# Acknowledgment to Reviewers of *Materials* in 2020

**DOI:** 10.3390/ma14030514

**Published:** 2021-01-21

**Authors:** Materials Editorial Office

**Affiliations:** MDPI AG, St. Alban-Anlage 66, 4052 Basel, Switzerland

Peer review is the driving force of journal development, and reviewers are gatekeepers who ensure that *Materials* maintains its standards for the high quality of its published papers. Thanks to the cooperation of our reviewers, in 2020, the median time to first decision was 14 days and the median time to publication was 35 days. The editors would like to express their sincere gratitude to the following reviewers for their precious time and dedication, regardless of whether the papers were finally published:
A. T. Ezhil, VilianKrzysztof, ZakowskiA. Toshimitsu, YokoboriKrzysztof, ŻamojćAamer, NazirKrzysztof, ZdunekAamir, JalilKrzysztof, ZiopajaAaron D., NicholasKrzysztof, ŻółtowskiAaron J., PungKuan-Cheng, ChiuAaron M., ForsterKuei-Chih, FengAaron Yu-Jen, WuKugler, SzymonAase, ReyesKui, JiaoAbbas, GhanbariKui, LuoAbbas, HaddadiKuldeep K., BansalAbbas, KhoshnoodKumar, AnupamAbbas, MadaniKun Viviana, TianAbbas, MohajeraniKun, ZhouAbbas, TaheriKunal, MishraAbbas, TamadonKunal, MondalAbbasali Taghavi, GhalesariKuniharu, UshijimaAbdalla, Abdal-hayKuo, YuanAbdel, El-kharbachiKuo-Cheng, HuangAbdelhafed, TalebKuorwel, KuorwelAbdelhamid, ElaissariKwangsuk, ParkAbdelkrem, EltaggazKwang-Yong, ChoiAbderrahmane, AyadiKyeong-Keun, ChoiAbdollah, BahadorKyeongsik, WooAbdollah, SabooriKyle Holmberg, ViningAbdulgalim, IsaevKyong Yop, RheeAbdulkarim, AmirovKyoung Youl, ParkAbdurizzagh, KhalfKyoung-Kyu, ChoiAbhay, KotnalaKyoung-Seok, MoonAbheetha, PeirisKyriaki, ManoliAbhijit, ChatterjeeKyriakos, KourousisAbhirup, DikshitKyung Hyun, AhnAbhishek K., MishraKyung Joon, ShinAbhishek, DasKyung-Ah, KwonAbhishek, MehtaKyunghoi, KimAbhishek, SharmaKyung-Min, KimAbishek B., KamarajKyung-San, MinAbishek, IyerKyung-Taek, KimAblyaz, TimirL’uboslav, StrakaAbm, ZakariaLadislav, CenigaAbolfazl, ShakouriLadislav, CvrčekAbshar, HasanLadislav, FalatAbu Ul Hassan Sarwar, RanaLadislav, KavanAbul B. M., SaifullahLaëtitia, MartyAbul Fazal M., ArifLai Mun, WongA-Cheng, WangLaia, HaurieAchim Walter, HasselLaichang, ZhangAdam Feliks, JunkaLaiming, JiangAdam, BieńkowskiLakshmi Prasanna, LingamdinneAdam, CwudzińskiLalita, SaharanAdam, GadomskiLanh Si, HoAdam, GąskaLara, RebaioliAdam, GlowaczLarissa, Moravcikova-GouveaAdam, GrajcarLarry, LessardAdam, JakubasLars Pilgaard, MikkelsenAdam, KulawikLars, BittrichAdam, MasłońLars-Åke, NäslundAdam, PiaseckiLasse, MurtomäkiAdam, PietrasLaszlo, FrazerAdam, StolarskiLászló, GáspárÁdám, TajtiLaszlo, KovacsAdam, WatrasLászló, LendvaiAdam, WisniewskiLaszlo, PeterAdam, ZielinskiLaszlo, TothAdeel, AfzalLászló, TrifAdela, NanoLaura Cristina, RusuAdele, CarradoLaura, BulgariuAdelino V., LopesLaura, D’alfonsoAdeyemi, AdesinaLaura, FarinaAdham, ElsayedLaura, FumagalliAdi, Ben-ArtzyLaura, GambariAdin, SternLaura, IopAdina Magdalena, MusucLaura, MorettiAdina, CoroabaLaura, OrianAdinel, GavrusLaura, PolitoAdithya, PeddapuramLaura, SalonenAditya Rio, PrabowoLaura, ŽalimienėAditya, MantriLaurel, SillerudAditya, WalvekarLaurence J., WalshAdnan Raheel, ShahLaurent Karim, BélandAdnan, YounisLaurent, LibessartAdolfo, Di FioreLaurentiu, SlatineanuAdrian Alexandru, SerbanoiuLavinia Cosmina, ArdeleanAdrian Ionut, NicoaraLavinia Petronela, CurecheriuAdrian, AugustyniakLavinia, TofanAdrian, BarylskiLazaros, TzounisAdrian, BurlacuLe, Quynh HoaAdrian, CirciumaruLe, ZhouAdrian, CurtoLéa, TrichetAdrian, DeaconuLea, ŽibretAdrian, NicoaraLeah, TolosaAdrián, Tenorio-AlfonsoLeandro, MaioAdriana Haydee, Martínez RegueroLech, CzarneckiAdriana Vulpoi, LazarLech, LichołaiAdriana, EstokovaLechosław, TuzAdriana, FilipLee Ann, Laurent-ApplegateAdriana, KovalcikLehui, LuAdriana, NowakLei, JinAdriana, Vargas-NordcbeckLei, Ka-MengAdriano, TroiaLei, MeiAdy, SuwardiLei, WangAerhan, KimLei, YuanAfriyanti, SumbojaLei, ZhangAfsaneh, ValizadehLeif, KariAfshin, HadipourLeila Jannesari, LadaniAgata, BorowikLeila, HashemianAgata, Jakóbik-KolonLeila, MomenzadehAgata, ŁamaczLeire, Ruiz RubioAgata, Podsiadły-PaszkowskaLeire, Ruiz-RubioAgata, PrzekoraLeixiao, YuAgata, SiwińskaLejeunes, StéphaneAgnė, KairytėLembit, KommelAgnès, GranierLeming, SunAgnes, KlarLena, Sundqvist ÖqvistAgnieszka J., NowakLenaic, LartigueAgnieszka, BrzózkaLenka, BodnárováAgnieszka, CzapikLenka, KucharikováAgnieszka, DąbrowskaLenka, ScheinherrováAgnieszka, DulskaLenuta, ProfireAgnieszka, Gadomska-GajadhurLeo J., McGillyAgnieszka, Gładysz-PłaskaLeon, KukiełkaAgnieszka, GubernatLeonard, AtanaseAgnieszka, JankowskaLeonardo, Bertolucci-CoelhoAgnieszka, JędrzejewskaLeonardo, CaprioAgnieszka, KochmańskaLeonardo, Di VenereAgnieszka, KrólickaLeonardo, OraziAgnieszka, KudelkoLeonardo, VelascoAgnieszka, KyziołLeonhard, HitzlerAgnieszka, Łękawa-RausLeonid M., KustovAgnieszka, PawłowskaLeonid V., LutsevAgnieszka, PiegatLeonid, BolotovAgnieszka, ŚlosarczykLeonid, SatrapinskyyAgnieszka, SzkliniarzLeonor, Santos-RuizAgnieszka, TercjakLeopoldo, FornerAgnieszka, TwardowskaLequien, FlorenceAgnieszka, WanagLeslaw, KyziolAgnieszka, WoszukLeszek Adam, DobrzanskiAgnieszka, ŻelaznaLeszek, CzechowskiAgostina, OreficeLeszek, ŁatkaAgostino, LauriaLeszek, WanatAgostino, MaurottoLeszek, ZaraskaAguinaldo, FraddosioLetizia, GiampietroAgustin, SalazarLeto-Aikaterini, TzivelekaAhamed, IrshadLex, ReiterAhmad, LabaniehLi (Licky), ZhuAhmad, MostafaLi, ChengAhmad, OmarLi, XiaoAhmed A., GheniLi, YangAhmed M., Abu-DiefLian Qiao, YangAhmed O., MoslehLiana, AnicǎiAhmed Ramadan, SuleimanLiana, MuresanAhmed Sabry, AfifyLianfeng, ZhaoAhmed Saleh, DalaqLiang, FanAhmed, Abdel-MohtiLiang, HongAhmed, AbumazwedLiang, LuoAhmed, AfifyLiang, TianAhmed, Al-RahmaniLiang, YanAhmed, ArafaLiangliang, ChengAhmed, ElhattabLianshe, FuAhmed, ElkaseerLianwen, DengAhmed, IbrahimLiberato, FerraraAhmed, JalalLibin K., BabuAhmed, MaamounLibor, BenesAhmed, MaaroufLibor, MrňaAhmed, NassrLibor, TopolářAhmed, OmarLiborio, CavaleriAhmed, TalaatLichong, XuAhmet, KertmenLidia, BaiamonteAhmet, TeberLidia, BanduraAida, NaghilouLidia, ReczekAikaterini K., AndreopoulouLidia, Stasiak-RóżańskaAikaterini, RogkalaLidia, ZurAikaterini, VarveriLidiia, KolzunovaAilar, HajimohammadiLidija, Andros DubrajaAimar, OrbeLidija, ĆurkovićAinhoa, RiquelmeLifeng, YanAiqin, WangLigia, MogaAira, MatsugakiLiguo, QinAires, CamõesLihong, SuAirong, XuLihua, LiAissa, BouaissiLihui, LangAissam, AiroudjLijun, ZhangAitana, TamayoLili, WuAitor, BeranoagirreLiliana, PorojanAitzol, LamikizLiliana, SachelarieAivaras, KareivaLiliang, OuyangAiying, WangLiliya, DunyushkinaAiyong, WangLily, PoulikakosAjay Giri Prakash, KottapalliLily, YangAjay Vikram, SinghLin, ChenAjay, KrishnamurthyLin, LiAjeet, KaushikLin, TianAjit, DhamdhereLin, ZhangAkbar Afaghi, KhatibiLina, StangvaltaiteAkhil, TayalLinda, Makovická OsvaldováAkihide, SaimotoLinda, ZottiAkihiko, FujiwaraLing Bing, KongAkihiko, KatoLing, RenAkihiro, NakataniLing, WangAkihito, HashidzumeLing, YifengAkiko, TsurumakiLing, ZhaoAkin, AkturkLingxin, ChenAkinola, OyedeleLingyun, YouAkinori, KuzuyaLinh T. B., NguyenAkio, NishimotoLinping, ZhaoAkira, IshigamiLinru, XuAkira, OtsukiLintao, BuAkira, TsuchiyaLinus Yinn Leng, AngAkira, YamadaLinxi, HouAkishige, HokugoLionel, MauriziAkitoshi, ShiotariLiping, LiuAkkapol, Suea-NgamLiqiang, WangAkram, AlhusseinLiqiu, ZhengAkshay, ChaudhariLiqun, NingAlaeddin Burak, IrezLis K., NanverAlain Vande, WouwerLisa E., PrevetteAlain, Gil Del ValLisa, BiasettoAlain, PercevalLisa-Marie, CzernuschkaAlaitz, Linares-UnamunzagaLisa-Marie, FallerAlaitz, ZabalaLisandra, MenesesAlalea, KiaLise, SandnesAlan Edward, TonelliLiu, HaifengAlan Keith, HellierLiudmila A., GorelovaAlan, TurnbullLiudmyla, SoldatkinaAlan, WemyssLiudvikas, UrbonasAlastair, MacLeodLiutauras, MarcinauskasAlbert E., PattersonLiuyang, ZhangAlbert, ArgilagaLivia Dana, BejuAlbert, Estrugo-DevesaLiviu, DutaAlbert, Rimola GibertLiwei D., GengAlbert, SabbanLjerka, Slokar BenićAlbert, SerràLluís, CasasAlbert. K. H., KwanLluïsa, EscodaAlberto Jesús, Perea MorenoLogesh, MathivathananAlberto N., AraújoLoic, HilliouAlberto S., De LeónLok, ShresthaAlberto V., PugaLong Y., ChiangAlberto, AssirelliLong, XinAlberto, BarrosoLongbiao, LiAlberto, BattistelLongfei, WuAlberto, CampagnoloLongjun, DongAlberto, ConcellonLong-Sheng, HuangAlberto, De BiaseLongyu, LiAlberto, FabriziLoredana, ContrafattoAlberto, FalcóLoredana, ProtesescuAlberto, FracheLorella, MarinucciAlberto, FraileLorena Freitas, LorenaAlberto, García-PeñasLorena, DeleanuAlberto, GirlandoLorena, Garcia CucalonAlberto, GregoriLorena, PardoAlberto, Hernández CreusLorena, ZichellaAlberto, LagazzoLorenza, DraghiAlberto, López OrtegaLorenza, MaddalenaAlberto, MannuLorenzo S., CaputiAlberto, MonsalveLorenzo, AbateAlberto, Rodríguez-ArchillaLorenzo, BigianiAlborz, ShokraniLorenzo, LisuzzoAled Deakin, RobertsLorenzo, VannozziAlejandro González, OriveLoreta, Tamašauskaitė-TamašiūnaitėAlejandro, Burgos-CaraLori C., BassmanAlejandro, EnfedaqueLotfi, BessaisAlejandro, Sánchez-PostigoLothar, SpießAlejandro, VegaLouis Ngai Sum, ChiuAlejandro-Miguel, Sanchez-de-la-MuelaLoukya, BoddapatiAleksandar M., MitrašinovićLourdes, FrancoAleksandar, KondinskiLovre, Krstulović-OparaAleksander, HejnaLu, ShaoAleksander, KowalskiLu, SunAleksander, LisieckiLu, YuAleksander, MarekLu, ZhangAleksandr P., AmosovLu, ZhengAleksandr S., VoznesenskiiLubica, HallmannAleksandr V., IvanishchevLubinda, WalubitaAleksandr V., ShvidchenkoLublóy, Éva EszterAleksandr, KlimovĽubomír, ČaplovičAleksandr, OreshonkovĽubomír, KubíkAleksandr, PshykLubomir, MedveckyAleksandr, SakhnevychLubomira, ToshevaAleksandr, ZinovievLubos, DanisovicAleksandra, DrygałaLubos, KlocAleksandra, Grząbka-ZasadzińskaLubos, KristakAleksandra, Kostrzanowska-SiedlarzLuboš, PazderaAleksandra, KrampikowskaLubov, SnegurAleksandra, Kuryłowicz-CudowskaLuc, CourardAleksandra, Palatyńska-UlatowskaLuc, LebeauAleksandra, RadtkeLuc, St-PierreAleksandra, RybakLuca, BertollaAleksandra, ŚwierczyńskaLuca, BoccarussoAleksandra, SzkudlarekLuca, CeseracciuAleksandra, WajdaLuca, ColliniAleksandra, ZielinskaLuca, De StefanoAleksei V., AlmaevLuca, Di PalmaAleksei, ObrosovLuca, EspositoAleksej, ZarkovLuca, FacconiAleksey A., VedyaginLuca, FalzoneAleksey E., PestovLuca, FerrantinoAleksey G., LipnitskiiLuca, FiorilloAleksey, Anatolievich ZakharenkoLuca, GiachettiAleksey, MaksimkinLuca, GiorleoAleksey, RogovLuca, LavagnaAleksey, VishnyakovLuca, MascarettiAlena, KalendováLuca, MeddaAlena, MichalcováLuca, PezzatoAlena, PietrikovaLuca, RigamontiAlena, SičákováLuca, SchenatoAlena, VimmrováLuca, SgambiAlena, ZemanováLuca, SorrentinoAlenka, VeselLuca, SpiridigliozziAleš, ChválaLuca, TestarelliAles, IglicLuca, TetiAles, MizeraLuca, ZoliAleš, StražeLucanos, StrambiniAlessandra, FuscoLucas A., HofAlessandra, LuccheseLucas, CarettaAlessandra, MobiliLucia, BaldinoAlessandra, StacchiottiLucia, D’accoltiAlessandro Fraleoni, MorgeraLucia, KnapcikovaAlessandro P., FantilliLucia, LattanziAlessandro, BellucciLucia, LeonatAlessandro, BistolfiLucia-Antoneta, ChicosAlessandro, CavalloLucian A., BlagaAlessandro, CerutiLucian, ConstantinAlessandro, ChiadòLucian, LazarescuAlessandro, ChiolerioLucian, MihaescuAlessandro, Di MarcoLucian, Pislaru-DanescuAlessandro, FantoniLuciana, AlgieriAlessandro, FrancoLuciano, Di MaioAlessandro, GalendaLuciano, LambertiAlessandro, GambardellaLuciano, PacificiAlessandro, GiorgettiLuciano, VelardiAlessandro, GrazziniLucie, BartoňováAlessandro, GuastoniLucie, HympánováAlessandro, LatiniLucio, LittiAlessandro, MartucciLucio, MaragoniAlessandro, MeduriLucio, MeloneAlessandro, NotaLucio, NobileAlessandro, PatelliLucja, Dybowska-SarapukAlessandro, PegorettiLucjan, ChmielarzAlessandro, PistoneLucjan, JacakAlessandro, PratesiLucjan, SniezekAlessandro, TonacciLucrezia, AversaAlessandro, ZonaLucy, ArmitageAlessia, ArtesaniLucyna, JaworskaAlessia, Le DonneLudek, HellerAlessio, CascardiLudek, JelinekAlessio, LampertiLudger, WeberAlessio, MezziLudmila, KučerováAlessio, MontiLudovic, ColombeauAlessio, SilvelloLudovic, JasonAlessio, VernaLudovic, SamekAlex Ching Tai, NgLudovica, CacopardoAlex J., YuffaLudovica, ParisiAlex M. H., ChauLudovica, RovattiAlex M., ValmLudovico, MegaliniAlex V., TrukhanovLudwig, CardonAlex, Cordero-CasanovasLuidmila, YakimovaAlex, JordanLuigi Pietro Maria, ColomboAlex, MontagneLuigi Vito, StefanelliAlexander A., BalandinLuigi, CalabreseAlexander A., GromovLuigi, CoppolaAlexander Alexandrovich, LebedevLuigi, De NapoliAlexander E., MayerLuigi, De NardoAlexander E., SytschevLuigi, FiorinoAlexander F., MasonLuigi, FortunaAlexander G., TskhovrebovLuigi, La SpadaAlexander M., GaskovLuigi, ManfrediAlexander M., KorsunskyLuigi, StagiAlexander M., LererLuis A., AngurelAlexander M., SamoylovLuis A., MataAlexander M., VolodinLuís Carlos, Ramos Nunes Pinto FerreiraAlexander N., MitropoulosLuis Carlos, Sandoval-HerazoAlexander P., SvintsovLuís F., MendesAlexander S., AntonovLuis Felipe, Verdeja GonzálezAlexander S., BrandLuis Filipe Almeida, BernardoAlexander S., ChausLuís Filipe, TeixeiraAlexander S., KrylovLuis Javier, Del ValleAlexander S., NovikovLuis M., Garcia-RaffiAlexander V., Artem’evLuís Manuel, AlvesAlexander Yu., ChuryumovLuís Miguel P., DurãoAlexander Yu., IvannikovLuis N. López, De LacalleAlexander Yu., KuntsevichLuis Norberto, López De LacalleAlexander, BakulinLuís Picado, SantosAlexander, CroyLuís Pinto, Da SilvaAlexander, DonchevLuis, BañónAlexander, EfimovLuís, BernardoAlexander, EliseevLuis, CoelhoAlexander, FedorchenkoLuís, CorreiaAlexander, GagarinLuís, Giner-TarridaAlexander, GottwaldLuis, Guerra RosaAlexander, HartmaierLuis, MesquitaAlexander, HeißLuís, PinhoAlexander, KaramanovLuís, Pinto Da SilvaAlexander, KirillovLuís, ProençaAlexander, KomissarovLuis, Rodríguez-LorenzoAlexander, LaptevLuis, Saucedo MoraAlexander, LuntLuís, SousaAlexander, MalikovLuís, VilhenaAlexander, MarrasLuis, YermánAlexander, MartinLuisa Maria, FerreiraAlexander, McLeanLuisa, CarvalhoAlexander, MorganLuisa, MedinaAlexander, ObraztsovLuisa, MolariAlexander, OvchinnikovLuisa, PaniAlexander, PatselovLuisa, PilanAlexander, PestovLuisa, RoveroAlexander, RyabtsevLuiz Antonio Pereira, De OliveiraAlexander, SafonovLuiz, PereiraAlexander, SamardakLuka, PavicAlexander, SamokhvalovLukas, RichteraAlexander, ShorokhovŁukasz, ChomątekAlexander, SidorenkoŁukasz, DrobiecAlexander, TikhonravovŁukasz, GołekAlexander, ToikkaŁukasz, Jan LaskowskiAlexander, Udal’tsovŁukasz, KonatAlexander, VanetsevŁukasz, KrawczykAlexander, VorontsovŁukasz, ŁapajAlexander, VorozhtsovŁukasz, LaskowskiAlexander, WelleŁukasz, ŁopusiewiczAlexandr A., ZaitsevLukasz, MendeckiAlexandr, BelosludtsevŁukasz, NagiAlexandr, KnápekŁukasz, PejkowskiAlexandr, SadovnikovŁukasz, PiskorskiAlexandr, StupakovŁukasz, PiszczykAlexandra Ripszky, TotanLukasz, RadosinskiAlexandra, AlexandriLukasz, SadowskiAlexandra, GoryaevaŁukasz, SkarżyńskiAlexandra, InayatŁukasz, ŚlusarczykAlexandra, Muñoz-BonillaLukasz, SójkaAlexandra, PapadopoulouŁukasz, ŚwięchAlexandra, PulyalinaŁukasz, SzeleszczukAlexandra, SzczesŁukasz, TabiszAlexandra, ZamboulisŁukasz, ŻyłkaAlexandre Cunha, BastosLulu, LiAlexandre, AnesiLuminita, AmarandeAlexandre, VivetLuminita, MarutescuAlexandre, YokochiLuminita, ParvAlexandros K., NikolaidisLuo, LiuAlexandru Mihai, GrumezescuLutfi, AgartanAlexandru, EnescaLydia, SobotovaAlexandru, MesterLyudmila V., ParfenovaAlexei N., SuchkovLyudmila, Bel’skayaAlexei V., DubM. Reza, PouranianAlexei, KuzminM. Shadi, MohamedAlexei, VinogradovMaarten, ArnstAlexey A., TsyganenkoMachel, MorrisonAlexey A., VereshchakaMaciej Robert, DutkiewiczAlexey B., NadyktoMaciej, DobrzyńskiAlexey F., KosolapovMaciej, DyziaAlexey G., KolmakovMaciej, GagatAlexey I., ChizhikMaciej, GieradaAlexey L., GlazovMaciej, JaworskiAlexey L., IordanskiiMaciej, KalinowskiAlexey N., KuznetsovMaciej, KujawaAlexey V., PovolotskiyMaciej, MaliszewskiAlexey, BeskopylnyMaciej, MotykaAlexey, BobrovskyMaciej, NiedostatkiewiczAlexey, BubnovMaciej, PietrzykAlexey, LipatovMaciej, PodgórskiAlexey, LukoyanovMaciej, RadzieńskiAlexey, MikhaylovMaciej, RoskoszAlexey, PorubovMaciej, SkotakAlexey, ProsviryakovMaciej, SowaAlexey, SalimonMaciej, SzelągAlexey, SoloninMaciej, ZubkoAlexey, VolkovMădălina, DumitriuAlexey, VoloshinMadalina, PandeleAlfiero, LeoniMadankara K., NivedyaAlfonso, Fernández-CanteliMadhab, PokhrelAlfonso, Navarro-LópezMadhukar, SomireddyAlfonso, San-MiguelMadis, RatasseppAlfred, TeischingerMaedeh, AmirpourAlfredas, LaurinaviciusMafalda, GuedesAlfredo M. P. G., DiasMafalda, LaranjoAlfredo, CapparielloMagdalena Daria, VaverkováAlfredo, RoncaMagdalena Valentina, LunguAli A., JasimMagdalena, BrodaAli Cemal, BenimMagdalena, DobiszewskaAli H., ReshakMagdalena, GacaAli M., AbdelhafeezMagdalena, GierszewskaAli, Abbaspour TamijaniMagdalena, JanusAli, Abdel-MageedMagdalena, KrólAli, Abdul-AzizMagdalena, KwiatkowskaAli, BagheriMagdalena, LaskowskaAli, BehnoodMagdalena, LeśniakAli, BelhocineMagdalena, Lukaszewska-KuskaAli, Borzabadi-FarahaniMagdalena, MachnoAli, EftekhariMagdalena, Makarska-BialokozAli, FahemMagdalena, MałeckaAli, FarajpourMagdalena, Niemczewska-WojcikAli, H., TayebMagdalena, OćwiejaAli, JamshidiMagdalena, PalaczAli, KarparvarfardMagdalena, RajczakowskaAli, PaknahadMagdalena, RogulskaAli, TabeiMagdalena, Rozmus-GórnikowskaAli, TalimianMagdalena, RuckaAli, ZaidiMagdalena, SobiesiakAli, ZolaliMagdalena, SzutkowskaAliakbar, HassanpouryouzbandMagdalena, ZiąbkaAliaksandr, ShaulaMaged F., BekheetAlice A. K., KingMaged, YoussefAlice, HospodkováMagnus, AndersonAlicia Loreto, García CostaMagnus, NeikterAlicia, Gomis-BerenguerMahdavi, MohammadAlicja, Kazek-KęsikMahdi, BodaghiAlicja, KrellaMahdi, DamghaniAlicja, NejmanMahdi, KeramatikermanAlina Adriana, MineaMahdi, KioumarsiAlina Gabriela, RusuMahdi, TakaffoliAlina Ioana, BadanoiuMahesh, PeddigariAlina, BarbulescuMahesh, VisheAlina, KaletaMahfooz, SoomroAlina, ManshinaMahnoush, BabaeiAlina, MineaMahroo, FalahAlina, PrunaMai Thanh, NguyenAlina, VladescuMaider, MuroAlireza Fadavi, BoostaniMaja Zebic, AvdicevicAlireza R. DamanpackMaja, AntunovićAlireza V., AmirkhiziMaja, KępniakAlireza, FarzampourMaja, VončinaAlireza, JavadianMajid G., RamezaniAlireza, NaseriMajid, AliAlireza, SassaniMajid, AstanehAlireza, TabatabaeiMakarona, EleniAlireza, TofangchiMakoto, HandaAlisa, RupenyanMakoto, HashimotoAlla, SalminaMakoto, KobayashiAlla, SologubenkoMakoto, YamamotoAllam I. S., ArdahMaksim, AntonovAllan, ZarembskiMaksim, BoldinAllison, Hess-DunningMaksim, StarykevichAlmir, GazizovMaksym, RybachukAlmudena, BenítezMakusu, TsutsuiAlmudena, RivadeneyraMalgorazata, MakowskaAlok, KumarMałgorzata, Adamczyk-HabrajskaAlvaro, Gallo CordovaMałgorzata, Geszke-MoritzÁlvaro, González-VilaMałgorzata, GołaszewskaÁlvaro, PicazoMałgorzata, Gra̧dzka-DahlkeAlvaro, RidruejoMalgorzata, HolynskaÁlvaro, Rodríguez GonzálezMalgorzata, HolyńskaAlwar, RamaniMałgorzata, Jedrzejewska-SzczerskaAlwin, DausMalgorzata, KarolusAlyaa, MohammedMałgorzata, KidaAmaia Soto, BeobideMałgorzata, LenartAmaia, JiménezMałgorzata, LinekAmaia, SantamaríaMałgorzata, MaciejewskaAmalia, GordanoMałgorzata, NorekAmalia, JiménezMałgorzata, PająkAmalia, MesarosMałgorzata, SzymiczekAmalraj, MarshalMalgorzata, UlewiczAmalraj, Peter AmalathasMałgorzata, WarmuzekAmanda C., MillsMałgorzata, WolskaAmanda R., KrauseMalik, YahiaouiAmanda, GibsonMalik, Zaka UllahAmandeep, AroraMalina, DagmaraAmandine, MiksicMallardo, VincenzoAmanmyrat, AbdullayevMallem Siva Pratap, ReddyAmborish, BanerjeeMallesh, KurakulaAmedeo, ManfrinManabu, EnokiAmelia Elena, BocîrneaManabu, HagiwaraAmeri Shideh, KabiriManabu, KanazawaAmerico, ScottiManas, SutradharAmerigo, GiudiceManel, Da SilvaAmeur, LouhichiManfred, DunkyAmey, KhanolkarManfred, WagnerAmeya, NarkarManfred, WeihnachtAmin, Babaei-GhazviniMangialardi, TeresaAmin, BahramiMan-Hoe, KimAmin, ChegenizadehManickam, MinakshiAmin, EbrahimiManik, BarmanAmin, ShavandiManing, LiuAminhossein, JahanbinManita, DangolAmir Masoud, PourrahimiMan-Jong, LeeAmir, ChamaaniManjusha, BattabyalAmir, DehghanManoj Atmaramji, UpadhyaAmir, DehghanghadikolaeiManoj K., MahapatraAmir, HassanpourManoj Kumar, MahataAmir, KordijaziManoj, KhandelwalAmir, MosaviManoli M., ZubiturAmir, TabakovicManolis, ManosAmirali, ShamsolhodaeiManomi, PereraAmirhesam, AmerinatanziManon, BonvaletAmirkianoosh, KianiManos, VlasiouAmirmansoor, AshrafiMansoo, JounAmirreza, AghakhaniManuel A., Fernández-RuizAmirreza, KhodadadianManuel Alejandro, Pedreño RojasAmit Kumar, SahaManuel Antonio, Diaz-PerezAmit Kumar, SarkarManuel Checa, GómezAmit, MunshiManuel Filipe Pereira Cunha Martins, CostaAmit, NautiyalManuel Francisco Costa, PereiraAmit, PawbakeManuel J., Lis AriasAmit, SharmaManuel J., Pérez-MendozaAmm Sharif, UllahManuel Jesús, Hermoso-OrzáezAmmar, YahiaManuel Marques, FerreiraAmmu, PrhashannaManuel Montenegro, CabreraAmnon, ShirizlyManuel V., RamalloAmod A., OgaleManuel, AngstAmr, AbdelkaderManuel, AurelianoAmr, NadaManuel, Cruz-YustaAmrita, BasakManuel, DominguezAn, LiangManuel, Ferrandez-VillenaAna B., LagoManuel, FreitasAna Cristina, CorreiaManuel, Gutierrez RamirezAna Cristina, FreireManuel, JordánAna Francisca, BettencourtManuel, Peñas-GarzónAna Isabel, Fernández-AbiaManuel, PiñeroAna Isabel, Nicolas-SilventeManuel, RegueiroAna Laura Luna, BarronManuel, Rodriguez-MartinAna Lúcia, HorovistizManuel, Sierra-CastanerAna Luísa, Daniel-da-SilvaManuel, Toledano-OsorioAna M., BeltranManuel, Uceda-RodríguezAna Margarida, UrbanoManuel, VazquezAna María, CamachoManuel, VieiraAna Maria, Santos-CoquillatManuela Sonja, KillianAna Meria, CastroManuela, PacellaAna Paula Betencourt, Martins AmaroManuela, RebaudengoAna Paula, PiedadeManuela, RomagnoliAna Pilar, Valerga PuertaManuela, SchiekAna Rita, Costa-PintoMaodie, WangAna Santos-CoquillatMaozhou, MengAna Teresa, Silva Cardoso BrandãoMara, CamaitiAna, AlcudiaMara, MihaiAna, ÁlvarezMāra, PilmaneAna, Briga-SáMarat, DosaevAna, CazacuMarat, EseevAna, CuestaMarat, GafurovAna, Espinel-IngroffMarc, Marín-GenescàAna, EvangelistaMarc, SnapperAna, García DiezMarc, WidenmeyerAna, HeitorMarcela E., Barbinta-PatrascuAna, IvaniševićMarcela E., TrybułaAna, JosanMarcela, PekarcikovaAna, MessiasMarcela, PokusováAna, Morales-RodríguezMarcela, SocolAna, PavlovicMarcella, BiniAna, PilipovićMarcello, BertoAnabela Baptista, PaulaMarcello, IasielloAnagh, DeshpandeMarcello, MaddaloneAnahita, EmamiMarcello, MerliAnaïs, Pitto-BarryMarcello, RomagnoliAnamaria, BechirMarcelo, De MouraAna-Maria, GalanMarcin Andrzej, GolabczakAna-Maria, PutzMarcin Henryk, StruszczykAnamarija, FarkašMarcin J., JewiarzAnandram, VenkatasubramanianMarcin K., WidomskiAnargyros, KarakalasMarcin, AbramskiAnastasia V., VasilevichMarcin, BorowiczAnastasia, MikhaylovskayaMarcin, DudekAnastasia, MitseaMarcin, GajewskiAnastasia, StamatiouMarcin, GnybaAnastasiia, KrushynskaMarcin, GodzierzAnastasios, GotziasMarcin, GórnyAnastasios, RaptisMarcin, GórskiAnastasios, TasiopoulosMarcin, GrabaAnatoli, PopovMarcin, GrabowskiAnatolijus, EisinasMarcin, HoffmannAnatoliy, KovalevMarcin, JasiewiczAnatoly F., ZatsepinMarcin, JesionekAnatoly N., ReshetilovMarcin, KamińskiAnatoly, BelonoshkoMarcin, KobieluszAnatoly, FilippovMarcin, KochanowiczAnatoly, ZinchenkoMarcin, KondrackiAnaxagoras, ElenasMarcin, KrajewskiAnbharasi, VanangamudiMarcin, KremieniewskiAnca Niculina, CadinoiuMarcin, KujawaAnca, BirsanMarcin, ŁapińskiAnca, CojocaruMarcin, LebiodaAnca, DumbravaMarcin, MadejAnca, MazareMarcin, MałekAnca, RoseanuMarcin, MasłowskiAnca, StanaMarcin, MaździarzAnders, KaestnerMarcin, NabiałekAndra-Cristina, DumitruMarcin, SosnowskiAndrás György, NémethMarcin, StawarzAndraž, KocjanMarcin, StienssAndraz, SuligojMarcin, StrąkowskiAndré Dias, MartinsMarcin, SylwestrzakAndré Ferreira, MoreiraMarcin, SzczechAndré R. R., SilvaMarcin, SzczepańskiAndre, BaldermannMarcin, WachowskiAndre, CastroMarcin, WekwejtAndré, FurtadoMarcin, WłochAndre, HaelsigMarcin, ZastempowskiAndre, McDonaldMarco A., OrtenziAndré, MeloMarco Alexandre, RibeiroAndrea Francesco, CiuffiniMarco Ludovico, MarquesAndrea Pietro, ReverberiMarco, Actis GrandeAndrea, ArnoldMarco, AlfanoAndrea, AtreiMarco, AnniAndrea, BalliniMarco, BaronAndrea, BenedettiMarco, BassaniAndrea, BernasconiMarco, BernasconiAndrea, BrotzuMarco, BobingerAndrea, CollinaMarco, BonoperaAndrea, DorigatoMarco, CaniatoAndrea, EhrmannMarco, CannasAndrea, GersonMarco, CicciùAndrea, GrazianiMarco, CiveraAndrea, GruttadauriaMarco, ColomboAndrea, MaioMarco, CorradiAndrea, MartinelliMarco, DomaneschiAndrea, MescolaMarco, GigliottiAndrea, MezzettaMarco, GovoniAndrea, MichelettiMarco, Lamperti TornaghiAndrea, MontessoriMarco, LaurentiAndrea, Navarro-QuezadaMarco, LezzeriniAndrea, NicolasMarco, LiebscherAndrea, OrsiniMarco, LollobrigidaAndrea, PacificiMarco, MartinoAndrea, PaolellaMarco, MascittiAndrea, PetrellaMarco, MilanesioAndrea, PorzionatoMarco, MiliucciAndrea, ProtoMarco, MiniaciAndrea, PucciMarco, MontemurroAndrea, RoccuzzoMarco, PaolinoAndrea, SchiratoMarco, PasettoAndrea, ScribanteMarco, PepeAndrea, SiliMarco, PirolaAndrea, SimonMarco, PoianaAndrea, SorrentinoMarco, PortelliAndrea, SpagnoliMarco, RaceAndrea, TerenziMarco, RossiAndrea, TridelloMarco, SacchiAndrea, ZoccaMarco, SalerAndreas Max, PabstMarco, SalernoAndreas, DelimitisMarco, SchowalterAndreas, EndruweitMarco, StollerAndreas, ErlebachMarco, TallaricoAndreas, HoffmannMarco, TatulloAndreas, HornigMarco, ToiaAndreas, KandelbauerMarco, ZannottiAndreas, KulovitsMarcos A. P., MartinsAndreas, LennartssonMarcos G., AlbertiAndreas, LeuteritzMarcos, De Oliveira JuniorAndreas, MautnerMarcos, DiazAndreas, MeyerMarcos, RodriguesAndreas, StaschMarcos, Rodríguez MillánAndreas, TortschanoffMarcus, AßmusAndreas, TreuMarcus, BjörlingAndreas, TsakalofMarcus, DymondAndreas, WirtzMarcus, JahnAndreea L., Chibac-ScutaruMarcus, RennhoferAndreea, CostasMarcus, SchreinerAndreea, DutuMarek, BrzezinskiAndreea, GrozaMarek, GucwaAndreea, MateiMarek, HawrylukAndrei V., KovalevskyMarek, IsbrandtAndrei V., MostovshchikovMarek, IwańskiAndrei V., PetukhovMarek, KalenikAndrei V., ShevelkovMarek, KobylańskiAndrei Vasile, NastutaMarek, KoutnýAndrei Victor, SanduMarek, KrawczukAndrei, ArtemevMarek, LechmanAndrei, CrisanMarek, MagdziakAndrei, EgorinMarek, MajAndrei, JitianuMarek, MarcinekAndrei, NovikovMarek, MołczanAndrei, PostnikovMarek, MuzykAndrei, PotapovMarek, NerudaAndrei, RotaruMarek, NowickiAndrei, SarbuMarek, PagáčAndrei, ShishkinMarek, PiątkowskiAndrei, SurženkovMarek, PokornyAndrei, SurzhenkovMarek, PolanskiAndrei, TarasovMarek, PrajerAndreia, OliveiraMarek, PszczolaAndreii S., KritchenkovMarek, SciazkoAndrej P., NazarovMarek, SkłodowskiAndres Fernando, ClarensMarek, SzkodoAndrés J., PrietoMarek, TarrasteAndres Sanz, GarciaMarek, UrbanskiAndrés, DíazMarek, WęglowskiAndres, GodoyMarek, WisniewskiAndres, Juan-ValdesMarek, WojtaszekAndres, OsvetMargaret J., SobkowiczAndresa, BaptistaMargaret, FreyAndrew (Chihpin), ChuangMargaret, GradovaAndrew B., CrollMargarita A., GoldbergAndrew B., SchenkelMargarita G., IsaenkovaAndrew C. E., ReidMargarita, ChizhAndrew D. M., CharlesMargarita, Gomez EscaladaAndrew I. M., GreerMargarita, GrozevaAndrew J., PascallMargarita, Vega HolmAndrew S., PaluchMargherita, TumedeiAndrew S., VoylesMargit L. H., RiisAndrew, GrygućMargit, SchulzeAndrew, KloseMargo, StaruchAndrew, RuysMari, KoikeAndrew, SpowageMaria A., GoulaAndrew, SwindleMaría A., Pérez-HerreroAndrew, ThomasMaria Addolorata, BonifacioAndrew, YostMaria Alba, MartinAndrey E., KrauklisMaria Alzira Pimenta, DinisAndrey Georgievich, PaulishMaría Angeles, PérezAndrey I., BazlovMaría Antonia, HerreroAndrey O., ZhigachevMaría Belén, Villacampa NaveracAndrey S., MereshchenkoMaria Carmen, Román-MartínezAndrey V., FilippovMaria Carmo, LançaAndrey, AleshinMaria Caterina, GiordanoAndrey, BaydinMaria Chiara, CavalliAndrey, BelyakovMaria Chiara, SportelliAndrey, FilippovMaria Concepción, CiveraAndrey, GnedenkovMaria Cristina, BonferoniAndrey, KistanovMaria Cristina, TanziAndrey, KoptyugMaría D., CouceAndrey, LiderMaria Daniela, StelescuAndrey, MedvedevMaría De Las Nieves, GonzálezAndrey, MiroshnichenkoMaria De Lourdes, PereiraAndrey, NoskoMaría Dolores, AvilésAndrey, OsipovMaría Dolores, Petit-DomínguezAndrey, PankratovMaria Dolores, Rubio-CintasAndrey, PoddelskyMaria Elena, De Cos GómezAndrey, PozdniakovMaria Elisa, TataAndrey, PryamikovMaria Elisabete C. D., Real OliveiraAndrey, SarikovMaria Erminia, GenoveseAndrey, TumarkinMaria Francesca, SfondriniAndrey, YurkovMaria G., ChernyshevaAndrey, ZuevMaria Giovanna, GandolfiAndrii, KostryzhevMaria Giulia, CristofaroAndriy, KvashaMaria Goreti, FernandesAndryushin Konstantin, PetrovichMaria Helena, FernandesAndrzej Jarosław, PanasMaria Helena, FigueiralAndrzej, AmbroziakMaria Icíar Arteagoitia, CalvoAndrzej, BrudnickiMaría Isabel, Prieto BarrioAndrzej, ChmielewskiMaria J., MilewskaAndrzej, ChudzikiewiczMaria Jacinta, SantosAndrzej, CzerwinskiMaría Jesús, Durán-PeñaAndrzej, DziedzicMaría Jesús, FernándezAndrzej, GarbaczMaría Jesús, Morales CondeAndrzej, GłuchowskiMaria Jesús, Morales-CondeAndrzej, KatuninMaría Jesús, SuárezAndrzej, KiełbusMaria Josè, Lo FaroAndrzej, KomorekMaría José, Mancheño RealAndrzej, KrasinskiMaría José, Suárez-LópezAndrzej, KubitMaría José, Valero-RomeroAndrzej, KurekMaria L. Almoraima, GilAndrzej, LewandowskiMaria L., CarreonAndrzej, ŁukaszewiczMaria Laura, De BellisAndrzej, MamalaMaria Laura, ParisiAndrzej, MatrasMaria Laura, TumminoAndrzej, MiklaszewskiMaria Lúcia M. F. S., SaraivaAndrzej, OkuniewskiMaria Luisa Della, RoccaAndrzej, OsuchMaría Luisa, García-RomeuAndrzej, PuszkaMaría Luisa, MarínAndrzej, SikoraMaría M., AlonsoAndrzej, SiomaMaria Magdalena, PastorAndrzej, SkrzypczykMaria Natividad, Gomez CerezoAndrzej, ŚlebarskiMaria Novella, RomanelliAndrzej, SmoleńMaria P., SokolovaAndrzej, StefanikMaria Paola, BraccialeAndrzej, TrytekMaria Paola, LudaAndrzej, WasiakMaria Rosaria, BoniAndrzej, WieczorekMaria Sameiro T., GonçalvesAndrzej, ZielinskiMaria Teresa, Blanco-ValeraAndy Wai Kan, YeungMaría Teresa, Senz DiezAndy, ChetwyndMaria Teresa, TodaroAndy, IngramMaria Valentina, DinuAneta, BartkowskaMaria Victoria, BiezmaAneta, KrzyzakMaria Vittoria, DiamantiAneta, Nowak-MichtaMaria, ArreguiAneta, PetelskaMária, BehúlováAnette, MüllerMaría, CanillasAng-Chen, TsaiMaria, CinefraÁngel De La, Rosa VelascoMaria, ContaldoAngel Miguel, Pitarch RoigMaria, DatchevaÁngel V., DelgadoMária, DománkováAngel, BarrancoMaria, Gamella CarballoÁngel, Díaz-OrtizMaria, GazdaÁngel, Morales-GarcíaMaria, HarjaÁngel, MuñozMaria, IgnatAngel, OrteMaria, KalivaAngel, Perez Del PinoMaria, KingAngel, RodriguezMaria, KoyioniÁngel, Rodríguez SaizMaria, LeżańskaÁngel, Serrano-ArocaMaria, LosurdoAngela P., CazzollaMaria, Luisa GrilliAngela, AgostianoMaria, MarinescuAngela, AndrzejewskaMaria, MeniniAngela, De BonisMaria, MilanovaAngela, Di BaldassarreMaria, MironovaAngela, Lo MonacoMaria, ModicaAngela, MalaraMaria, MuchaAngela, Sastre-SantosMaria, NetoAngela, ScalaMaria, NikolovaAngela, StaicuMaria, PantanoAngeles G., De La TorreMaria, PapastergiouAngeles, BlancoMaria, PopAngelika, KmitaMária, PorubskáAngeliki, BrouzgouMaría, Prados-PrivadoAngeliki, PapalouMaria, RapaAngelina, AngelovaMaria, RaposoAngélique, SourMaria, RichertAngelo Marcello, TarantinoMaria, RichettaAngelo, AlbiniMaria, RouliaAngelo, CasagrandeMaria, RussoAngelo, MullaliuMaria, RutkowskaAngelo, PutignanoMaria, SakovskyAngelo, TagliettiMaria, SarapultsevaAngelo, TroedhanMaria, SvedaAngelo, ZavattiniMaria, TaxiarchouAnge-Therese, AkonoMaria, ZentkovaAnghel, CernescuMaria-Carmen, AsensioAngus K. F., CheungMariacecilia, PasiniAnh D., VuMaria-Daniela, StelescuAnh Kiet, TieuMaria-Ioana, PastramaAnh Nguyen, VanMaria-Loredana, SoranAnicuta, StoicaMarialucia, GalloriniAniela, PopMarialuigia, RaimondoAniello, CostantiniMarialuisa, GentileAniello, RiccioMarialuisa, TorreAnil Kumar Reddy, PoliceMariamelia, StanzioneAnil Kumar, JonnalagaddaMarian Nicolae, VeleaAnil, BanerjeeMarian, DrienovskyAniruddh Jagdish, KhannaMarian, HettiaratchiAniruddh, DasMarián, KučeraAnişoara, CîmpeanMarian, LehockyAnita, CatapanoMarián, LehockýAnita, KwaśniewskaMarian, MiculescuAnita, Olszówka-MyalskaMarián, PalcutAnita, TarbukMarian, ŠofrankoAnja, LodeMarian, ZaborskiAnja, NoheMariana Domnica, StanciuAnja, WinklerMariana, BraicAnkica, ŠarićMariana, BusilaAnkit, RochaniMariana, KunevaAnkur, BajpaiMariana, MarinAnkur, SharmaMariana, VarnaAnn, RajnicekMariangela, LombardiAnna A., OkunkovaMariangela, LonghiAnna D., ZervakiMariangela, QuartoAnna Janina, DolataMarianna, BarbalinardoAnna M., OsyczkaMarianna, GniadekAnna, BacarditMarianna, PannicoAnna, BelcarzMarianna, RinaldiAnna, BielenicaMarianna, TriantouAnna, BienMariano, Fernandez FairénAnna, BiernasiukMaria–Styliani, VoutetakiAnna, Bratek-SkickiMariateresa, GuadagnuoloAnna, BulingMariateresa, LettieriAnna, CastaldoMaricica, PacurariAnna, Chomicz-KowalskaMarie Claude, ClochardAnna, ChurakovaMarie E., RoyAnna, DanihelováMarie Hubálek, KalbáčováAnna, DrabczykMarie, KvapilovaAnna, GancarczykMarie, SalguesAnna, GoiMarie, SejkorováAnna, GrosserMarie-Agathe, CharpagneAnna, HalickaMarielle, GueguenAnna, KhlyustovaMarietta, HerrmannAnna, KonstanciakMariia V., NutskovaAnna, KornevaMarija, NedeljkovićAnna, KowalewskaMarija, StjepanovićAnna, Kowalik-KlimczakMarija, StojmenovićAnna, KozakiewiczMarija, VaicieneAnna, Krztoń-MaziopaMarijana, Hadzima-NyarkoAnna, KusiorMarijana, SerdarAnna, LewinskaMarijo, BuzukAnna, MarzecMarilena, VlachouAnna, MasekMarilés, Bonet-AracilAnna, MłynarczykowskaMarin, MicutzAnna, MorozovaMarin, UgrinaAnna, NocivinMarina A., MaleevaAnna, PawełczykMarina A., VolosovaAnna, PrnováMarina Consuelo, VitaleAnna, RudawskaMarina G., KhmelevaAnna, SiekierkaMarina P., ArrietaAnna, Sroka-BartnickaMarina Yu., KorolevaAnna, StepienMarina, BorodachenkovaAnna, StrąkowskaMarina, Cabral PintoAnna, SurdackaMarina, D’AntimoAnna, SzcześniakMarina, DelucchiAnna, SzwajcaMarina, FialloAnna, SzymczykMarina, KnyazevaAnna, TampieriMarina, MariniAnna, TasolamprouMarina, Martínez-CarmonaAnna, TrauthMarina, MassaroAnna, ValenteMarina, MynbaevaAnna, VilàMarina, PozzoliniAnna, WoloszykMarina, RuthsAnna, WołowiczMarina, RynkovskayaAnna, ZykovaMarina, SamodurovaAnnabel, FernandesMarina, SchwanAnna-Liisa, KuboMarina, SpankaAnna-Liisa, PeikolainenMarina, ValentukevicieneAnnalisa, BrunoMarina, VukojeAnnalisa, DalmoroMarinca Traian, FlorinAnnalisa, PaoloneMarinko, BarukčićAnnalisa, VolpeMarino, BrcicAnnamaria, ViscoMario Daniele, PiccioniAnnarita, StringaroMario Lucio, PuppioAnnav, DettlaffMário R., VasconcelosAnnav, FarquharMario, AparicioAnne, HofmeisterMario, BeinerAnne-Marie, KietzigMario, CacciaAnnett, Dorner-ReiselMario, CommodoAnnunziata, PalumboMario, D’AcuntoAnqi, ZhangMario, DioguardiAnssi, LaukkanenMario, GrassiAntanas, ŠapalasMario, LavellaAntaryami, MohantaMario, MiscuglioAnthony M., WaasMario, Perez-SayansAnthony N., PapathanassiouMário, PolidoAnthony Neal, WatkinsMario, ProsaAnthony S. T., ChiangMario, RaspantiAnthony, ClarkeMǎrioara, MoldovanAnthony, DichiaraMarion, SeierstenAnthony, FavaloroMarios C., PhocasAnthony, TavaresMarios E., KazasidisAnthony, ThuaultMarios, SophocleousAnthony, TorresMaris, KlavinsAntigoni, PanagiotopoulouMaris, SinkaAntoine, Durocher-JeanMarisa, NicolaiAntoine, LétoublonMarisol, FaraldosAntoinette M., ManiattyMarius, AleinikovasAntolino, GallegoMarius, AndruhAnton A., NaumovMarius, EnachescuAnton G., KutikhinMarius, MiricioiuAnton S., KonopatskyMarius, PrelipceanuAnton S., ShabuninMarius, PustanAnton V., NaumovMarius, TintelecanAnton Yu., NalivaikoMarius, TreiderisAnton, ChepurnenkoMarius-Andrei, OlariuAnton, KotovMariusz, BanaszkiewiczAnton, ManakhovMariusz, BoberAnton, PopovMariusz, DąbrowskiAnton, SmirnovMariusz, DejaAnton, TrníkMariusz, DudekAnton, ZubrikMariusz, GackowskiAntonella Caterina, BocciaMariusz, HasiakAntonella, CurulliMariusz, JaczewskiAntonella, D’AlessandroMariusz, JaśniokAntonella, GuzzonMariusz, KosobudzkiAntonella, Marino GammazzaMariusz, KostrzewskiAntonella, PiozziMariusz, KrólAntonella, RizzoMariusz, MagierAntonello, MaroccoMariusz, MamińskiAntonello, VicenzoMariusz, RadtkeAntoni, CladeraMariusz, WalczakAntonietta Lo, ConteMariusz, WalkowiakAntonino, La MagnaMariusz, WesołowskiAntonino, QuattrocchiMariya Aleksandrovna, VikulovaAntonino, ReccaMariya, AleksandrovaAntonino, SantoniMariya, Konsulova-BakalovaAntonino, ScandurraMariya, SpasovaAntonino, SpadaMarjan, MarinšekAntonio Berná, GalianoMarjan, NezafatiAntonio Di, BartolomeoMarjan, TusarAntonio Eduardo, PalomaresMarjana, NovičAntonio J., SalinasMarjetka, ConradiAntonio J., Sánchez EgeaMark James, JacksonAntonio José, Lozano GuerreroMark W., BeattyAntónio Jose, Paleo VieitoMark, ArnouldAntonio José, Tenza-AbrilMark, BegoniaAntonio Juan, Dos Santos-GarcíaMark, GilbertAntonio Lopez, UcedaMark, JacksonAntónio M., PereiraMark, PotterAntonio Mario, LocciMark, RainforthAntonio Norio, NakagaitoMark, RinnerthalerAntónio Paulo, Monteiro BaptistaMark, SidebottomAntonio, ApicellaMark, ŽicAntonio, BoccaccioMarkku, LeskeläAntonio, CaggianoMarko, BartolacAntonio, CallejasMarko, KatinićAntonio, CapezzaMarko, PavlovicAntonio, Cid-SamamedMarko, PetričAntonio, ConcilioMarkos, PetousisAntonio, CoppolaMarkus, GahleitnerAntonio, D’AndreaMarkus, GalleiAntonio, De CrisciMarkus, HärtelAntonio, Di BartolomeoMarkus, HofAntonio, Di MartinoMarkus, KnoblochAntonio, Díaz-ÁlvarezMarkus, PristovsekAntonio, Díaz-ParralejoMarkus, RatzkeAntonio, Doménech-CarbóMarkus, StommelAntonio, FernándezMarkus, VargaAntonio, FerraroMarta Molina, HuelvaAntonio, FronteraMarta Peña, FernándezAntonio, GalganoMarta Roig, FloresAntonio, GloriaMarta Šavor, NovakAntonio, GrandeMarta, Cabeza SimoAntonio, GrecoMarta, ChecchiAntonio, LaezzaMarta, ChylińskaAntonio, LamuraMarta, Del ZoppoAntonio, LasantaMarta, Ferreiro-GonzálezAntonio, OrlandiMarta, Fiedot-TobołaAntonio, PapagniMarta, GmurekAntonio, Pedrero GonzálezMarta, GrochowiczAntonio, PereiraMarta, HarničárováAntónio, Pereira GonçalvesMarta, KałużaAntonio, PetošićMarta, KepinskaAntonio, PiccininniMarta, KopaczyńskaAntonio, PizziMarta, Kosior-KazberukAntonio, RibeiroMarta, MadaghieleAntonio, RiveiroMarta, MazurAntonio, SandoliMarta, MikuśkiewiczAntonio, SansonettiMarta, MuñozAntonio, ScaranoMarta, PalaciosAntonio, Torres MarquesMarta, PutrinšAntonio, VinciMarta, Roig-FloresAntonio, ZuorroMarta, SkafAntonios N., PapadopoulosMarta, ŚlęzakAntonios, BanosMarta, StucchiAntonios, KelarakisMarta, SuárezAntoniya, TonchevaMarta, TunesiAntony, AnanthMarta, WasilewskaAnubhab, MukherjeeMarta, Woźniak-BudychAnuj, KumarMarta, Ziegler-BorowskaAnup, BasakMartin A., HubbeAnup, GangopadhyayMartin B., ØstergaardAnúp, PandíțhMartin Ruthandi, MainaAnupam, RoyMartin, BaumgartenAnurag, SharmaMartin, BrehmAnushree, DasMartin, BystrianskyAnvesh, GaddamMartin, CadaAnya, VollprachtMartin, DankoAo, ZhouMartin, DresselAoife K., LucidMartin, FiskAomar, HadjadjMartin, FriákAphrodite, KtenaMartin, GerickeApostolos, KorlosMartin, GurkaAqiang, LinMartin, JassoAraliya, MoslehMartin, KapitánAram, BugaevMartin, KeppertArantzazu, Valdés GarcíaMartin, KolekArash, AhmadivandMartin, KollerArash, JenabMartin, KučerkaAravin Prince, PeriyasamyMartin, LedererArcady, DyskinMartin, LeitnerArezoo, ZareMartin, LutzAri, LehtonenMartin, MotolaAriane, Perez-GavilanMartin, NicolausAriful, HaqueMartin, OrendáčAris, GiannakasMartin, OschatzAristide, GiulianoMartin, PalouAristides, BakandritsosMartin, PetrunAristidis S., VeskoukisMartin, PipiskaAristotelis, SgourosMartin, RožánekArjun Prasad, TiwariMartin, SahulArkadeep, KumarMartin, StěničkaArkadii, ArinsteinMartin, TaylorArkadiusz, AdamczykMartin, TunnicliffeArkadiusz, DenisiewiczMartin, VašinaArkadiusz, DziedzicMartin, VögeleArkadiusz, JózefczakMartin, VyšvařilArkadiusz, MordakMartin, WeisArkadiusz, NędzarekMartin, WildingArkadiusz, TomczakMartina, CappellettiArkady, SerikovMartina, PetranikovaArkady, VoloshinMartínez García, CarmenArkaprabha, KonarMartyna, GrzegorzekArman, ShojaeiMartynas, LelisArmando, CaseiroMaruf Nur, HossainArmando, Fernandez-PrietoMary, HardieArmin, AbediniMaryam, HojatiArminda, AlmeidaMaryam, Mobed-MiremadiArmindo, Armindo MeloMarzanna, KsiazekArnab, DawnMarzena, KurpinskaArnab, Sen GuptaMarzena, LachowiczArnaldo, CasalottiMarzena, Mitoraj-KrólikowskaArnaud, DuchosalMarzena, PawlikArnaud, Le FebvrierMarzena, SutowskaArnaud, PerrotMarzio, GrassoArne, LindbråthenMasahiko, ItoArnold J., KellMasahiko, YamaguchiArnoldas, ŠneiderisMasahisa, FujinoArnoldo, LopezMasakazu, AtobeAroldo J., Romero-AnayaMasakazu, IchikawaArpad, MolnarMasaki, KakiageArpan, KunduMasami, OkamotoArtem, EreminMasanari, HiraharaArtem, MarchenkovMasanari, NagasakaArtur P., TerzykMasanori, KikuchiArtur R., ShugurovMasao, KuniokaArtur Władysław, GanczarskiMasao, YamagishiArtur, ChrobakMasashi, KatoArtur, CzupryńskiMasashi, OhashiArtur, IlukMasataka, KuboArtur, KasprzakMasato, TanakaArtur, NemśMasato, UedaArtur, NowoświatMasatoshi, SaitouArtur, PiekarczukMasatoshi, SakairiArtur, RydoszMasatoshi, TosakaArtur, VashurinMasaya, HigashiArtur, WolakMasayuki, OchiArtūras, KilikevičiusMashaalah, ZarejousheghaniArtūras, ŽalgaMasoud, HemmatianArturo, Sanchez-PerezMasoud, HosseinpoorAru, YanMasoud, Jahandar LashakiArun Prasanth, NagalingamMasoud, Nazarian-SamaniArun, ArjunanMasoud, ShekargoftarArun, JaganathanMasoume, DavoudiArun, KesavanMasrur, MahediArunabha RoyMassimiliano, AvalleArunas, JagminasMassimiliano, CavalliniArūnas, JarasMassimiliano, FerrucciArunas, RamanaviciusMassimo, CarossaArvaidas, GaldikasMassimo, DuranteArvind, GuptaMassimo, LazzariArvydas, PaleviciusMassimo, LorussoAsad, HanifMassimo, OttonelliAsal, KiazadehMassimo, PiottoAsanka Sajini, YapaMassimo, PolettoAsghar, Gholizadeh-VayghanMassimo, RovereAsheesh, LanbaMassimo, TessarottoAshim, DhakalMassimo, VivianiAshiqur X., RahmanMassoud, SofiAshish, GanvirMatei D., RaicopolAshit, RaoMatej, BabičAshkan, BozorgzadMatej, KomeljAshok Kumar, MeiyazhaganMatej, ParAshutosh, SharmaMatej, PašákAshutosh, ThakurMateja, KertAshutosh, TiwariMateja, KlunAshveen, NandMateus Garcia, RochaAsit Kumar, GainMateusz, BrelaAspasia, SarafianouMateusz, CzyzyckiAsta, TamulevičienėMateusz, FicekAsun, CanteraMateusz, IwańskiAsuncion, Garcia-EscorialMateusz, KowalskiAswini K., PradhanMateusz, KoziolAsya, Drenkova-TuhtanMateusz, KuklaAta, MeshkinzarMateusz, MrukiewiczAtanas, KaterskiMateusz, SchabikowskiAtanu, JanaMateusz, SikoraAthanasia, TolkouMatheus A., TunesAthanasios C., MitropoulosMathias, HerrmannAthanasios, GoulasMathias, LiewaldAthanasios, SalifoglouMathias, WoydtAthanasios, SkourasMathieu, DomenjoudAthina, AngelopoulouMathieu, TernerAtsufumi, HirohataMatija, BušićAtsushi, InoishiMatija, JezeršekAttila, BendeMatilda, ZemanováAttila, CsíkMatjaž, KristlAttila, DiószegiMatjaž, PavličAttila, GéczyMato, PericAtul D., SontakkeMatteo, BenedettiAtul, BansodeMatteo, BonomoAudrey, GbaguidiMatteo, GhidelliAuezhan, AmanovMatteo, GuidottiAugusto Cannone, FalchettoMatteo, MonaiAugusto Claudio, MarcelliMatteo, PanizzaAugusto, Cannone FalchettoMatteo, SambucciAugusto, OrlandiMatteo, ScaramuzzaAurea Immacolata, LumbauMatteo, SorrentiAurel, TabacaruMatteo, StrozziAurelia, Alonso-MedinaMatteo, TieccoAurelia, MegheaMatteo, TonezzerAurelia, VisaMatteo, ZanoccoAurelian, MarcuMatthew A., MarcusAurelian, PopescuMatthew Greig, CowanAurélie, TaguetMatthew L., WhitakerAurélien, BesnardMatthew P., SpellingsAurélien, DeniaudMatthew, AdamsAurélien, DoitrandMatthew, PanAurelio Hierro, RodriguezMatthew, SwensonAurelio, CabezaMatthew, WadeAurelio, García-ValenzuelaMatthias, AhlhelmAušra, MažeikienėMatthias, AlbiezAuxi, BarbudoMatthias, BickermannAvik, HalderMatthias, EckertAvinash, KadamMatthias, KapischkeAvneesh, KumarMatthias, KernAwais, IkramMatthias, KochAwetehagn, TuaumMatthias, KuntzAxel, GottschalkMatthias, PrellerAxel, PelsterMatthias, SchnabelrauchAy Ching, HeeMatthias, SchwotzerAyako, WashioMatthias, SteinbacherAyan, BhowmikMatthias, ZehnderAyar, PooyanMatthieu, RauchAydar, RakhmatullinMatthieu, WeberAydin, BerenjianMatti Il mari, PutkonenAyman, El-ZohairyMatti, KalliokoskiAymen, YanguiMatti, LindroosAyodele, OlofinjanaMatti, VirkkiAzadeh, SheidaeiMattia, BartoliAzam, NabizadehMattia, BiesuzAzarmidokht, Gholamipour-ShiraziMattia, PierpaoliAzhagiya Singam, Ettayapuram RamaprasadMattias, ThuvanderAzizur, RahmanMaurice H. T., LingAzzeddin, NagharMaurice, CollinsBabak, AshouriradMauricio, MoraisBabak, Shalchi-AmirkhizMaurizio, BellottoBach, TranMaurizio, D’AmarioBae, DowonMaurizio, MussoBaer, WolframMauro Fernandes, PereiraBagdat, TeltayevMauro Francesco, SgroiBailey, BubachMauro, CoduriBaiquan, LiuMauro, GiorcelliBalasingam, MuhunthanMauro, GiudiciBalazs, EndrodiMauro, MalvèBalázs, IllésMauro, RicottaBalázs, ImreMauro, SassuBalázs, MikóMavinakere Eshwaraiah, RaghunandanBalázs, NagyMaw Maw, TunBalazs, TothMaw Tien, LeeBandar, AlMangourMax, HeilandBang Yeon, LeeMax, HossfeldBangjing, LiMaxence, UrbaniBankole, OladapoMaxim G., KhomutovBaohua, ChangMaxim M., KorshunovBaomin, WangMaxim V., GorkunovBaoming, WangMaxim, BermeshevBaozeng, RenMaxim, GalkinBär, JürgenMaxim, MolokeevBarbara, BallarinMaxim, OzerovBarbara, Bobrowska-KorczakMaxim, PyatnovBarbara, CharmasMaxime, DucretBarbara, CichyMaximilian, AckermannBarbara, CosantiMaximilian, BesenhardBárbara, FerreiraMaya, Narayanan NairBarbara, FlorisMayakrishnan, PrabakaranBarbara, FranckeMayri Alessandra, Diaz De RienzoBarbara, GawdzikMaysam B., GorjiBarbara, GilMayzonee, LigarayBarbara, GoszczyńskaMazdak, TootkaboniBarbara, HissaMazen, Al-KheetanBarbara, KozubMaziar, MahdaviBarbara, KucharskaMd. Ahasan, HabibBarbara, ŁapińskaMd. Ashraf, HossainBarbara, MiroslawMd. Ashraful Islam Molla, MollaBarbara, NasiłowskaMd. Rashadul, IslamBarbara, PanunziMd. Shakhaoath, KhanBarbara, PatriziMd. Tanvir, HasanBarbara, PawelecMd., SalauddinBárbara, Pérez-KöhlerMeaurio, EmilianoBarbara, PietruszkaMeenashisundaram, Ganesh KumarBarbara, ReggianiMehdi, BenhassineBarbara, SacchiMehdi, GhazimoradiBarbara, Šetina BatičMehdi, HosseinzadehBarbara, SurowskaMehdi, MehraliBarbara, Szpikowska-SrokaMehdi, SalimiBarbara, Tchórzewska-CieślakMehdi, ShishehborBarbara, VercelliMehmet, YilmazBarbora, DoušováMehran, DadkhahBarbora, NečasováMehran, KianiniaBaris, KumruMehrnoush, KhavarianBarkoczy, PeterMehrshad, MehrpouyaBarry D., DunietzMeikang, HanBarry, AldwellMeisam, GoudarzyBart, De GeestMeiyu, ZhaoBart, FollinkMelanie, EckerBart, PijlsMelinda L., JueBart, WeberMelkie, TadesseBartlomiej, GraczykowskiMeng, LingBartłomiej, IglińskiMeng, ZhangBartłomiej, WysockiMengfang, LiBartmański, MichałMeng-Hsiu, TsaiBartolomeo, CoppolaMeng-Ting, TsaiBartosz, BondziorMercedes Sánchez, MorenoBartosz, GapińskiMercedes, Del Río MerinoBartosz, PiątekMercier, FredericBartosz, PowałkaMert, CelikinBasanth Babu, EedaraMert, VuralBasilio, PueoMert, Yucel YardimciBastian, WessingMesut, YurukcuBastos Arrieta, JulioMi G., ChorzepaBattulga, MunkhbatMi, TianBeata, DudziecMia Mia, ThiBeata, HouškováMiao-lei, ZhouBeata, JaworskaMiće, JakićBeata, KaczmarekMichael A., BeckettBeata, Kalska-SzostkoMichael A., TrakselisBeata, KolesińkaMichael C. H., KargBeata, KurcMichael Cai, WangBeata, Łaźniewska-PiekarczykMichael D., CropperBeata, LemliMichael J. W., JohnstonBeata, Leszczyńska-MadejMichael P., MarderBeata, PodkoscielnaMichael P., MarkeyBeata, ŚmielakMichael R., KoblischkaBeata, SmyrakMichael R., MucaloBeata, StaniszMichael T., KoltzBeata, ZielinskaMichael, AnenburgBeata, ZimaMichael, ArkasBeatrice, BellettiMichael, ArulBeatrix, PéterMichael, AuingerBeatriz, Achiaga MenorMichael, BuchmeiserBeatriz, GonzálezMichael, CampbellBeatriz, Salinas RodríguezMichael, ChaeBeelee, ChuaMichael, CiesielskiBehnam, Jahangiri KoohbananiMichael, CordinBehnam, Kheyraddini MousaviMichael, El KadiBehnoush, GolchinfarMichael, FörsthBehzad V., FarahaniMichael, FrankBehzad, EsmaeliMichael, HadjiargyrouBehzad, FotovvatiMichael, KarnahlBejinariu, CosticaMichael, KassnerBekim, BerishaMichael, KlontzasBekir, BedizMichael, LeitnerBéla, IvánMichael, LorenzBelén, Begines RuizMichael, MarxBelén, Díaz FernándezMichael, ModigellBen, AlcockMichael, MorlockBen, AllardyceMichael, MückBen, BeakeMichael, MüllerBen, CerjanMichael, MunzBenedetto, NastasiMichael, NolanBenedicte, VertruyenMichael, OjovanBenjamin, BergmannMichael, OrtnerBenjamin, EftinkMichael, PetrouBenjamin, HertweckMichael, RethmeierBenjamin, HoganMichael, SeibtBenmansour, SamiaMichael, ToffoloBenoit, HamelinMichaela, GkantouBenoit, HilloulinMichaela, ŠlapákováBenoit, MalardMichail, KlontzasBenoit, MichotMichał J., WiniarskiBeomjoo, YangMichal, AckermannBerit, Zeller-PlumhoffMichał, BartmańskiBernadeta, DoboszMichał, BöhmBernard, GeffroyMichal, CichomskiBernardeta, DębskaMichał, DoroszkoBernardette, Soust-VerdaguerMichał, DrzazgaBernardo Gonçalves, AlmeidaMichal, HlobilBernardo, ZuccarelloMichal, JamborBernardus, RoozenMichal, KulkaBernd, BreidensteinMichal, ŁachBernd, KleimtMichal, LahavBernd, KuhfussMichał, LandowskiBernd, SchöllhornMichał, MoritzBernhard J., HoendersMichał, MusiałBernhard V. K. J., SchmidtMichał, NowickiBernhard, DörlingMichal, PetruBernhard, MinglerMichał, PuchalskiBernhard, SchmidtMichał, RachwalskiBernhard, SchulzMichal, RymsBernhard, SchusterMichal, ŠajgalíkBernhardt, Saini-EidukatMichał, SarnowskiBertha Maria Batista, Dos SantosMichal, SejnohaBetul Aldemir, DikiciMichal, SobaszekBetül, KüçüközMichał, SzydłowskiBhabani Sankar, SwainMichal, UrbánekBharat, GwalaniMichal, WieczorowskiBhaskar, DudemMichał, WojasińskiBhishma R., SedaiMichel, SassiBhogeswararao, SeemalaMichela, BorghettiBhupendra, SharmaMichela, BosettiBhuvanesh, SrinivasanMichela, RiccaBianca, GalateanuMichela, SimonciniBibiana María, Fernández-PérezMichele, BettiBice, ContiMichele, CalìBilen Emek, AbaliMichele, CasielloBilly James, MurdochMichele, CassettaBin, FeiMichele, D’AmatoBin, WangMichele, DallagoBin, XuMichele, De SantisBin, YangMichele, FedelBin, YaoMichele, FerraiuoloBin, ZhangMichele, GuidaBinbin, HeMichele, MastroianniBing, ChenMichele, MattioliBing, HanMichele, MontanariBing, WangMichele, PerrellaBing, YeMichele, TepedinoBing, YuanMichelle, PantoyaBingfeng, WangMichelle, SalviaBinghao, WangMichihide, KitamuraBingran, YuMichihiko, NagumoBingyan, JiangMicka, BahBinsheng, ZhangMieczysław, PancielejkoBipasha, BoseMieczyslaw, ScendoBipin, GaihreMieczysław, SłowikBiplab, PaulMieczysław, SzataBiranchi, PandaMieszko, WieckiewiczBishnu, DahalMiguel A., CamblorBishnu, GautamMiguel A., Gómez-PoloBishweshwar, PantMiguel Ángel, Caminero TorijaBiswadeep, SahaMiguel Angel, CentenoBiswanath, DuttaMiguel Ángel, Delgado CantoBjörn, LüssemMiguél Ángel, GutierrezBlake, HildrethMiguel Ángel, Laguna-BerceroBlas, CanteroMiguel Angel, OrtegaBlaž, LikozarMiguel Ángel, SanjuánBłażej, KudłakMiguel Angel, Selles CantoBłażej, ScheibeMiguel G., AcedosBłażej, TomiczekMiguel, AlmeidaBo, CuiMiguel, Castro-ColinBo, MaoMiguel, Gisbert-GarzaránBob, HowellMiguel, Gomez-HerasBobby, MathanMiguel, JulveBocong, ZhengMiguel, LleraBodor, MariusMiguel, LorenzoBogdan Cătălin, ȘerbanMiguel, LozanoBogdan M., DiaconuMiguel, MoralesBogdan, AntoszewskiMiguel, Muñiz-CalventeBogdan, ChiritaMiguel, NepomucenoBogdan, CulicMiguel-Angel, Perea-MorenoBogdan, MunteanuMiha, HumarBogdan, PiwakowskiMihael, BrunčkoBogdan, RutkowskiMihaela, AlbuBogdan, SamojedenMihaela, BacalumBogdan, SłodkiMihaela, BadeaBogdan, SmykMihaela, OleksikBogdan, SzybińskiMihaela, Radu (Balas)Bogdan, WarcholińskiMihaela, TertisBogdan, ZygmuntMihaela, UlmeanuBogumiła, KumanekMihaela, VlaseaBogumiła, KuźnickaMihaela-Violeta, GhicaBogusław, BiedaMihai Alin, PopBoguslawa, Adamczyk-CieslakMihai V., PutzBohayra, MortazaviMihai, BrebuBohumír, StrnadelMihai, MusteataBojan, MilovanovicMihai, NitulescuBojan, PodgornikMihai, OaneBojan, StarmanMihaiela, IliescuBojana, VoncinaMihail, SecuBojidarka, IvanovaMihai-Tiberiu, LatesBolesław, SzadkowskiMihalis, FakisBongju, KimMihály, PurgelBong-Kee, LeeMija H., HublerBonnie R., AntounMika, SalmiBoon Peng, ChangMikael J., TurunenBoonya, CharnnokMikael, JensenBoren, LinMikael, PerrutBoris A., KhasainovMikael, Vejdemo-JohanssonBoris A., KulnitskiyMike J., JenkinsBoris A., UmanskiiMike, BambachBoris B., StraumalMike, BarbeckBoris, DyatkinMike, BuckleyBoris, GnesinMike, HurleyBoris, LakardMikhael, BechelanyBoris, MahltigMikhail A., Il’yushinBoris, MarkovskyMikhaïl A., LebyodkinBoris, TenchovMikhail F., BudykaBoris, WilthanMikhail M., MaslovBorja, Arroyo MartínezMikhail Mikhailovich, SkripalenkoBorja, EriceMikhail V., GolubBorja, IzquierdoMikhail V., VenerBorja, VaronaMikhail Yegorovich, SinelnikovBorowiecka-Jamrozek, JoannaMikhail, BannikovBor-Shiunn, LeeMikhail, EreminBo-Sung, ShinMikhail, SevostyanovBoto E. F., RenatoMikhail, ShamoninBouazizi, NabilMikhail, SinelnikovBoussad, AbbesMikhail, SlobodyanBowei, DongMikhail, StarostenkovBoyang, HuangMikhail, VaginBozena, PietrzykMikhail, ZheludkevichBożena, Szczucka-LasotaMikio, ItoBożena, TyliszczakMikko, KanervaBrahmananda, PramanikMiklós, BakBrain, ViaMiklós, SerényiBrandis, KellerMikołaj, LewandowskiBranko, ŠavijaMikołaj, MiśkiewiczBranko, TrajkovskiMikolaj, PochylskiBraulio, García-CámaraMikolaj, SchmidtBre-Anne, SainsburyMilad, GhayoorBrian A., OmondiMilad, Hamidi NasabBrian A., RosenMilad, Pourbaghi MasoulehBrian E., GrottkauMilad, SaeedifarBrian G., FrederickMilada, GajtanskaBrian J., JaquesMilan, BrandtBrian J., WisnerMilan, ČervenkaBrian W., DarvellMilan, KadnárBrian, LattimerMilan, KlicperaBrian, RodriguezMilan, MarônekBrian, RoweMilan, OnčákBrienne N., SeinerMilan, SedlarBrigit, MittelmanMilan, TerčeljBrigitte, GrosgogeatMilan, UhríčikBrina J., BlinzlerMilan, ŽmindákBrisid, IsufiMilena G., GeorgievaBruce L., TaiMilena, KušnerováBruna, SinjariMiloš, JermanBruno, BriseghellaMiloš, PánekBruno, BuchmayrMin Seok, MoonBruno, ChrcanovicMin, ParkBruno, LuchiniMin, SongBryan, HunterMin, TuBryan, WongMin, WangBugga V., RatnakumarMin, YueBurak, GerisliogluMinas, StylianakisBurcu, GumuscuMindaugas, GedvilasBurkhard, PlinkeMing Jen, TanBurkhard, SchulzMing, JinBusisiwe Chikomborero Ncube, MakoreMing, LuByeong Wan, AnMing, TangByoung Suhk, KimMingbo, PuByoung, Hooi ChoMing-Cheng, ShihByoungjin, SoMing-Chung, WuBystrik, DolnikMing-His, ChiangByung Hoon, KimMinghua, LiuByung Jae, LeeMinghui, HongByung Jun, JungMing-Jer, JengByung-Cheol, KimMingqiang, LiByung-jae, KangMingqing, WangByungjoon, LeeMingrui, ZhaoByungmin, AhnMing-Shien, YenByungseok, YooMingshun, JiangByung-Wook, ParkMingtu, JiaCaballero, ÁlvaroMingwen, BaiCagan, DiyarogluMingxiao, YeCaiwang, TanMingxing, LiCalin, VaidaMing-You, ShieCamelia, CerbuMing-Yu, LiCamelia, NegrutiuMingyue, LinCamil, LanceaMingzhe, ZhangCamilla, GallettiMingzhong, LiCamilla, KärnfeltMingzhou, ChenCamillo, SartorioMinh Chien, TrinhCamilo Andres, FrancoMinh Quang, PhamCamino Fernández, RodríguezMinh, LuongCan, ChenMin-Ho, HongCan, KoralMinhui, ChenCandelas, PaniaguaMinju, KangCândida, MalçaMin-ju, SongCanio, NoceMinkook, KimCao, WangMinliang, LaiCarina, CruchoMinmin, ZhuCarl James, DebonoMinoru, KuniedaCarl, SchaschkeMintaek, YooCarla Maria, CostaMinwoo, ChangCarla, BazzicalupiMin-wook, OhCarla, GasbarriMinxiang, ZengCarla, GentileMioara, BercuCarla, PalumboMiodrag, MicicCarl-Eric, WilenMiquel A., Chamorro-TrenadoCarlo Alberto, BiffiMir Jalil, RazaviCarlo, BruniMira, NaftalyCarlo, CastellanoMira, ParkCarlo, FornainiMiran, MerharCarlo, PastoreMiran, MozetičCarlo, PaternosterMiran, UlbinCarlo, SantiniMiranda, FateriCarlo, SantulliMircea Cristian, DudescuCarlos David, Grande TovarMircea Dorin, VasilescuCarlos Fernando, MourãoMircea, ChiparaCarlos G., JuanMircea, DragomanCarlos J., Durán-ValleMircea, NicoaraCarlos J., Gómez GarcíaMircea, TampaCarlos Miguel, CostaMirco, PeronCarlos Miguel, MartoMirei, ChibaCarlos, AndradeMirela, RožićCarlos, CaroMirela-Fernanda, ZaltariovCarlos, DrummondMirijam, VrabecCarlos, EscuderoMirko, NitschkeCarlos, Garcia-GonzalesMirna, Habuda-StanicCarlos, López-ColinaMiroslav, CernyCarlos, MarquezMiroslav, CieslarCarlos, Navarro PintadoMiroslav, GombárCarlos, PauloMiroslav, HalilovičCarlos, Rivera-GómezMiroslav, KarlíkCarlos, Rizo-MaestreMiroslav, ManasCarlos, Romero-MuñizMiroslav, MrlíkCarlos, SalasMiroslav, NěmecCarlos, Sotelo-VazquezMiroslav, NeslušanCarlos, ThomasMiroslav, PohankaCarlotta, GirominiMiroslav, SahulCarlotta, PontremoliMiroslav, SloufCarlotti, MarcoMiroslav, TomášCarme, Martínez-DomingoMiroslav, VarinyCarmel B., BreslinMiroslav, ZecevicCarmela, GurauMiroslava, Dušková-SmrčkováCarmelo Lo, VecchioMirosław S., BonekCarmelo, CaggegiMiroslaw, SzalaCarmelo, MajoranaMiroslaw, SzybowiczCarmelo, ScuroMiroslawa, PawytaCarmen, AndradeMiroslawa, ProchonCarmen, CiotoneaMiruna, StanCarmen, García-RosalesMislav, StepinacCarmen, LlenaMithil, MazumderCarmen, PerezMithilesh, YadavCarmen, RacanelMitja, PetričCarmen, Rodríguez LiñánMitsuaki, MatsuokaCarmine, AutieriMitsuharu, TodaiCarmine, CapacchioneMitsuhiro, ShigeishiCarmine, D’AgostinoMitsuo, NotomiCarmine, LimaMizanur, RahmanCarmine, PappallettereMizue, MizoshiriCarol, BarryMizuki, TenjimbayashiCarola Esposito, CorcioneModestas, KligysCarolina, Piña RamirezMogalahalli V., ReddyCarolina, Ripolles-AvilaMoh, AmerCarolyn M., PrimusMohamad Hassan, AminCarson J., BrunsMohamed A., ShenashenCarter, HamiltonMohamed M., ChehimiCaspar, ClarkMohamed Nawfal, GhazzalCastaldo, RacheleMohamed, AbdelrahmanCastejón Herrer, LuisMohamed, BakarCastillo-Rivera, SalvadorMohamed, ChowdhuryCastorina Silva, VieiraMohamed, El MehtediCătălin CroitoruMohamed, El-ZariaCatalin, AmzaMohamed, F. AttiaCătălin, AraniciuMohamed, GuessasmaCătălin, MarinMohamed, HamdaouiCatalin, PopaMohamed, IbrahimCatalin, PruncuMohamed, ShalabyCatalin, ZahariaMohammad Ali, Mohtadi BonabCatalina Mihaela, GrădinaruMohammad Amin, Hariri-ArdebiliCatalina, MaierMohammad Boshir, AhmedCatalina, Natalia Cheaburu, YilmazMohammad J., MirzaaliCatalina-Alice, BrandusMohammad Mahbub, RabbaniCatalin-Daniel, ConstantinescuMohammad Mehdi, RashidiCatarina Pereira, NobreMohammad Mohsen, SarafrazCatarina, ReisMohammad Nurul, IslamCatarina, VidalMohammad R., KabirCaterina, LiciniMohammad Reza, JandaghiCaterina, PipinoMohammad Reza, KhosravaniCaterina, SoldanoMohammad Reza, SafaeiCatherine, KellyMohammad Shafiqur, RahmanCatherine, TuleuMohammad Shahrear, KabirCattin, LindaMohammad Yaghoub Abdollahzadeh, JamalabadiCécile, DilibertoMohammad, Akbari GarakaniCecile, LangladeMohammad, BazzazCecilia, SuraceMohammad, Faisal HaiderČedomir, OblakMohammad, IslamCédric, DelattreMohammad, KarimiCédric, JaoulMohammad, MahdaviCédric, PardanaudMohammad, MalekanCédric, Van GoethemMohammad, MastaliCedric, VuyeMohammad, MofidfarCélia F., RodriguesMohammad, NadimiCélio Gabriel Figueiredo, PinaMohammad, RafieeCem, ÖrnekMohammad, SaffariCésar Augusto Correia, De SequeiraMohammad, ShariareCesar, JaureguiMohammadReza, SeyediCésar, Martín-GómezMohammadreza, ShokouhimehrCésar, MedinaMohammed A., FaruqiCesare, CamettiMohammed Abdul, HannanCesare, Oliviero RossiMohammed El Amine, SlamaCesare, SignoriniMohammed Nadeem, BijleCezary, SenderowskiMohammed, IsmaelChadrasekhar, LokaMohammed, KoubitiChaenyung, ChaMohammed, MezianiChaker, FaresMohan, EdirisingheChamila, GunasekaraMohan, SakarChan Hyeong, ParkMohankandhasamy, RamasamyChandani, SenMohannad Z., NaserChang Jiang, WangMohannad, MayyasChang, LiuMohanraj, VinothkannanChang, PengMohit, MehtaChang, QiMohsen, AzimiChang-Cheng, ChenMohsen, MoslehChanggu, LeeMohtada, SadrzadehChangheui, JangMoinul, MahdiChanghong, CaoMoisés Luzia, PintoChanghyun, RohMoises, BuenoChangjoo, ParkMojca, JazbinsekChanglu, ZhouMojca, OtoničarChang-Whan, LeeMojmír, ŠobChangxiang, WangMona, EskandariChangyong, YimMonajati, HosseinChanho, PakMonday, OkoronkwoChan-Jung, KimMoneeb, GenedyChao, GaoMonia, SperaChao, LiuMonica Cartelle, GestalChao, ZhangMónica, CarreraChaofang, DongMonica, DistasoChaofeng, WangMonica, FlorescuChaolong, TangMonica, FuensantaChaoqun, ZhangMonica, NardiChao-Sung, LinMonica, OliveiraChao-Wei, TangMonica, SantamariaChara, SimitziMónica, SilvaCharles, DismukesMonica, SimionCharles, FortmannMonica, TonelliCharles, FrazierMonica, YamautiCharles, KwanMonika, BakierskaCharles, ManièreMonika, FurkoChat-Tim, TamMonika, Gołda-CępaChe Nan, KuoMonika, GwoździkChe-Chen, ChangMonika, HertenChee Kwan, GanMonika, JenkoChen, FangMonika, KubzovaChen, ShiMonika, KuliszChen, WangMonika, Lukomska-SzymanskaChen-Chi, TsaiMonika, MadejCheng, ChenMónika, MolnárCheng, JiangMonika, OstapiukCheng, LianMonika, RatajczakChengbin, ZhangMonika, SkowrońskaChengcheng, TaoMonika, Śmiga-MatuszowiczCheng-Chung, LeeMonika, Wałęsa-ChorabChengde, GaoMonika, WawrzkiewiczCheng-Fu, YangMonique, Calvo-DahlborgCheng-Hsun, HsuMonirosadat, SadatiCheng-Hsun-Tony, ChangMonoj, GhoshChengliang, HuMonssef, Drissi-HabtiChengmin, JiangMontassar Billeh, BouzouraaCheng-Ming, TangMontserrat, ColillaChengqi, SunMontserrat, TobajasChengsong, CuiMoon-Jo, KimChenguang, FuMorena, NocchettiChengying, BaiMorena, PetriniChenhui, PengMoreno, ConcezziChen-I., YangMoreno, MarcelliniChennakesava, KadapaMorshed, KhandakerChenning, ZhangMorteza, AfsariChenqi, TaoMorteza, ArameshChensheng, WuMorteza, Hasanzadeh KafshgariChenshuang, LiMorteza, RasoulianboroujeniChen-Xuan, WeiMorwenna, SpearChenyang, LuMosab, KaseemChen-Yen, TsaiMostafa, NabawyCheryl Suwen, LawMostafa, Safdari ShadlooChetan, TambeMostafa, SeifanChia-Che, HoMostafa, YakoutChia-Chen, ChangMostafa, YazdimamaghaniChiachung, ChenMostafa, YourdkhaniChia-Hung, HungMostaqur, RahmanChia-Hung, LeeMotoki, UedaChiara Maria, MottaMotonori, WatanabeChiara, AttanasioMounir, ChaouchChiara, BattocchioMoussa, ZaarourChiara, BedonMuamer, KadicChiara, BrulloMuc, AleksanderChiara, ElmiMuchao, QuChiara, FerraraMudinepalli, Venkata RamanaChiara, GiosuèMuhamed, SućeskaChiara, MaccatoMuhammad Atiq, Ur RehmanChiara, MazzoniMuhammad Danang, BirowosutoChiara, MusumeciMuhammad Shahzad, KamalChiara, PiraniMuhammad Tayyab, NomanChiara, PlatellaMuhammad, AamirChiara, PortesiMuhammad, AteeqChiara, RiccaMuhammad, HossainChiashain, ChuangMuhammad, KashifChia-Wen, WuMuhammad, KhattabChi-Ching, KuoMuhammad, NabeelChiemela Victor, AmaechiMuhammad, QasimChien-hong, LinMuhammad, TahirChien-Kuo, HsiehMuhammad, UsmanChien-Wen, HsiehMuhammed Said, BoybayChien-Yie, TsayMuhibUr, RahmanChih Chiang, HongMukesh Kumar, PandeyChih-Cheng, YangMulugeta, WayuChih-Chia, ChengMumin Enis, LeblebiciChih-Chieh, HsuMunehiro, KimuraChih-Chien, ChuMunish Kumar, GuptaChih-Chun, HsiehMunkhbayar, BatmunkhChih-Feng, HuangMurali Krishna, GhatkesarChih-Hao, LeeMurali Mohan, CheepuChih-Hsuan, ChenMuruganandan, ShanmugamChihiro, SuzukiMuttin, FrédéricChih-Liang, WangMykhaylo, PotopnykChih-Wei, ChiuMykola, AbramchukChikashi, SatoMykolas, DaugevičiusChin Lung, ChiangMyroslav, SprynskyyChin-Chao, ChenMyrtil L., KahnChin-Chung, WeiMyung Hwan, ParkChing-Chich, LeuMyung Hyun, KimChing-Hsin, WangMyung Jun, KimChiou-Hwa, YuhMyung-eun, SukChiranjeevi, KorupalliMyung-Ki, KimChiranjivi, BhattaraiMyungkoo, KangChitiphon, ChuaichamNa, OkpinChiung-Wu, SuNabanita, SahaChi-Vinh, NgoNabeen K., ShresthaChiwai, KanNabil, DaghboujChobin, MakabeNabil, NassifChong Yang, ChuahNacú B., HernándezChongchong, QiNader, ShehataChonggui, LiNadezda, LangovaChongyang, LiuNadezda, StevulovaChoong-Un, KimNadezhda Alexandrovna, KochetovaChoon-man, LeeNadezhda, BokachChris G., KarayannisNadezhda, DudovaChris J., BarnettNadia, BarberoChris S., HawesNadia, Elghobashi-MeinhardtChris Valentin, NielsenNadia, JesselChris, FancherNadica, Maltar-StrmečkiChris, FellowsNadir, BettacheChris, SteffiNadka Tzankova, DintchevaChrister, JohanssonNag Jung, ChoiChristian J., StorkNagamalleswara Rao, AlluriChristian L., LaboisseNagendra Kumar, KaushikChristian M., JulienNahendra, DahotreChristian Mark, PelicanoNahum, TravitzkyChristian Oen, PaulsenNaito, HidekiChristian Wolfgang KarlNaiyang, MaChristian, AgatemorNak Cheon, JeongChristian, BrosseauNakata, TaikiChristian, HiepenNam Kon, LeeChristian, KirschneckNamboodiri, PradeepChristian, KloseNamchul, ChoChristian, KrambergerNamita, ShresthaChristian, KuklaNan, GaoChristian, MertensNan, GuiChristian, MotzNan, YuChristian, PariggerNantakan, WongkasemChristian, SchmitzNaoki, ShimosakoChristian, WillbergNaoki, TarutaniChristian, WirajaNaoki, WakiyaChristian-Alexander, BungeNaotaka, NittaChristiane, HerrmannNaoya, TadaChristiane, JungNapat, VajraguptaChristiane, SchulzNarayanasamy Sabari, ArulChristiansen, EmilNarayanaswami, RanganathanChristin, DavidNarcís Pous, RodriguezChristina, BirkelNarcis, BarsanChristina, KaravasiliNarcís, MestresChristina, PlatiNarcís, Pous RodríguezChristina, TangNarcisa, VrinceanuChristina, WüstefeldNarendar, DudhipalaChristine, BorchersNariman, EnikeevChristoph, HartlNarmina O., BalayevaChristoph, HartmannNaseem, AbbasChristoph, RameshanNaser, AliChristoph, StotterNasim Nsooudi, NsooudiChristoph, StrangfeldNatale, PerchiazziChristophe, BressotNatali Murri, AnnalisaChristophe, DrozNatalia Ivanovna, KozhukhovaChristophe, MartinNatalia, KamaninaChristophe, ValléeNatalia, KarpukhinaChristopher Adam, SenalikNatalia, MartynenkoChristopher J., WinfreeNatalia, MenshutinaChristopher J., WohlNatalia, PushilinaChristopher Neil, Hulme-SmithNatalia, SizochenkoChristopher P., KoharNatália, TomašovičováChristopher, BrownNatalie Williams, PortalChristos G., PapakonstantinouNatalija, VelićChristos N., PanagopoulosNataliya E., NovikovaChristos, AthanasiouNataliya, ChistyakovaChristos, MichailNataliya, MaksimchukChristos-Edward, AthanasiouNataliya, SakharovaChrysanthos, MaraveasNataliya, SaninaChrysoula, LitinaNataliya, ShaburovaChrystelle, SalamehNatalja, FjodorovaChua, Chee KaiNataly, SiqueiraChuan, LiNatalya, ShaburovaChuang, FengNataša, NáprstkováChuang, WenNathalie, KölblChuanlong, WangNathalie, SauerChuanrui, ChenNathalie, Siredey-SchwallerChuanxiong, NieNathan, JacksonChuen-Lin, TienNathan, LazarusChul Kyu, JinNathanaelle, SchneiderChul-Hee, LeeNavanietha Krishnaraj, RathinamChulhun, ParkNavaratnarajah, KuganathanChun-Chi, ChenNaveen, PrakashChunfu, XinNavid, HasheminejadChung Chen, TsaoNavid, RanjbarChung-Chiun, LiuNavin, KumarChung-Hwan, ChenNawar, KadiChung-Sun, LimNayomi, PlazaChung-Wei, YangNazar, FaridChun-Hu, ChengNazely, DibanChun-Huei, TsauNazmul, HudaChunju, GuNdue, KanariChunlei, YangNeamat Hassan, AbubakrChun-Liang, YehNeamul Hayet, KhansurChunping, DaiNechita, PetronelaChun-Rong, LinNectarios, VidakisChunsheng, WuNeeraj, BokdeChunwei, ZhangNeha, KaushikChun-Ying, HuangNeil D., MathurChuyen Van, PhamNeil, WaddellChuying, MaNeli, KosevaCiesielski, ArkadiuszNelson A. M., PereiraCimpoesu, NicanorNenad, TomasicCinthia Antunes, CorreaNengxiu, DengCinzia, CaliendoNeno, TorićCinzia, MasperoNeophytos, NeophytouCiprian, IacobNerija, ZurauskieneClaas, FischerNeringa, DirgėlienėClaire, AntoineNestor F., AguirreClara, CelauroNestor Washington Solís, PinargoteClara, PiccirilloNeven, HadžićClaro I., Sainz-DiazNeven, UkrainczykClaude, LeyderNgee Sing, ChongCláudia C. L., PereiraNghia P., TruongClaudia Maria, SimonescuNgoc Diep, LaiClaudia, BizzarriNgoc San, HaClaudia, FotiNguyen Duc, ChinhClaudia, GirjobNguyen Huu, TrungClaudia, NeunaberNguyen Quoc Khanh, LeClaudia, PelosiNguyen, Tam Nguyen TruongClaudia, PigliacelliNhat Truong, NguyenClaudia, TomasiniNhu, NguyenClaudiane, Ouellet-PlamondonNhung H. A., NguyenClaudine, WulfmanNia, RichardsClaudio, CeconeNiall, TaitClaudio, Di FrattaNicanor, CimpoesuClaudio, EvangelistiNiccolò, PampuroClaudio, FeroneNichola J., ColemanClaudio, FlorisNicholas A., BrakeClaudio, GennariNicholas A., PikeClaudio, LarosaNicholas F., MatererClaudio, LeoneNicholas P., StadieClaudio, PettinariNicholas, BraithwaiteClaudio, PoggioNicholas, DimakisClaudio, SantiNicholas, FantuzziClaudio, TestaniNicholas, FletcherClaudiu T., FleacaNicholas, HoltgreweClaudiu, AciuNicholas, PingitoreClaudiu, MaziluNicholas, SarlisClaus, MosekeNicholas, TeoClaus, RebholzNick Guan Pin, ChewClémence, PetitNick, SilikasClemens F., SchaberNick, VordosClement, CabanetosNicklas, AnttuClément, LacosteNickolai I., KlyuiClio, AzinaNickolay Yu., SdobnyakovCodruta, SarosiNico Alexander, GaidaCOL Chi, NguyenNicoara, MirceaColin, EslerNicola A., MorleyConal E., MurrayNicola H., GreenConcepció, SeguíNicola, BaldoConcetta, RispoliNicola, CalisiConrad, RizalNicola, CefisConstantin Mihai, LucaciuNicola, Contessi NegriniConstantin, ApetreiNicola, ContuzziConstantin, ChaliorisNicola, FabrisConstantin, HochNicola, LisiConstantina, PapatriantafyllopoulouNicola, MacchioniConstantino, TsallisNicola, MolinariConstantinos S., PsomopoulosNicola, MontinaroConstantinos, SimseridesNicola, TestoniCora, BulmauNicola, TrivellinCord M., BrundageNicolae, CiontCordula, VogelNicolae, UngureanuCorina J., BîrleanuNicolaie, PavelCorina Marilena, CristacheNicolas, CandauCornel, SamoilaNicolas, JacquelCorneliu Ioan, OpreaNicolas, LebonCorneliu, CojocaruNicolas, VanthuyneCorneliu, MunteanuNicole, JaffrezicCorrado, PiconiNicole, LounsburyCorson L., CramerNicoleta, PleşuCosmin Alexandru, DaescuNicoleta, PredaCosmin, CodreanNicoleta, RaduCosmin, CosmaNicoletta, DitarantoCosmin, CotetNien-Tsu, HuangCosmin, RomanitanNigam, MishraCostanzo, BelliniNigel T., LucasCostas A., AnagnostopoulosNiina, DulovaCostas A., CharitidisNikhil, AdmalCraig Denver, WilliamsNikhil, TiwaleCraig L., HillNikita A., KazarinovCraig, PrzybylaNikita, StepanovCristian I., ContescuNikita, YurchenkoCristian, DeacNiklas, SommerCristian, PetcuNikola, BregovićCristian, Rodríguez-TinocoNikola, GrgićCristian, ScheauNikola, KoutnáCristiana, RadulescuNikola, SakačCristiano, FragassaNikola, SaulacicCristina Manuela, DrăgoiNikolai, BoshkovCristina Martín, DoñateNikolai, TsvetkovCristina Mihaela, CampianNikolai, UvarovCristina Moreno, DíazNikolai, VatinCristina Sofia Dos Santos, NevesNikolaj, MoleCristina, AchimNikolaj, VišniakovCristina, ArgizNikolaos (Nikos), CheimariosCristina, ArtiniNikolaos A., ChrysanthakopoulosCristina, BignardiNikolaos C., KokkinosCristina, BirisNikolaos E., KarkalosCristina, BusuiocNikolaos G., SemaltianosCristina, CazanNikolaos, BalisCristina, ChircovNikolaos, BouropoulosCristina, CirtoajeNikolaos, ChamakosCristina, DumitriuNikolaos, KoukouzasCristina, FemoniNikolaos, KourkoumelisCristina, FornagueraNikolaos, NikolaidisCristina, MinnelliNikolaos, PapakostasCristina, NistorNikolaos, PolitakosCristina, Pinedo-RivillaNikolaos, TapoglouCristina, SavencuNikolaos, TziavosCristina, TozzoNikolaos, VaxevanidisCristina, TubaroNikolas, PodrazaCristina-Maria, TrandafirescuNikolaus, JägerCristóbal García, ParienteNikolaus, PapenbergCristobal, GarciaNikolay M., UshakovČrtomir, DonikNikolay, KovalCruceru, MihaiNikolay, MukhinCrystal S., ShinNikolay, PerovCsaba, HegedűsNikolay, PetkovCsaba, VáradiNikolic, MijoCtirad, UherNikolina, ZivaljicCuesta, AnaNikos, KarayiannisCui, HuangNikos, PnevmatikosCumaraswamy, VipulanandanNikša, KrstulovićCyril, BordreuilNileshkumar, DubeyCyrille Denis, TetougueniNiloufar, NilforoushanCyrille, RichardNiloufar, Raeis-HosseiniD. Andrew S., ReesNils G., WeimannD. C. Florian, WielandNils, EllendtDae-Cheol, KoNils, HaneklausDae-Hyun, ChoNima E., GorjiDae-Hyun, KimNima, MovahediDaeseung, JungNima, SayydiDafeng, ZhengNimer, MurshidDag, NoréusNina, BonoDagmar, GregušováNina, GunkelmannDagmar, SamesovaNina, OrlovskayaDahai, XiaNina, RecekDah-Shyang, TsaiNina, ŠtirmerDahu, ZhuNing, LiuDa-Hua, WeiNingshu, MaDaibing, LuoNiraj, ChawakeDaiji, KatoNiranjan, PatraDaisuke, IshiiNirav, JoshiDaisuke, MatsuuraNitesh, MittalDaisuke, TakajoNitilaksha, HiremathDaiva, MikucionieneNitin, ChandolaDaiva, ZeleniakieneNitu, SyedDale SwensonNitul, KakatiDale, FeldmanNives, VladislavićDalia, KaisarlyNoa, Lachman-SeneshDalia, SmailienėNoboru, YuasaDalibor, KocábNobufumi, UeshimaDalius, JuciusNobuhiro, MiuraDamian, BatoryNobuhisa, FujimaDamian, BebenNobuo, SasakiDamian, NakoniecznyNobuto, YoshinariDamian, NowakNobutoshi, OtaDamian, PaliwodaNoel, RodriguezDamiano, TaniniNoel, ThomasDamien, FabregueNoelia, Zagalaz-AnulaDamien, LeducNoemi Alejandra, Schclar LeitãoDamien, MertzNoemí, Aguiló-AguayoDamien, PrimNoémie-Manuelle, Dorval CourchesneDamir, GodecNokeun, ParkDamir, GrgurašNorbert A., HamppDamir, MarkucicNorbert M., NemesDamir, ValievNorbert, RadacsiDamir, VarevacNorberto, FeitoDan C., VodnarNordine, LeklouDan Ioan, StoiaNoriharu, YodoshiDan Jae, LinNoriko, TakegaharaDan Mihai, ConstantinescuNorman S., AllenDan T., BaborNoureddine, JouiniDan, BompaNoushin, NasiriDan, CristeaNozomu, SuzukiDan, DobrotăNubia, Zuverza-MenaDan, GheorgheNuggehalli M., Ravindra (Ravi)Dana, AshkenaziNuggehalli, RavindraDana, MiuNuno V., GamaDana, SchwarzNuno, AlvesDanaja, ŠtularNuno, LopesDandan, GuoNur Azam, AbdullahDanhong, WangNuria, GarcíaDani, DordevicNuria, García-CarrilloDania, OlmosNuria, Llorca-IsernDaniel E., Martínez-TongNuruzzaman, NoorDaniel N., BlaschkeOana Lelia, PopDaniel R., HinesOana, CadarDaniel Yiwei, TayOana, DodunDaniel, BaldomirOana-Maria, BolduraDaniel, BreiteObara, YuzoDaniel, Castro-FresnoOcean, CheungDaniel, ChateignerOchai, OklobiaDaniel, Dias Da CostaOcsana, OprisDaniel, ElduqueOctavi, Camps-FontDaniel, ErrandoneaOctavian Dumitru, PavelDaniel, FerrándezOctavian Mădălin, BunoiuDaniel, FologeaOctavian Tudorel, OlaruDaniel, FoucherOctavian, DuliuDaniel, FruchartOctavio, PereiraDaniel, GarcíaOday Ibraheem, AbdullahDaniel, KacikOdda, Ruiz De BallesterosDaniel, KajánekOfelia Cornelia, CorbuDaniel, KujawskiOfélia, AnjosDaniel, KurtzOfelia, Hernández-NegreteDaniel, KytyrOgyun, SeokDaniel, LaroucheOkhil K., NagDaniel, LepadatuOksana, ShishkinaDaniel, MarconiOksana, TananaikoDaniel, MaskellOksana, VelgosovaDaniel, MedyńskiOksana, ZholobkoDaniel, MolterOk-Su, KimDaniel, NeumaierOlaf, EnglerDaniel, Nieto GarcíaOlaf, HesebeckDaniel, OhOlaf, StenzelDaniel, OrtegaOlaf, Van Der SluisDaniel, PieniakOldřich, JirsákDaniel, PopaOldrich, SuchardaDaniel, RamosOleg E., GulinDaniel, RetaOleg G., MorozovDaniel, Rodríguez PradoOleg Igorevich, LebedevDaniel, Sanz PontOleg M., GradovDaniel, SaramakOleg V., GradovDaniel, SauerOleg V., MikhailovDaniel, SolaOleg Vladislavovich, LevinDaniel, TomaszewskiOleg, IlievDaniel, UrbánOleg, LunovDaniel, VizmanOleg, MishinDaniel, WeberOleg, SokolovDaniel, ZambonOleg, StolbovDaniela, BenedecOleg, UdalovDaniela, CascheraOleg, ZabeidaDaniela, GhicaOleh, AndrukhovDaniela, IannazzoOleksandr O., KurakevychDaniela, Ion-EbrasuOleksandr, GlushkoDaniela, LanariOleksandr, GryshkovDaniela, PappalardoOleksandr, TkachDaniela, PredoiOleksandra, VeselskaDaniela, RaduOleksiy V., PenkovDaniela, SovaOleksiy, PashkinDaniela-Roxana, Tamas-GavreaOleksiy, SydorukDaniele, BaniOlena I., OkhayDaniele, BarettinOlena, VolkovaDaniele, BattegazzoreOlexandr, GrydinDaniele, CangialosiOlga E., GlukhovaDaniele, DavinoOlga V., KaravaiDaniele, LosannoOlga, CegielskaDaniele, MantioneOlga, DymshitsDaniele, MartellaOlga, FedotkinaDaniele, PerroneOlga, GuskovaDaniele, RigonOlga, MakhlynetsDaniele, SofiaOlga, NetskinaDaniele, ZieglerOlga, SamoilovaDanielle, CoteOlga, SerenkoDaniil N., BratashovOlga, YakovtsevaDaniil, KolokolovOlga, ZinovievaDanijela, Jurić-KaćunićOlimpio, MonteroDanijela, MarovićOliver, ClemensDanil, PimenovOliver, MyersDanila, MosconeOliver, OpelDanko D., GeorgievOliver, SchierzDanko, BakarčićOlivier, BouazizDanko, ĆorićOlivier, ChassandeDanling, WangOlivier, DalvernyDanping, ChenOlivier, DezellusDanuta, Barnat-HunekOlivier, GuillaumeDanuta, MatykiewiczOlivier, PierronDanuta, MiedzińskaOlivier, ProuxDanutė, SližytėOlivier, ThomasDanyil, KovalovOlja, SimoskaDaqiang, GaoOlli-Ville, LaukkanenDaria, NikolaevaOluwatoosin Bunmi A., AgbajeDarina, DuplákováOluwole A., OlufayoDarinka, ChristovaOluyemi Peter, FaladeDario, CroccoloOlya, StoilovaDario, D’OrazioOmar, HamzaDario, De DomenicoOmid, GooranorimiDario, Di NardoOmid, KhalajDario, IljkićOmid, ZabihiDario, PasiniOmur, DagdevirenDario, SantonocitoOna, LukoševičienėDarius, BačinskasOncay, YasaDarius, MilčiusOndrej, DyckDariusz, BartkowskiOndřej, JankovskýDariusz, BartochaOndřej, KováříkDariusz, BochenekOndřej, KvítekDariusz, BorońskiOndřej, KylianDariusz, ChocykOndřej, ŽivotskýDariusz, FydrychOrazio, BaglieriDariusz, GarbiecOren, RazDariusz, HeimOreste, TaralloDariusz, JarzabekOrestis, VryonisDariusz, KarczOriana, PiermattiDariusz, KardaśOriele, PalumboDariusz, ŁukowiecOriol, PonsDariusz, MierzwińskiOrsolya, MolnarovaDariusz, MlynarczykOsama, AbdeljaberDariusz, OleszakOsama, EljamalDariusz, RozumekOsamu, KamiyaDariusz, RydzOsamu, UrakawaDariusz, SkabaOscar, CastañoDariusz, SkibickiOscar, Figueras-AlvarezDarko, DamjanovicOscar, Quevedo-TeruelDarko, IvančevićÓscar, Rodríguez-AlabandaDarko, MakovecOskar, ArmbrusterDarko, TibljasOskar, SachenkovDarrel, NicholasOssi, MartikkaDarya, GurinaOsvalda, SennecaDave, ChanOthman, NasirDavid A., DixonOthmeni, ChrifDavid Alexander, GregoryOtilia Ludmila, CintezaDavid E., JaramilloOurania, TsioulouDavid G., CalatayudOuti, SupponenDavid G., GarmireOvidijus, ŠernasDavid I., BigioOvidiu, CrisanDavid J., KukulkaOvidiu, NemeşDavid K., MaleOvidiu, VasileDavid M., BastidasOwais, WaseemDavid M., PierceOwen, ClarkinDavid Marín, GarcíaOwies M., WaniDavid Marrero, LópezOxana V., MagdysyukDavid W., WheelerOxana, KomovaDavid, Alique AmorPablo A., Garcia-SalaberriDavid, AradillaPablo Luis, López EspíDavid, AyrePablo Pérez, ZubiaurDavid, BeggPablo, AlbellaDavid, BerthebaudPablo, Álvarez-AlonsoDavid, BiedermannPablo, Fernández-YáñezDavid, BlancoPablo, LodeiroDavid, BombacPablo, Martin-RamosDavid, CameronPablo, Martín-RamosDavid, CorreaPablo, Pujadas ÁlvarezDavid, DixonPablo, Ramos-GarciaDavid, DobrockyPablo, Rodríguez-GonzálezDavid, DuQuesnayPablo, ZapicoDavid, EonPai, WangDavid, EscolanoPal, JovariDavid, FerryPál, SiposDavid, García-FresnadilloPalamandadige K. S. C., FernandoDavid, GarozPalilis, LeonidasDavid, GreenhalghPaloma, Rubio-de-HitaDavid, HernandoPan, GongDavid, JessonPanaghiotis, KaramanisDavid, JohnsonPanagiota P., GiannakopoulouDavid, KalfaPanagiotis (Panos), TsakiropoulosDavid, KlotzkinPanagiotis, BerillisDavid, LawPanagiotis, KyratsisDavid, López-DíazPanagiotis, LianosDavid, MelePanagiotis, MallisDavid, MorganPanagiotis, SismanisDavid, NečasPanagiotis, TheodorakisDavid, PaganinPanayotis, BenetatosDavid, PánekPanchao, YinDavid, PetrosyanPanchmatia, ParthDavid, RestrepoPandora, PsyllakiDavid, Revuelta CrespoPanita, MaturavongsaditDavid, San MartinPankaj, ChowdhuryDavid, Sánchez-GarcíaPankaj, KumarDavid, SchiraldiPanos, ApostolidisDavid, SinghPanos, EfthymiadisDavid, SkodaPanos, TsopelasDavid, TrunecPantelis G., NikolakopoulosDavid, WibowoPaola, CostanzoDavid, WoodwardPaola, Di MascioDavid, ZubiaPaola, Di PietroDavide, CavagnettoPaola, GandiniDavide, CeresoliPaola, GentileDavide, ForcelliniPaola, GiardinaDavide, MombelliPaola, LaurienzoDavide, NardiPaola, ManiniDavide, PalumboPaola, RizzarelliDavide, PapurelloPaola, RussoDavide, PorrelliPaola, SemeraroDavide, PriantePaola, StabileDavide, ProserpioPaola, TaddeiDavide, RavelliPaola, Viloria SáezDavide, RoccoPaola, ZuppellaDavide, SangiovanniPaolo S., ValvoDavide, TrutalliPaolo, BollellaDavor, GracinPaolo, BombelliDavor, StankoPaolo, CapparèDavorin, PenavaPaolo, ForaboschiDavydok, AntonPaolo, FormichiDawei, WangPaolo, GuidorziDawid, JanasPaolo, MarconciniDawid, RysPaolo, MarsiliDawid, StawskiPaolo, MatteisDawid, StefaniukPaolo, MercorelliDawid, SzurgaczPaolo, PiccardoDayakar Naik, LavadiyaPaolo, PrincipiDayakar, PenumaduPaolo, TrucilloDayun, YanPaolo, ZampieriDean, SmithParameswar, HarikumarDebadrita, PariaParanjayee, MandalDebananda, MohapatraParashu, KharelDebanjana, GhoshParaskevas, PapanikosDebin, XiaParaskevi, Yiouta-MitraDebora, PugliaPardis, RofouieDeborah, ChungParisa Pour Shahid Saeed, AbadiDebra F., LaeferParth, VashishthaDeclan, DevinePartha P., PaulDedy, SeptiadiParthiban, PazhamalaiDeepak Kumar, DubeyParthiban, RamasamyDeepak Kumar, PatelPascal, BergerDeepak Rajendra, UnunePascal, RayDeepak, GantaPascu, AlexandruDeepak, OjhaPasquale F., FulvioDeepak, VenkateshvaranPasquale, Di PalmaDeesy, PintoPasquale, GuglielmiDegang, XiePasquale, MarrazzoDejan, BrkicPasquale, Russo SpenaDelei, ShangPasquale, SaccoDelia, PatroiPasqualino, CoriglianoDelphine, BardPászti, ZoltánDelphine, Notta-CuvierPatric, DobronDemetre, EconomouPatrice, MélinonDemirci, SerpilPatricia, AparicioDemófilo, Maldonado-CortesPatricia, Diaz-RodriguezDeng, HongbingPatricia, DiogoDenis A., RomanovPatrícia, Freitas RodriguesDenis B., ZolotukhinPatricia, Kara De MaeijerDenis O., AlikinPatricia, Losada-PerezDenis, BenasciuttiPatricia, Poblete-GrantDenis, GentiliPatrícia, RijoDenis, GuilhotPatrick, GlouannecDenis, KarimovPatrick, HarknessDenis, KasymovPatrick, HaubruckDenis, KorneevPatrick, LittlewoodDenis, MusicPatrick, NavardDenis, NazarovPatrick, RichardDenis, OpraPatrick, ShambergerDenis, PolitisPatrick-Julian, MesterDenis, PustovoytovPatrik, ScajevDenis, RodriguePatrizia, CinelliDenis, RychkovPatrizia, ParadisoDenis, SabirovPatrizia, TrovalusciDenis, ShishinPatrycja, Domalik-PyzikDenis, SorninPatrycja, MakośDenis, VinnikPatryk, RozyloDenise-Penelope, KontoniPaul C., FitzgeraldDenitza, ZgurevaPaul F., EganDennis, CoonPaul F., SmithDennis, JanssenPaul Hm M., Van SteenbergeDenny, CoffettiPaul, BakerDer-Chyuan, LouPaul, BatchelorDercilio Junior, Verly LopesPaul, BereDerrick M., MottPaul, DerryDerun, ZhangPaul, FaragoDervis Emre, DemirocakPaul, JacobDesalegn Alemu, MengistiePaul, JaraDesirée, Rodríguez-RoblesPaul, LambertDesislava, StanevaPaul, LeidererDetlef, KlimmPaul, LuckhamDevaraj, ShanmukarajPaul, MotzkiDevid, FallianoPaul, PantanoDezso, BekePaul, SottaDhanada Kanta, MishraPaul, SundaramDhanasekaran, VikramanPaula, CoutinhoDhanjai, DhanjaiPaula, Malo De MolinaDharmapura Hanumantharaya K., MurthyPaula, OssowiczDharmendra K., YadavPaula, OulegoDhruba B., KhadkaPaula, Ruiz-HincapieDhruv, ThakarPaula, Villanueva-LlauradóDhruvajyoti, RoyPaul-Henri, HaumesserDi, SunPaulina, ChilimoniukDi, WangPaulina, KosmelaDi, WuPaulina, Lisiecka-GracaDi, ZhangPaulius, GriškevičiusDiakonova, NinaPaulmanickam, KoilrajDian, YuanPaulo Jorge, PalmaDiana, CiolaciuPaulo M. S. T., De CastroDiana, DudeaPaulo Nobre Balbis, Dos ReisDiana, HunPaulo, CaldasDiana, PaivaPaulo, CoelhoDiana, PetkovaPaulo, LozanoDiana, SanninoPaulo, MascarenhasDick, UhrlandtPaulo, MileoDidier, SnoeckPaulo, PalmaDidier, StuergaPaulo, PilotoDidier, VincentPaulo, SáDiederik, DeplaPaulo, ValeDiego Rafael Nespeque, CorreaPavan, AddepalliDiego Romano, PerinelliPavel A., LukyanovDiego Said, SchicchiPavel A., PodrabinnikDiego, AntonioliPavel N., BelkinDiego, CarouPavel S., KoroteevDiego, Infante-GarcíaPavel S., PostnikovDiego, MazaPavel S., ZelenovskiyDiego, Said SchicchiPavel, AtanasoaeDiego, TuroPavel, CtiborDiego, VergaraPavel, DemoDieter D., BosshardtPavel, DikoDieter W., EngelPavel, EvdokimovDieter, FrensePavel, GurikovDieter, SpiehlPavel, IvchenkoDietmar, AppelhansPavel, JandaDietrich, StoyanPavel, KopelDigby, MacdonaldPavel, KrakhmalevDigby, SymonsPavel, KrystynikDijana, VuleticPavel, LejcekDileep, SharmaPavel, LoikoDimitar, RadevPavel, LukacDimitra N., PapadimitriouPavel, MaňasDimitra, MichaPavel, MokrejšDimitri, BogdanovskiPavel, NovakDimitrio, KontziampasisPavel, NovákDimitrios A., DragatogiannisPavel, ProsrDimitrios C., ZografopoulosPavel, ReitermanDimitrios G., AggelisPavel, SalvetrDimitrios G., FatourosPavel, ŠilerDimitrios, BikiarisPavel, TofelDimitrios, ChaliampaliasPavel, UrbanekDimitrios, ChronopoulosPavlina, MateckovaDimitrios, DionysopoulosPavlo, MaruschakDimitrios, GiannakoudakisPavol, HvizdošDimitrios, GiliopoulosPavol, LengvarskýDimitrios, KontziampasisPavol, LipovskýDimitrios, LampakisPawan Kumar, MishraDimitrios, MihelogiannakisPawan, KaholDimitrios, PapoulisPawan, KumarDimitrios, TzetzisPawan, PathakDimitris A., ChalkiasPaweł P., PomastowskiDimitris S., AchiliasPaweł V., ŽukowskiDimitris, TsamatsoulisPaweł, BrykDimka I., IvanovaPaweł, ChmielarzDimos, TriantisPaweł, CzajaDina, DudinaPaweł, DunajDinara, SobolaPaweł, FalkowskiDinesh, GundapaneniPawel, FigielDing, ZhaoPaweł, GłuchowskiDinko, ĐurđevićPaweł, GrochDino, AquilanoPawel, HorodekDino, MusmarraPawel, JezowskiDionysios E., MouzakisPawel, JozwikDipali R., Bagal-KestwalPaweł, KaczyńskiDirk, BährePaweł, KochmańskiDirk, EngelbergPaweł, KossakowskiDirk, EnkePaweł, KozakiewiczDirk, HegemannPaweł, KrauseDirk, KucklingPaweł, ŁukowskiDirk, Lützenkirchen-HechtPawel, MarcDirk, PoelmanPaweł, MazierskiDirk, WilhelmPaweł, MieczkowskiDjordje, MandrinoPawel, NakielskiDmitri V., LouzguinePaweł, NiegodajewDmitri, MalakhovPaweł, NuckowskiDmitrii E., AndreevPawel, PlatekDmitrii, TayurskiiPawel, PohlDmitrii, ZaguliaevPawel, PolaczykDmitriy V., GunderovPaweł, RomanowiczDmitriy V., MakarovPaweł, SajkiewiczDmitriy, TitovPaweł, SkalskiDmitry O., DolmatovPaweł, SokołowskiDmitry Yurievitch, RazorenovPaweł, StelmachowskiDmitry, KharitonovPaweł, StochDmitry, SkachkovPaweł, TwardowskiDmitry, TagantsevPawel, WagnerDmitry, ValeevPawel, ZajacDmitry, WainsteinPaweł, ZmarzłyDmitry, ZimnyakovPayam, ShafighDmitry, ZinoveevPayam, ZarrintajDmytro, NesterovPedro F., MayuetDo Haeng, HurPedro J., RiveroDoaa, BondokPedro Raposeiro, Da SilvaDobrosława, KasprowiczPedro, BaboDobrota, DanPedro, Carmona MarquesDobryden, IlliaPedro, CintasDochshanov M., AldenPedro, CostaDoga, BilicanPedro, Fernández ÁlvarezDoh-Hyung, RiuPedro, GarcesDoina, RǎducanuPedro, LavelaDolić, NatalijaPedro, MorouçoDolores R., SerranoPedro, NetoDolores, Eliche QuesadaPedro, PinhoDomagoj, GlavinaPedro, PratesDomagoj, VrsaljkoPedro, Raposeiro Da SilvaDomen, TabernikPedro, RomeroDomenico, DalessandriPedro, RosaDomenico, FrattiniPedro, TavaresDomenico, LombardoPeer, HallerDomenico, MagisanoPei, ChengDomenico, PaparoPei, FengDomenico, SagnelliPei, YoutaoDomenico, TiganiPeifeng, SuDomingo, Morales-PalmaPeihong, NiDominic, WalesPeiyuan, GaoDominic, ZerullaPeiyuan, NiDominik, DoroszPeizhen, LiDominik, LogońPekka, TaskinenDominik, PaulkowskiPelagia, GawronekDominik, WyszynskiPeng, HeDominika, MadejPeng, JinDominikus, HeiftPeng, SongDominique, AgustinPeng, TengDominique, DalozPeng, WangDominique, De LignyPeng, ZhangDominique, HooglandPengfei, LiuDominique, LanielPengmin, PanDominique, LunterPengqing, LiuDomitilla, MandatoriPengtao, LuDonald, LucasPeng-wan, ChenDonald, LupoPeng-Yuan, WangDonald, PicardPenny, CominoDonald, RockwoodPentti, NieminenDonald, TrykPer, EklundDonata, Konopacka-ŁyskawaPer, NordbladDonatella, GiurannoPer, OhlckersDong Jin, YooPere, Barriobero-VilaDong Wook, ChangPere, CastellDong, JiangPere, MutjèDong, MengPeriyasamy, SivalingamDong, ZhouPetar, AntovDongbin, WeiPetar, ŽuvelaDongfang, ZhouPetcu, CristianDongfeng, XuePeter E., MurrayDong-Gyu, AnhPeter J., CollingsDonghuan, QinPeter J., MahonDong-joo, KimPeter R., LaityDongkyu, LeePeter Ross, UnderhillDonglai, ZhangPeter S., FedkiwDonglou, RenPeter, Ábrányi-BaloghDong-Man, RyuPeter, BaumliDongseok, SuhPeter, BurgholzerDongWon, JungPeter, FilipDong-Won, KangPeter, FinkelDong-Wook, HanPeter, FoxDonovan, LeonardPeter, FrankovskyDoojin, LeePeter, FröhlichDora, FotiPeter, FutášDora, MehnPeter, HodgsonDora, RoloPeter, JenkinsDoretta, CapsoniPeter, KotešDorian A. H., HanaorPeter, KrollDorin, LucaPeter, LiawDorin, RaduPeter, LoutzenhiserDorina Nicolina, IsopescuPeter, MikhailenkoDorina, LauritanoPeter, NiemzDoris, EhrtPeter, PanjanDorota, Czarnecka-KomorowskaPeter, PetrikDorota, DziurkaPeter, PetrovDorota, KaliszPeter, RodičDorota, Klimecka-TatarPeter, ŠevcDorota, KołodyńskaPeter, SherrellDorota, KowalczukPeter, ŠiffalovićDorota, MałaszkiewiczPeter, StarkeDorota, NeugebauerPeter, ŠugárDorota, Oniszczuk-ŚwierczPeter, Tamas-BenyeiDorota, Rutkowska-ZbikPeter, TatarkoDorota, SitkoPeter, TaylorDorota, ZawieskaPeter, UhlikDorthe, PosseltPetr, BelousovDouglas, DunlopPetr, FilipDouglas, IveyPetr, HaušildDowon, BaePetr, HenysDoyoon, KimPetr, KawulokDragana, DogančićPetr, LoudaDragana, GabrićPetr, MarcianDragana, KopitarPetr, MiarkaDrago, StrlePetr, MisákDragos, CozmaPetr, OpělaDragos, IsvoranuPetr, SedláčekDragos-Victor, AnghelPetr, ShibaevDražan, KozakPetr, ŠittnerDražen, JokićPetr, SlepickaDrialys, Cardenas-MorcosoPetr, SmolkaDriss, LahemPetr, ŠperkaDritan, SiliqiPetr, ŠtěpničkaDror, MalkaPetr, SyselDuanyi, WangPetr, VlcakDuarte, De Melo-DiogoPetra, MaierDuarte, MarquesPetra, PeerDubravko, RogalePetra, SchneiderDuc Duong, LaPetre, IonitaDuc Thanh, NguyenPetricǎ, VizureanuDuck-Ho, KimPetro, LutsykDuc-Kien, ThaiPetros E., TsakiridisDuc-Thang, VoPetros, KoutsovitisDu-Hyeong, LeePetros, PetrouniasDujian, ZouPetru, MihaiDulal, SahaPeyman, RahgozarDumitru, CebruceanPezhman, Zarabadi-PoorDumitru, MitricaPh. V., Kiryukhantsev-KorneevDumitru, OlaruPhilip W., WertzDumitru, VieruPhilip, EgbertsDũng (Dzung) Dinh, LuongPhilip, HackneyDuo, LiPhilip, HowesDuong, NguyenPhilip, KitchenDurga, ParajuliPhilipe, ColombanDushyant, KumarPhilipp, Del HougneDustin L., McIntyrePhilipp, GrützmacherDyi-Cheng, ChenPhilipp, HenckellDylan J., RichardsPhilipp, PreinstorferDziedzic, ArkadiuszPhilipp, SeilerDzigita, NagrockienePhilipp, SteinertDzmitry, ShcharbinPhilippe, BoisseEason, HildrethPhilippe, ColombanEbo, Ewusi-AnnanPhilippe, GléEckhard, KahlePhilippe, LambinEddy, SimoenPhilippe, LeclèreEden, TannerPhilippe, LecomteEdgar, SchäferPhilippe, PoullainEdit, CséfalvayPhillip, LewisEdith-roland, FotsingPhillip, VisintinEdmond, GravelPhimon, AtsawasuwanEdoardo, StaderiniPhornphimon, MaitaradEdouard, Rivière-LorphèvrePia, Lopez-JornetEduard-Marius, CraciunPia, Willberg-KeyriläinenEduardo, AnsorenaPiedad N., De AzaEduardo, CejuelaPier Carlo, RicciEduardo, EspinosaPier Giorgio, SchiaviEduardo, FortunatoPier Paolo, MancaEduardo, GarzonPier Paolo, RivieraEduardo, GuzmánPierantonio, De LucaEdward E., RemsenPierfrancesco, FioreEdward G., GillanPiergiorgio, GentileEdward J., GarbocziPiergiorgio, TataranniEdward Kah, Wei TanPierluigi, ColombiEdward, BednarzPierluigi, FanelliEdward, BormashenkoPiero, FerrariEdward, LoPierre Hubert, MutinEdward, MatiosPierre, D’AnsEdward, RoszykPierre, DumontEdward, SacherPierre, HerckesEdwin, BrandsPierre, MarechalEdwin, PengPierre, MargeritEdwin, TsoPierre, WeissEfraín, Carreño-MorelliPierre-Henri, CornuaultEfstathios E., TheotokoglouPierre-Jacques, LiotierEgidijus, KatinasPierre-Yves, Collart-DutilleulEgle, JotautienePierre-Yves, Le BasEgle, MiliaPierric, LemoineEgon, PavlicaPietro, AusielloEgor Y., PlotnikovPietro, BurrascanoEhrenfried, ZschechPietro, CalandraEhsan Naderi, KalaliPietro, CataldiEhsan, BhuiyanPietro, GaliziaEhsan, RahimiPietro, RussoEhsan, RezvaniPietro, StefanizziEkaterina P., AntonovaPihua, WenEkaterina, KomarovaPikee, PriyaEkaterina, KozlovaPilar, Mercader-MoyanoEkaterina, StepanovaPilar, PosadasEkaterini, MoschopoulouPilgyu, KangEkin, OzerPing, LiEkrem, OezkayaPing, XiangElad, PrielPingping, HanEladia M, Peña-MéndezPingping, YaoElaine, DunlopPingwei, LiuElba, MaurizPiotr A., GaudenElda, HegmannPiotr Marek, MarkowskiEleana, KontonasakiPiotr Paweł, WoyciechowskiEleftherios, AnastasiouPiotr, BałaElena D., ObraztsovaPiotr, BełdowskiElena M., Ivan’kovaPiotr, BorysiukElena Mercedes, Pérez-MonserratPiotr, CyganowskiElena N., KorostelevaPiotr, DobrzynskiElena O., NasakinaPiotr, DrygaśElena V., BalashovaPiotr, DybełElena V., UshakovaPiotr, DziegielElena Vladimirovna, BobrukPiotr, GaudenElena Yu., KramarenkoPiotr, GazdaElena Yu., PanchenkoPiotr, GębaraElena Yu., PikalovaPiotr, JaskułaElena, AnghelPiotr, JeleńElena, AtroshchenkoPiotr, KaminskiElena, BoldyrevaPiotr, KiełczyńskiElena, CastelliniPiotr, KnyziakElena, Cerro-PradaPiotr, KoniecznyElena, ColombiniPiotr, KurcokElena, CrottiPiotr, KuryloElena, DilonardoPiotr, KustrowskiElena, EfremenkoPiotr, KwaśniakElena, FerrettiPiotr, LackiElena, HelereaPiotr, LesiakElena, KorznikovaPiotr, LipiecElena, LenciPiotr, MackiewiczElena, LopezPiotr, MatczakElena, LucchiPiotr, MiluskiElena, ManailaPiotr, MitkowskiElena, MastalyginaPiotr, NarlochElena, MicheliniPiotr, OsuchElena, PolisadovaPiotr, PawlowskiElena, ScibiliaPiotr, PiszczekElena, ScutelnicuPiotr, ProchonElena, SitnikovaPiotr, ProchorElena, StoleruPiotr, RadziszewskiElena, TaranPiotr, RytlewskiElena, TocciPiotr, SiwakElena, VillaPiotr, SkubiszElena, VolkovaPiotr, SlomkiewiczElena, ZhitovaPiotr, SmarzewskiEleni, ToumpanakiPiotr, SzulcEleni, TsangouriPiotr, ZabierowskiEleonora, ConterositoPipsa, HirvaEleonora, MargheritisPlaiasu, GabrielaEleonora, PargolettiPlamen, KirilovEleonore, FröhlichPlaton, KaraseovElettra, PapaPlonska-Brzezinska, MartaElham, Hosseini NejadPo-Hsiang, WangEli, AghionPo-Hsien, LiElia, MarinPolina, KuzhirElia, RanzatoPoliraju, KalluruElias P., KoumoulosPolona Dobnik, DubrovskiElias, SakellisPooja, BasnettElide, NastriPoopalasingam, SivakumarElio Alberto, PérigoPooria, KhaliliElisa, BelluzziPooyan, AyarElisa, BoaniniPooyan, MakvandiElisa, FracchiaPosinasetti Nageswara, RaoElisa, GarbayoPostek, EligiuszElisa, GuazzelliPoulami, MajumderElisa, PanzariniPouya, Partovi-AzarElisa, PassagliaPrabaha, SikderElisa, ReboliniPrabhatchandra, DubeElisabet, SuarezPrabhusrinivas, YavvariElisabeta, SzerbPradeep, VaradwajElisabeta-Corina, SecuPradip, DalapatiElisabete C. B. A., AlegriaPradip, DasElisabete R., TeixeiraPragathi, DarapaneniElisabete Ribeiro, SilvaPragya, VermaElisabeth, HofmannPrakash G., KshirsagarElisabetta, CanettaPralok Kumar, SamantaElisabetta, DorePranab, BiswasElisabetta, TranquilloPranav, NawaniElisabetta, ZanettiPrannoy, SuraneniEliseo, BustamantePrasad, RautEliza Li Shan, FongPrasanna, NeelakantanElizabeth A., KoulaouzidouPrasant, SharmaElizabeth Dianne, RekowPrashant K., SarswatElliot R., BernsteinPrashant, JainElnaz, AfsharipourPrashant, KulshreshthaEloi, PinedaPrashant, SharmaEloisa, Salina BorelloPrashanth, Konda GokuldossElsa, GaravagliaPrateek, SaxenaElsa, LasseuguettePratheep Kumar, AnnamalaiElsisi, AlaaeldinPraveen, SherElvio, CarlinoPrem S., ThapaElvira, De Luna-BertosPřemysl, BeranElza M. M., FonsecaPremysl, VanekElzbieta A., PieczyskaPrimož, JelušičElżbieta, BociągaPrincipia, DardanoElżbieta, HorszczarukPritam, ShankhariElżbieta, MegielPritesh, ParikhElżbieta, PiesowiczPriyadharshini, PerumalElżbieta, Radziszewska-ZielinaPriyanka, KarnatiElżbieta, Stanaszek-TomalPriyanka, SharmaEmad, MaawadProtima, RauwelEmanoil, LinulPrzemysław, BrzyskiEmanuel, IonescuPrzemysław, BuczyńskiEmanuel, StriederPrzemysław, CzapikEmanuel, VamanuPrzemysław, GolewskiEmanuela, GattoPrzemyslaw, LedwonEmanuela, SperanziniPrzemysław, LisińskiEmanuele, BrunesiPrzemysław, MiąsikEmanuele, MauriPrzemysław, RybińskiEmanuele, RubilottaPrzemysław, SadowskiEmanuele, ZamperiniPrzemysław, SnopińskiEmeka, NkenkePrzemysław, StrzeleckiEmerson, CoyPshtiwan, ShakorEmil, EvinPu, DengEmil, SobólPui-Lam, NgEmil, SpišákPui-yan, HungEmile, HayePunita, KumariEmilia P., CollarPunnathat, BordeenithikasemEmilia, IglesiasPura Alfonso, AbellaEmilia, MihaylovaPuyang, ZhangEmilia, NeagPyeong Jun, YooEmilia, SikorskaPyung Soo, LeeEmilia-Adela, SalcaQamar, AbbasEmiliano, PasquiniQi, WangEmilie, CourbonQian, ZhangÉmilie, GaudryQiang, FuEmilija, PetronijevicQiang, YuanEmilio, BassiniQichi, LeEmilio, MartinesQigang, HanEmin, BayraktarQijin, HuangEmine, BakanQing, LuEmma, PaloQing-Feng, LiuEmmanouil, VougioukasQinghua, SongEmmanuel P., GeorgiouQinghuan, HuoEmmanuel, Atta-ObengQinglin, WuEmmanuel, GeorgiouQinglong, AnEmmanuel, KeitaQingsong, WangEmmanuel, LamourouxQingsong, YuEmmanuel, LegerQingxiang, WangEmmanuel, RamassoQinlong, RenEmmanuel, TopoglidisQinyi, WangEmma-Tuulia, NurmesniemiQiuchen, DongEmoke, PallQiuquan, GuoEmre, CinkilicQiwen, QiuEndre, NagyQixin, ZhouEneko, UkarQizhou, CaiEner, SemihQuan, LiuEnrica, ChiesaQuang-Cherng, HsuEnrico, AndreoliQuanlong, YangEnrico, BabilioQuark, ChenEnrico, BerrettiQuentin, GoossensEnrico, GiulottoQuentin, WilmartEnrico, LemmaQuim, TarrésEnrico, MarchettiQuoc Cuong, DoEnrico, MarsiliQuoc Tri, PhungEnrico, PanettieriRabee, ShamassEnrico, PetritoliRabih, MansourEnrico, SalvatiRachel L., SammonsEnrico, StortiRachele, CastaldoEnrico, TroianiRachelle, PalcheskoEnrique Fernandez, LedesmaRachman, ChaimEnrique, CasarejosRachmat Adhi, WIbowoEnsanya A. Abou, NeelRadek, ŠtefanEnshirah, Da’naRadhakrishna, PrabhuEnza, PanzardiRadhakrishnan, JagdheeshEnza, VitaleRadi, JishiEnzo Maria, VingoloRadian, PopescuEnzo, MartinelliRadionova, LiudmilaEoin, SyronRadoslav, SovjákEran, GreenbergRadosław W., MarudaErdal, UzunlarRadoslaw, JasinskiErdong, SongRadosław, LisieckiEri, YoshidaRadosław, ŁyszkowskiEric J. R., ParteliRadosław, MirskiEric W., NeumanRadosław, PodsiadłyEric, FlorentinRadoslaw, WiniczenkoEric, GaffetRadovan, BurešEric, GowansRadu Claudiu, FierascuEric, GuibalRadu Robert, PiticescuEric, LacosteRadu, AlbulescuEric, MartinRadu, ConstantinescuEric, QuarezRadu, GodinaEric, ShenRadu, MunteanEric, StarkeRadu, StefanoiuErica C., TeixeiraRadu-Dan, RusuErica L., MajumderRadu-Eugen, BreazErick, OgamRadu-Ioachim, ComaneciErik, CartonRaed, Abu-ReziqErik, KuhnRafael R., SolisErik, LascarisRafael, BareaErika, AdomavičiūtėRafael, Casquel Del CampoErika, CioneRafael, ComesañaErika, Coaglia Trindade RamosRafael, Leiva-GarcíaErika, DematteisRafael, Prado-GotorErika, HodulovaRafael, RodríguezErin, PatrickRafael, SantosErkan, AsikRafael, ShehuErkan, OterkusRafał J., WróbelErlantz, LizundiaRafał Jakub, WigluszErnesto, ChicardiRafał, AnyszkaErol, YilmazRafał, BielasErriguible, ArnaudRafał, BoguckiErtug, AydinRafał, JanusErwan, RauwelRafał, JanuszewskiErwin, RosenbergRafał, KobyłkaEryk, WolarzRafał, KowerdziejEsmaeil, Doustkhah HeraghRafal, KruszynskiEsmail, DoustkhahRafał, KrzywońEsperanza, DiazRafał, LongwicEsperanza, PeruchaRafał, MalinowskiEstela Oliari, GarcezRafał, MichalczykEsther, AmstadRafał, ReizerEsther, Bailon-GarciaRafał, WrobelEsther, RebollarRafał, WróblewskiEswar, ShankarRaffaele, CarliEtienne, DeveauxRaffaele, RiccòEugen Mircea, AnitasRaffaele, SepeEugen, AndreiadisRaffaello, PapadakisEugen, AnitasRaffel, BogenfeldEugen, AxinteRaghuraj Singh, ChouhanEugen, StamateRahim, AbdurEugene É., FeldshteinRahmat, EllahiEugene T., KennedyRahul, TrivediEugene V., CollaRaimundas, ŠiaučiūnasEugene, KoganRainer J., HebertEugenia, Eftimie TotuRainer, HahnEugenia, Fagadar-CosmaRainer, HeiseEugenia, GrecuRainer, HipplerEugenio, MeloniRainer, NiewaEugenio, PedullàRainer, NovotnyEugenio, Velasco OrtegaRaivo, SternEugeniusz, KodaRaj, KarthikEugeny, AlexandrovRaja, BalaguruEui Pyo, KwonRajab, AbousninaEui Soo, KimRajan, PatelEun Hea, JhoRajarajan, RamalingameEun Jeong, ChoRajavaram, RamaraghavuluEungje, LeeRajdeep, AdhikariEunice, CarrilhoRajender S., VarmaEunjong, AhnRajendra, DulalEuy, Sik JeonRajendran, MuthurajEva M. García, Del ToroRajesh, JhaEva M., Pérez-SorianoRajesh, MishraEva O. L., LantsoghtRajesh, ParsanathanEva Vargas, OrgazRajiv Ranjan, SrivastavaEva, FazakasRajkumar, PatelEva, FilovaRajkumar, VeluEva, KormaníkováRaju, KumalEva, MartinsRaju, SinhaEva, RemisovaRakesh, RanjanEva, RemišováRakhmonov, JovidEva, RentschlerRaktim, SarmaEva, SchiefersteinRalf, LachEva, ŠvábenskáRalph Rolly, GonzalesEvaggelia, PapiaRalph, BäßlerEvaldas, ŠerelisRalph, ChamberlinEvangelia, KaragianniRaluca, IanchisEvangelos, GavalasRaluca, IsopescuEvangelos, SkoulasRaluca, VodaEvdokia K., OikonomouRaluca-Giorgiana, ChivuEvelio, Teijón-López-ZuazoRama Krishna, ChavaEvgeni A., BezusRama Rao, TataEvgenia A., KovalevaRama, LayekEvgenia A., Tereshina-ChitrovaRamachandran Chidambaram, SeshadriEvgenii, BobasovRamalingam Venkat, Kalyana SundaramEvgenii, RoginskiiRaman, VedarajanEvgeniy, MersonRamez, HajjEvgeniy, PapynovRamezani, MajidEvgeny V., AbkhalimovRami, EidEvgeny, ApartsinRamin, KhoramniaEvgeny, BuntovRamin, RahmaniEvgeny, FilatovRamiro Rafael, Ruiz-RosasEvgeny, KatzRamón González, RubioEvgeny, NazarchukRamon, Escobar GalindoEvgeny, ParfenovRamon, Jerez-MesaEvgeny, PrusovRamon, PamiesEvgeny, ShafirovichRamon, TorrecillasEvgeny, ShirshinRamona, CimpoeşuEvgeny, TrofimovRamona, GalatusEvguenii I., KozliakRamūnas, ČesnavičiusEwa, GlowinskaRamune, ZurauskieneEwa, JondaRamy, GadallahEwa, KijenskaRamy, HusseinEwa, KorzeniewskaRan, NiuEwa, KowalskaRan, TaoEwa, OledzkaRan, ZhouEwa, Olewnik-KruszkowskaRandall M., ErbEwa, PopkoRando Tungga, DewaEwa, SkwarekRangasayee, KannanEwa, StodolakRanier Enrique, Sepúlveda FerrerEwa, TykarskaRaphael, BoichotEwa, WierzbickaRaphael, KampmannEwelina, BasiakRaphael, PiloEwelina, Kłosek-WawrzynRaphael, SchneiderE-wen, HuangRaquel Guillamat, PratsFabia, GrisiRaquel, Costa-AlmeidaFabian, MederRashid G., BikbaevFabian, StarsichRasim, AlosmanovFabian, WesthauserRasim, GuldikenFabien, BriffodRasoul, Esmaeely NeisianyFabio Maria, ColomboRastislav, VargaFabio, Di GioacchinoRathinam, BalamuruganFábio, FernandesRaul D. S. G., CampilhoFabio, GiudiceRaul J., BernardinoFabio, IucolanoRaúl, Ayuso-MonteroFabio, MaroniRaul-Augustin, MitranFabio, MazzaRauno, PääkkönenFabio, MinghiniRavi Ajitbhai, PatelFabio, PalumboRavi Anant, KishoreFabrice, PougoumRavi Chandra, DuttaFabrizio, BarberisRavi K., ArvapallyFabrizio, CalderaRavi Kiran, YellavajjalaFabrizio, GuzzettaRavi P., SahuFabrizio, MarraRavichandar, BabaraoFabrizio, SarasiniRavindranadh, KoutavarapuFaisal Ahmed, MemonRavitej, UppuFaisal, Mohd-YasinRawaz, KurdaFaisal, QayyumRawil F., FakhrullinFaisel, TubbalRaymond J., GorteFalk, WittelRazvan Ioan, PacurarFamulari, AntoninoRazvan, StefanFan Liang, ChanRazvan, UdroiuFan, YangReaz A., ChowdhuryFan, ZhangRebeca, Illescas MontesFanbin, MengRebecca, AnthonyFang-Chyou, ChiuRebecca, BostonFanghong, SunRebecca, LarsonFanghui, JiaRebecca, SaiveFangwei, ShaoRebeka, RudolfFangzhong, HuRechana, RemadeviFani, MantzouridouReda, YahiaouiFanny, CoumesReddy Ramireddy, ThrinathFaouzi, GhribRegina, Fuchs-GodecFarah, Asa’adRegis, BarilleFaraz, DeirminaRei, FurukawaFar-Ching, LinReihaneh, JamshidiFardin, KhabazRéka, BarabásFarhad, AslaniRemya P., NarayananFarhad, PargarRena C., YuFarhad, Rezai-AriaRena N., D’SouzaFarhana, AbedinRenata, BorisFariborz, KargarRenata, JastrzabFarnoosh, FarhadRenáta, OriňákováFarooq, SherRenata, WłodarczykFarshid, PahlevaniRenato Sante, OlivitoFarshid, SefatRenato, BuzioFarzad, Zangeneh-NejadRenato, FroidevauxFateh, FazeliRenato, GonçalvesFatemeh, AhmadpoorRenbo, YangFatemeh, AzhariRendang, YangFátima, TerneroRené M., RossiFausto, ZampariniRené M., WilliamsFawaz, KaseerRene, BuchetFawzy-Hosny, SamuelRené, HammerFayna, MammeriRene, LimbachFazhou, WangRene, LopezFederica, RaganatiRené, PyszkoFederica, SodanoRenee, GorehamFederico A., VivaRen-Jei, ChungFederico Maria, RubinoRen-Kae, ShiueFederico Simone, GobberRenxun, ChenFederico, AccorneroRen-Yeong, HuangFederico, AguayoReyes, AaseFederico, BarrinoReyes, MateoFederico, BellaReza Masoudi, NejadFederico, CarosioReza Taherzadeh, MousavianFederico, CecchiniReza, ArjmandiFederico, CesanoReza, BayatFederico, FoschiReza, HosseinpourpiaFederico, MazzucatoReza, JavaherdashtiFederico, MorettiReza, MolaeiFederico, VeronesiReza, SerajianFei, BinRezwanul, HaqueFei, DingRicardo Fernández, SerranoFei, LuRicardo J., Alves De SousaFei, PanRicardo M., GouveiaFei, ShenRicardo Mallavia, MarinFei, WangRicardo Manuel, SoutoFei, XuRicardo, AbejónFei, ZuoRicardo, BaptistaFeijun, QuRicardo, CastedoFei-Yi, HungRicardo, Do CarmoFelicia, ToleaRicardo, JimenezFelipe Vannucchi, De CamargoRicardo, VardascaFelipe, ConzueloRicardo, VergazFelipe, GándaraRicardo, Villa-BellostaFelisa, Reyes-OrtegaRiccardo, Di GianfilippoFelix, FritzenRiccardo, DonniniFelix, GoniRiccardo, FincatoFelix, SchacherRiccardo, MessinaFelix, WeberRiccardo, NobileFeng, CaiRiccardo, RussoFeng, ChenRich, MildrenFeng, FuRicha, ChaudharyFeng, LiRichard P., RayFeng, LinRichard T., WilkinsFeng, QiRichard, BaileyFeng, XuRichard, DrevetFenggui, LuRichard, DvorskyFengjiang, WangRichard, GlassFenglei, QiRichard, HorrocksFeng-Tso, ChienRichard, KlemmFerdinand, DobešRichard, KminiakFerdinand, ScholzRichard, LassFerdinando, SalvatoreRichard, MauliniFerenc, LezsovitsRichard, MoatFerenc, RonkayRichard, OleksakFermín, BañónRichard, OtisFernan, SaizRichard, PinčákFernanda Lopes, MottaRichard, RaspetFernando C. G., MartinhoRichard, ThackrayFernando C., Pérez-CárdenasRichie H. S., GillFernando Torres, AndónRico J., VictorFernando, CamposRikhav P., GalaFernando, CastroRiki, ToitaFernando, Fernández-LázaroRimantas, KacianauskasFernando, FerreiraRinat R., IsmagilovFernando, JulianRishi, GuptaFernando, LloretRita Bacelar, FigueiraFernando, OliveiraRita, AmbuFernando, Pacheco TorgalRita, FigueiraFernando, SimõesRitva, NäpänkangasFernando, SotoRizwan Abdul, Rahman RashidFernando, VeigaRizwan Rahman, RashidFevzi, KafexhiuRizwan Saeed, ChoudhryFilimon, AncaRoald M., TiggelaarFilip, ChylinskiRobabeh, JazaeiFilip, GórskiRobabeh, MoosaviFilip, PastorekRobert Catalin, CiocoiuFilip, SiskaRobert H., AguirresarobeFilip, StojcevskiRobert Horst, MeißnerFilipa, MoleiroRobert J., LadFilipa, SequeiraRobert J., MeierFilipa, SiopaRobert, BroniszFilipe, AlmeidaRobert, CackoFilipe, FerreiraRobert, CepFilippo Giammaria, PraticòRobert, CharvatFilippo S., BoiRobert, CzarnotaFilippo, CianettiRobert, EberleinFilippo, GiannazzoRobert, ErckFilippo, GiubileoRobert, GeitnerFilippo, LandiRobert, GuidoinFilippo, MangoliniRobert, HeinemannFilippo, ParisiRobert, KosturekFilippo, PieriniRóbert, KovácsFilippo, RossiRobert, KusiorowskiFilippo, SamperiRobert, LancasterFilippo, ZaniniRobert, MelchersFilomena, PiscitelliRobert, MroczyńskiFinian, McCannRobert, OwenFiona R., SmithRobert, OwsińskiFiona, SammlerRobert, PaszkowskiFiona, SchulzRobert, PealeFiore Pasquale, NicolettaRobert, PrzekopFioretta, AsaroRobert, SchennachFiras, KobeissyRobert, SchmittFirdaus, Muhammad-SukkiRobert, SekretFirman Mangasa, SimanjuntakRobert, StreubelFjodor, SergejevRobert, Texido BartesFlavio, FigueiraRobert, TomkowskiFlavio, RizzolioRobert, UlewiczFlavio, StochinoRobert, WojcieszakFlaviojorg, BartolomeuRobert, WojtyczkaFlaviu Mihai, Frigura-IliasaRobert, ZaleśnyFlora, FaleschiniRobert, ZieroldFlorence, CarrouelRoberta G., ToroFlorenci V., GonzálezRoberta, CipulloFlorent, AllaisRoberto A., BotoFlorent, GauvinRoberto Alonso, Gonzalez-LezcanoFlorentina, GolgoviciRoberto, Andresen EguiluzFlorian Ion, Tiberiu PetrescuRoberto, AvolioFlorian, ArbeiterRoberto, BernasconiFlorian, BittnerRoberto, BrighentiFlorian, HausenRoberto, CastillaFlorian, MartoïaRoberto, Castro-MuñozFlorian, PapeRoberto, CoppolaFlorian, SchulzRoberto, DÁmatoFlorian, SpieckermannRoberto, De SantisFlorian, StadlerRoberto, FeliceFlorian, WittemannRoberto, GristinaFlorica Adriana, JercaRoberto, LazzaroniFlorin, BorcanRoberto, Li VotiFlorin, IordacheRoberto, LópezFlorin, JurcaRoberto, MontanariFlorin, MiculescuRoberto, NisticòFlorin, OnisorRoberto, PastoreFlorin, PopaRoberto, PetrucciFlorina, CojocaruRoberto, PilotFlorina, TeodorescuRoberto, SalaFnu, SuriaminRoberto, SolimeneFolker, WittmannRoberto, SpinaFortunato, PezzimentiRoberto, SpotornoFotini, PetrakliRobin, KimFouad H., FouadRobinson, JamesFranc, MajdičRocco, Di GirolamoFranc, PerdihRocío Estefanía, Rojas-HernandezFranc, ZupaničRocío, PérezFranca, JonesRocío, Porras SorianoFranca, MorazzoniRodgers, MugumeFrancesc Xavier, EspinachRodica, PlugaruFrancesca, BettazziRodica, VlǎdoiuFrancesca, CirisanoRodica, VoicuFrancesca, DiomedeRodica, ZavoianuFrancesca, ErcoleRodica-Mariana, IonFrancesca, GarelloRodolfo D., MoralesFrancesca, GherardiRodolfo, PicchioFrancesca, LeonardiRodolphe, SonnierFrancesca, LionettoRodrigo, AntunesFrancesca, LorandiRodrigo, CruzFrancesca, LuziRodrigo, FrançaFrancesca, MastrottoRodrigo, HeyderFrancesca, NerilliRodrigo, MartinsFrancesca, PedoneseRodrigo, MorenoFrancesca, RossiRodrigo, NavarroFrancesca, RussoRoger, TannerFrancesca, SciarrettaRogerio, ManicaFrancesca, TessoreRohan, GalgalikarFrancesco Guido, ManganoRohan, MishraFrancesco Paolo, FanizziRohan, SomanFrancesco Paolo, La MantiaRohit, VoothaluruFrancesco, AlettaRoko, ZarnicFrancesco, BainoRoland K., ChenFrancesco, BasoliRoland, BejjaniFrancesco, BencardinoRoland, KleinFrancesco, BennardoRoland, MalliFrancesco, BertocciRolando, ChaconFrancesco, BoschettoRolands, KromanisFrancesco, BrandaRomain, LucasFrancesco, BucchiRoman A., NevshupaFrancesco, BuonamiciRoman B., TabakaevFrancesco, BuonocoreRoman, FediukFrancesco, CanestrariRoman, JaskulskiFrancesco, CaravelliRoman, KulaginFrancesco, ChiavaioliRoman, LaptevFrancesco, CiardelliRoman, MoravčíkFrancesco, CiccareseRoman, PuzniakFrancesco, ClementiRoman, StoklasFrancesco, ColangeloRoman, SturmFrancesco, CorderoRoman, SurmenevFrancesco, D’AmicoRoman, SzewczykFrancesco, Degli InnocentiRoman, TrochimczukFrancesco, DeloguRoman, Wan-WendnerFrancesco, Di CaprioRoman, WróblewskiFrancesco, FabbrocinoRomana, SchirhaglFrancesco, FerellaRomána, ZelkóFrancesco, FlorisRomeo, PatiniFrancesco, FocacciRomina, RegaFrancesco, FreddiRomualda, Bregier-JarzębowskaFrancesco, GucciRomualdas, KliukasFrancesco, GuidoRomualdas, NavickasFrancesco, InchingoloRomulus, TeteanFrancesco, LacconeRon H. J., PeerlingsFrancesco, LiguoriRonald James, ClarkeFrancesco, LopezRonald, HageFrancesco, Marotti De SciarraRonaldas, JakubovskisFrancesco, MauroRonaldo, AmaralFrancesco, ModicaRonan, MurphyFrancesco, MollicaRongcong, LuoFrancesco, NegroRong-Ho, LeeFrancesco, PampaloniRoni, ShneckFrancesco, PattiniRoong Jien, WongFrancesco, PieriRoozbeh, Abedini-NassabFrancesco, QuochiRoque Borinaga, TreviñoFrancesco, RobustoRoque, CalvoFrancesco, RossellaRosa, AgliataFrancesco, RuffinoRosa, De FinisFrancesco, SalvatoreRosa, DireitoFrancesco, ScotognellaRosa, Marat-MendesFrancesco, TodaroRosalía, PoyatoFrancesco, TornabeneRosana, Badía LaíñoFrancesco, TrequattriniRosaria, PuglisiFrancis K., ManteRosario, GarciaFrancisc, PeterRosario, PereiroFrancisco J., AparicioRosario, SerpicoFrancisco J., López-GarzónRoschger, AndreasFrancisco J., Romero-SalgueroRoscini, FrancescaFrancisco Javier, Cárcel-CarrrascoRose-Marie, LatonenFrancisco Javier, Madruga SaavedraRosemary, DziakFrancisco Javier, NavarroRoser, Sabater I. SerraFrancisco Javier, Navarro DomínguezRoshan, JosephFrancisco Javier, Otero-EspinarRoshini, RamachandranFrancisco Javier, Puerta MoralesRosica, MinchevaFrancisco Javier, Rodríguez-LozanoRosita, DianaFrancisco Javier, Sánchez-VelascoRositza, DimitrovaFrancisco Javier, TrujilloRosolino, VaianaFrancisco Javier, Velasco LopezRoss A., KernerFrancisco José Gomes, Da SilvaRossana, BellopedeFrancisco José Nunes, AntunesRossana, DimitriFrancisco M., MarquezRossella, ArrigoFrancisco, AgrelaRossella, CastagnaFrancisco, AlmansaRossella, SuraceFrancisco, Baeza-BrotonsRossen, TodorovFrancisco, CarmonaRostand Moutou, PittiFrancisco, Carrasco-MarinRostislav, ChotěborskýFrancisco, Cavas-MartínezRoumen, PetrovFrancisco, CuevasRouzbeh, DavoudiFrancisco, Do ValeRouzbeh, GhabchiFrancisco, EspínolaRowena H., BruggeFrancisco, Fernández MartínezRoxana, Racoviceanu (Babuta)Francisco, Fraga-LópezRoxana, StegaroiuFrancisco, LemosRoyce, FloydFrancisco, MárquezRu, LiuFrancisco, MartinsRuben Mas, BallestéFrancisco, Montero-ChacónRuben, Agustin-PanaderoFrancisco, RescalvoRuben, ForestiFrancisco, Ruiz-ZepedaRuben, GalindoFrancisco, SilvaRubén, LostadoFrancisco, SolanoRubén, Paz HernandezFrancisco-Javier, AyasoRubén, Rabadán-RosFranck, MeyerRuby, MejíaFranck, NicoudRuchika, BajajFranco, ConcliRüdiger W., SeidelFranco, DinelliRudinei, FiorioFranco, MarabelliRudolf, KieferFrançois O., LaforgeRudolf, PietschnigFrancois, BauerRudolf, SchneiderFrançois, Buyle-BodinRudy, KoningsFrançois, DucobuRuei-Sung, YuFrançois, GervaisRuey-Ching, TwuFrançois, MorencyRugved, LikhiteFrancois, RaultRui B., RubenFrançoise, ArnaudRui M. S., CruzFrank E., GoodwinRui Miranda, GuedesFrank W., ZokRui Pedro, MartinhoFrank, AbdiRui Vasco, SilvaFrank, AlexisRui, AlmeidaFrank, Alifui-SegbayaRui, GuerraFrank, BalzerRui, MarquesFrank, ClemensRui, MinFrank, CollinsRui, NevesFrank, CzerwinskiRui, PereiraFrank, KümmelRui, SilvaFrank, MissellRui, TamuraFrank, NiessenRui, XiaoFrans L., MullerRui, YuFrantišek, FojtíkRuifu, ZhangFrantišek, KováčRuiliang, LiuFrantišek, KrčmaRuipu, MuFrantisek, SoukalRuitao, QuFranz G., RammerstorferRuixiang, BaiFranz Josef, SeibertRuixue, WangFranz, RotersRuizhe, SiFranz, WeberRumana, HossainFranziska, SaxerRumyana, YankovaFranziska, SchmidtRun, HuFred, BurpoRun, ZhangFred, DavisRunchen, FangFrederic, AddiegoRupert, TscheliessingFrederic, DelbecqRupy Kaur, MatharuFrédéric, DelbecqRuriko, HatadaFrederic, DruesneRuslan, BarkovFrédéric, DumurRuslan, IbragimovFrédéric, GrondinRuslan, MuydinovFrédéric, LachaudRussell C., ReidFrédéric, LebonRussell, VarleyFrederic, LerougeRusso, EleonoraFrederick, RueggebergRustem, ValiullinFrederik, KotzRustem, ZairovFredrik, GlasserRūta, RimašauskienėFrey, MarionRutger, VrijdaghsFrieder, LucklumRuth, SiddallFua, TopuzRuzica, NikolicFuad, OsmanlicRyan E., MewisFulvio, CiriacoRyan Jin Young, KimFulvio, LavecchiaRyan, HolmesFumitake, FujiiRynno, LohmusFumitoshi, HirayamaRyo, KoikeFupeng, LiuRyoma, KitagakiFurong, TianRyszard, BuczkowskiFurqan A., ShahRyszard, DachowskiFutami, Nagano-TakebeRyszard, PawlakFuyao, YanRyszard, PecherskiFu-Yin, HsuRyszard, SitekFuzhen, PangRyszard, SkulskiFyodor, MalchikRyszard, WójtowiczGabor, CameliaRyszard, ZachGábor, DogossyRyuhei, NishiyabuGábor, KalácskaS. Amir Reza, BeyabanakiGabor, KovacsS. Habib, AlaviGábor, KozmaSaad, Al-SaadiGabor, MucsiSabata, MartinoGabor, PiskotySabina L., CampanelliGabor, SziebigSabina, AbbrentGabriel A., MontañoSabine, Kuchler-BoppGabriel J., AssafSabornie, ChatterjeeGabriel, AndreiSabri, AlamriGabriel, BatinSabrina, PrestoGabriel, FedorkoSabyasachi, GaanGabriel, FurtosSacco, LeandroGabriel, LokeSachin Maruti, ChavanGabriel, TestaSadanand, PandeyGabriela Iulia, DavidSadanori, AkitaGabriela, AristiaSadao, AdachiGabriela, CiobanuSadegh, GhourchianGabriela, CraciunSadig, AghazadaGabriela, LisaSae Byeok, JoGabriela, RutkowskaSaeed, KamaliGabriele, CervinoSaeed, MozaffariGabriele, GrecoSaeid Aghahossein, ShiraziGabriele, MelegariSage R., BauersGabriele, MilaniSagil, JamesGabriele, PitingoloSagrario Martínez, RamirezGabriele, RaabeSaianand, GopalanGabriella, LindbergSaid, LaasriGadi, BorkowSaïd, YagoubiGael Y., RochefortSaiful, IslamGaëtan, BuvatSaja M. Nabat, Al-ajrashGaetano Antonio, SciutoSajan Daniel, GeorgeGaetano, Di MinoSajid, Ali AnsariGaetano, GiuntaSajjad, GhobadiGaetano, IsolaSajjad, Seifi-MofarahGaetano, MarenziSakai, YuyaGaetano, PalumboSakdirat, KaewunruenGaetano, PaoloneSalah, MassoudGaetano, SquadritoSalar, BozorgiGaia, PellegriniSalim, HizirogluGajanan, GhodakeSalman, PervaizGajdzik, BozenaSalman, SiddiqueGalder, KortaberriaSalvador, EscobedoGalić, MirelaSalvatore A., PullanoGalina A., KovalenkoSalvatore, BocchieriGalina, GurievaSalvatore, GalloGalina, KurlyandskayaSalvatore, GrassoGanesh Dattatraya SarataleSalvatore, PastaGanesh Kumar, MeenashisundaramSalvatore, ScaccoGanesh, ShimogaSalvatore, VerreGang, FangSalwan, KhafajiGang, LiuSam, HinmanGang, WeiSam, LoflandGangadhar, AndaluriSamad, SepasgozarGao, ZhuangqiangSaman, HosseinpourGaojun, MaoSamantha E., WilnerGareth, HindsSamarthya, BhagiaGareth, NeighbourSameh, AttiaGargi, JoshiSameul Serna, OtálvaroGarikoitz, BeobideSamir, KhatirGarry, KerchSamir, KopacicGary J., DunderdaleSamira Esteves Afonso, CamargoGary, CaldwellSamira, NaghdiGary, HixSamjid H., MannanGary, HooperSamuel Clark, LigonGary, MankeySamuel, DorevitchGary, ThompsonSamuel, ObengGáspár Marcell, GáspárSamuel, ZelinkaGaspar, RegoSamy, MadboulyGauhar, SabihSandeep K., ChaudhuriGaurab, DuttaSandeep Sudhakaran, NairGaurav P., ShrivastavSandeep, MalyanGaurav, JainSandeep, PatilGauri, KhanolkarSandip M., VibhuteGavriel, ChaushuSandor, BodzasGaweł, ŻyłaSándor, SzénásiGbanaibolou, JomboSándor, ValkaiGeers, ChristophSandra, CruzGee-Soo, LeeSandra, Garcia-GallegoGejian, ZhaoSandra, PaszkiewiczGeminiano, MancusiSandra, PereiraGenadijs, SahmenkoSandra, RugonyiGenevieve, PalardySandrine, RicoteGennadiy, KolesnikovSandro, LongoGennady V., StepanovSandro, WartzackGennady, MiloshevskySandy, CochranGennady, ZebrevSang Don, MunGennaro Salvatore, PonticelliSang Won, KwakGennaro, GentileSang-Hyun, ParkGennaro, PonticelliSang-Keun, OhGennaro, ScarselliSangram, SamalGeno, MarcoviciSang-Ryul, ChaGeoffrey, MitchellSang-Yeop, ChungGeon Dae, MoonSanichiro, YoshidaGeorg, SchlickSanja, DimterGeorge Alexandru, NemnesSanja, Ercegović RažićGeorge G., AbashevSanja, Mahovic PoljačekGeorge Jayantha DiasSanjairaj, VijayavenkataramanGeorge Zhuo, TanSanjay, NimbalkarGeorge, AvgouropoulosSanjeev K., SharmaGeorge, EliadesSanjeev, KumarGeorge, MiloshevSanjiv, PrasharGeorge, PantazopoulosSanjoy, PaulGeorge, PapantonopoulosSanju, GuptaGeorge, PashosSankaranarayanan, SeetharamanGeorge, PonchakSankha, MukherjeeGeorge, TrakakisSanli, MovafaghiGeorge, VekinisSanny, VermaGeorge, VerrosSantanu, KhataniarGeorge, VoyiatzisSantanu, MukherjeeGeorge, WardehSantiago Ferrandiz, BouGeorge-Octavian, BuicaSantiago, GrijalvoGeorgia, ManikaSantiago, HerreroGeorgios C., PsarrasSantiago, OrregoGeorgios E., StavroulakisSantiago, Pozo-AntonioGeorgios N., AntonoglouSantosh S., PatilGeorgios Ι., GiannopoulosSantosh, KumarGeorgios, BartzasSanwu, WangGeorgios, DimitrakopoulosSaqib, HameedGeorgios, EleftheriadisSara, BernardiGeorgios, KritikosSara, CattaneoGeorgios, PapavasileiouSara, FerrarisGeorgios, SkordarisSara, Garzon HernandezGeorgy, FedorovSara, NocentiniGeorgy, LazorenkoSaranraj, KaruppuswamiGerald L., KnappSarath Mercedes Vega, GutiérrezGerald, HörnerSarath, KumaraGerard C., Van RhoonSarath, Vega GutierrezGerardino, D’ErricoSarbjit, SinghGerardo, GarcesSarmad, Al-AnssariGerasimos, PagiatakisSaroj, KandelGerd, DobmannSartaj Ahmad, BhatGerd, GrauSarunas, MeskinisGergő, TóthSasa V., DordevicGerhard, GrüllSaša, AhacGerhard, MullerSaskia, SchimmelGerhard, SwiegersSašo, GyergyekGermán F., De La FuenteSatender, KatariaGerman, CastilloSathiyaraj, KandhasamyGerrit, VermeirSatish, SakilamGesmi, MilcovichSatoru, OhisaGeta, CaracSatoshi, FujiiGetinet, WoyessaSatoshi, FukutaGhaeth, YassenSatoshi, KamiguchiGhanashyam, AcharyaSatoshi, KomasaGhazal, MoeiniSatoshi, NishimotoGheorghe, GurauSatoshi, YamaguchiGheorghe, MatacheSatoshi, YokoseGheorghe, NagitSaturnino Marco, LupiGheorghe, NechiforSaulius, DrukteinisGheorghe, OanceaSaulius, JuodkazisGheorghe, PaltaneaSaulius, VaitkusGheorghiţa, MitranSaulo, GeraldeliGheorghiu, Calin-IoanSaulo, MartelliGherlone Felice, EnricoSaurav, SarmaGhiara, GiorgiaSaurav, SorcarGholamhossein, LiaghatSaverio, CosolaGiacomo, OteriSaverio, MaiettaGiacomo, SaielliSayed Ehsan, SaghaianGiampietro, FarronatoScooter, JohnsonGian Andrea, RizziScott G., MitchellGian Carlo, CardarilliScott M., ThompsonGian Piero, LignolaScott, SchiffresGian, SongSebastian, BeyerGiancarlo, MaccariniSebastian, BürkleinGiancarlo, RamagliaSebastian, DahleGianfranco, CarotenutoSebastian, Feliú Jr.Gianfranco, Dell’AgliSebastian, FryskaGianfranco, NicodemoSebastian, GhinetGianfranco, PalumboSebastian, HärtelGiangiacomo, MinakSebastian, HenselGianina, DodiSebastian, JaegerGianluca, AccorsiSebastian, MrózGianluca, BoccardoSebastian, SchwamingerGianluca, CicalaSebastian, WachowskiGianluca, LandiSebastian, ZlotnikGianluca, PlotinoSebastiano, CandamanoGianluca, TondiSébastien, JoannèsGianluca, TrottaSébastien, PecqueurGianluigi, AlbanoSébastien, SanaurGianluigi, De FalcoSebastjan, GlinšekGianmaria, D’AddazioSedelnikova, MariyaGianni, GallelloSedov Vadim, StanislavovichGianpaolo, PapaccioSefl, VaclavGianpaolo, SavioSeid, KoricGianpaolo, VitaleSeigo, OhnoGianrico, SpagnuoloSeigo, OkawaGianvito, ScaringiSeiichi, KawaharaGideon, GraderSeiichi, TarutaGidion, TurualloSeisuke, AtaGierasimiuk, PawelSeiya, SuzukiGil, SerrancolíSejin, ChoiGill, DiamondSejoon, LeeGimyeong, SeongSelcuk, HazarGintarė, KručaitėSelda, OezkanGintaris, KaklauskasSemiramis, FriedrichGintautas, BagdziunasSenka, MeštrovićGintautas, SkripkiunasSeog-Jin, JeonGion, CalzaferriSeog-Young, YoonGiordano, FranceschiniSeok Jae, ChuGiorgi, ShtenbergSeok Su, SohnGiorgia, GhiaraSeokbin, LimGiorgia, GiovanniniSeokmin, KimGiorgio, FacchettiSeokmoo, HongGiorgio, LombardoSeokyoung, AhnGiorgio, MarrubiniSeong Jung, KwonGiorgio, SonninoSeong-Cheol, LeeGiorgio, SperanzaSeong-Gon, KimGiorgio, VilardiSeongHo, ChoiGiorgos, SkordarisSeong-Kyun, KimGiorgos, TzanetakisSeongwoo, GwonGiovanna, ConcuSeon-Jin, KimGiovanna, LonghiSepanta, HosseinpourGiovanna, OrsiniSeppo, LouhenkilpiGiovanna, PoggiSerafim M., OliveiraGiovanna, RotellaSerena, CoiaiGiovanni Francesco, SolitroSerena, DantiGiovanni Maria, SardiSerena, DuchiGiovanni, BolelliSergei A., DecterovGiovanni, BrunoSergei A., VasilievGiovanni, De BellisSergei Valentinovich, TrukhanovGiovanni, FalsoneSergei, AlexandrovGiovanni, GiacomelloSergei, ChekurovGiovanni, GuidiSergei, ChernyakGiovanni, LigorioSergei, KliminGiovanni, LucchettaSergei, KulinichGiovanni, MeneghettiSergei, PopovGiovanni, PappaletteraSergei, TverdokhlebovGiovanni, SempriniSergeiy Vasilievich, GnedenkovGiovanni, SignoreSergej A., UporovGiovanni, ZuccoSergej, GookGiriprasath, RamanathanSergej, MedvedGirish, RughooburSergejs, GaidukovsGirolamo, CostanzaSergejus, BorodinasĢirts, BūmanisSergey A., AdoninGirum Solomon, UrgessaSergey A., KukushkinGisela, OhmsSergey A., NikulinGiulia, FioravantiSergey A., ShevchikGiulia, FrediSergey Anatolievich, GhyngazovGiulia, GuidaSergey I., GutnikovGiulia, MagriSergey Leonidovich, SinebryukhovGiulia, MasiSergey M., AǐzikovichGiulia, MisseriSergey M., NovikovGiuliana, FaggioSergey N., GrigorievGiuliano, AngellaSergey V., DorozhkinGiuliano, GiambastianiSergey V., GudkovGiuliano, ZanchettaSergey Vyacheslavovich, KuryntsevGiulio, MarcheseSergey Yu., StefanovichGiuseppe Domenico, ArrabitoSergey, AksenovGiuseppe Fileccia, ScimemiSergey, BritvinGiuseppe Lo, GiudiceSergey, DubinskiyGiuseppe, BagnatoSergey, DvinskikhGiuseppe, BalduzziSergey, FedorovGiuseppe, BrandoSergey, IshutovGiuseppe, CantisaniSergey, KapranovGiuseppe, CavallaroSergey, KetovGiuseppe, CiaburroSergey, KidalovGiuseppe, CirilloSergey, KobtsevGiuseppe, CrucianiSergey, KonovalovGiuseppe, GranataSergey, KudryashovGiuseppe, LacidognaSergey, MedvedevGiuseppe, LazzaraSergey, MitryukovskiyGiuseppe, LoglioSergey, MoiseevGiuseppe, LoprencipeSergey, NikishinGiuseppe, MazzeoSergey, PaninGiuseppe, MuscasSergey, ShevtsovGiuseppe, MusumeciSergey, VinogradovGiuseppe, PeraleSergey, VinokurovGiuseppe, RutaSergey, ZherebtsovGiuseppe, SantarsieroSergi, Riera-GalindoGiuseppe, SciortinoSergio M. O., TavaresGiuseppe, StortiSergio M. R., LopesGiuseppe, TalòSergio, CapraraGiuseppina Pinuccia, CerratoSergio, CaputiGiuseppina, CamioloSergio, CiceroGiuseppina, NoccaSergio, GonzálezGiuseppina, PolinoSergio, LorenziGiuseppina, UvaSergio, RuggieriGiusy, CurcurutoSergio, SpinatoGladius, LewisSergio, Torres-GinerGleb, KozlovSergiu, CoseriGlen, DeaconSergiu, IordanescuGlikeria, KakaliSergiy, AntonyukGloria, Belén Ramírez RodríguezSerguei Petrovich, MurzinGloria, Huerta-AngelesSerguei V., KuznetsovGloria, MazzoneSerguei V., SavilovGloria, PenaSeth, DarlingGloria, PérezSeung Hwa, YooGloria, TabacchiSeung Uk, SonGnanendra, ShanmugamSeung Wook, KimGo, IgarashiSeung Yoon, RyuGo, InoueSeunghyun, KimGokcekaya, OzkanSeunghyun, LeeGokhan, SerhatSeung-Il, ShinGómez-Ior, BertaSeung-Jae, MoonGong, PanSeung-Jin, KimGonzalo, AlvarezSeungjun, ChungGopinathan, JanarthananSeungjun, KimGoran I., MiletićSeunguk, NaGoran, TurkSeungwon, LeeGoran, VukelicSeung-Won, OhGorana, PetkovićSeung-Yup, JangGorazd, LojenSeunho, JungGorazd, ŽibretSeval, GunduzGordana, MedunićSéverine, Le GacGorka, UrbikainSevero Raul, Fernandez-VidalGorka, Urbikain PelayoSeyed Ali, GhahariGorkem, MemisogluSeyed Amid, TahamiGoshtasp, CheraghianSeyed Farzad, AhmadiGosteva, AlevtinaSeyed Hamid Reza, SaneiGovind, ChilkoorSeyed Vahid, SajadifarGovindasamy, ManiSeyed, GhaffarGrant T., KirkerSeyed, Saeid Rahimian KoloorGrant, HillSeyedAbdolreza, SadjadiGranzow, TorstenSeyyedeh Azar, Oliaei MotlaghGray, LinShaden, KhalifaGrazia Giuseppina, PolitanoShaghayegh, KhaniGrazia, AccardoShahabaldin, RezaniaGraziella, MalandrinoShahed, RasekhGrażyna, AdamusShahin, HomaeigoharGrazyna, LewandowiczShahir, YusufGrazyna, Simha MartynkovaShaikh Nayeem, FaisalGreeshma, ThrivikramanShailendra, RajputGreg A., BrewerShamus, McNamaraGreg, ShawShanaka, BadugeGreg, WhiteShangquan, WuGrégoire, LéonardShangran, XieGregor, KravanjaShanhong, WanGregor, ŽerjavShanmugam, MuruganandanGregori, CasalsShannigrahi, SantiranjanGregorio F., OrtizShannon S., NicleyGregorio, GarcíaShanoob, Balachandran NairGregory F., MethaShaohua, ChuGregory S., NusinovichShaohua, JiangGregory, CaputoShao-ju, ShihGregory, GilesShaolin, ZhangGregory, PandraudShaolong, SunGrégory, PouriéShaopin, SongGreta, AdamczykShaoqing, WangGreta, FaccioShaowei, YangGrigory N., ChurilovShaoyang, LinGrün Nicole, SommerShao-Yi, HsiaGrzegorz Ludwik, GolewskiSharon, Kao-WalterGrzegorz, AdamekShashi Kant, ShuklaGrzegorz, CempuraShashi, BhuckoryGrzegorz, ChladekShatadru, ChakravartyGrzegorz, CholewinskiShaun, McFaddenGrzegorz, CieslarShayan Fakhraei, LahijiGrzegorz, ĆwikłaShazada M. U., KhanGrzegorz, DerczSheik, TanveerGrzegorz, DombekShengchuan, WuGrzegorz, GrabowskiShengdun, ZhaoGrzegorz, GumiennyShengfu, YuGrzegorz, KłosowskiShengguan, QuGrzegorz, KowalukSheng-Heng, ChungGrzegorz, KrólczykShenghua, WuGrzegorz, LesiukShengjun, ZhouGrzegorz, LisakShengli, JinGrzegorz, MatulaShengtai, ZhouGrzegorz, MieczkowskiSheng-Wei, FengGrzegorz, MoskalShengwen, TangGrzegorz, NowakSherif, El-SaftyGrzegorz, RaniszewskiSherif, FakherGrzegorz, RogalskiShery, ChangGrzegorz, SkrabalakShi, BaiGrzegorz, SochaShiao-Wen, TsaiGrzegorz, StradomskiShib Shankar, BanerjeeGrzegorz, StruzikiewiczShibo, LiGrzegorz, ŚwitShichen, SunGrzegorz, WiniarskiShideh, Kabiri AmeriGrzegorz, WójcikShifeng, WangGu, XuShifra, LevartovskyGuan, LinShigehiko, YumuraGuang, RenShigeru, HamadaGuanghui, ZhuShigeyuki, YamadaGuangjun, GaoShih-Ching, WuGuangkui, XuShih-kang, LinGuang-Liang, FengShih-Wei, ChoGuanglin, XiaShih-Yun, ChenGuang-Ling, SongShijie, RenGuangming, ChenShikha, ShresthaGuangwei, YangShikinaka, KazuhiroGuangxian, LiShikuan, SunGuangxue, ChenShiladitya, PaulGuangyi, MaShilun, FengGuangyong, SunShima, PilehvarGuangyu, QiuShima, TaheriGuanjie, HeShin Hye, ChungGuankui, WangShinichi, MochizukiGuannan, ZhangShin-Ichi, OhiraGuan-Ting, PanShinichi, TashiroGuanwu, WangShinichiro, AdachiGuanyu, ZhouShinji, AndoGudrun, AuffermannShinji, OgiharaGudrun, BrucknerShinji, TakemotoGuebum, HanShintaro, SukegawaGue-Hyun, KimShin-Tson, WuGuendalina, ZuccariShinya, InazumiGueorgui K., GueorguievShin-Ya, KitamuraGuha, ManogharanShipeng, WenGui, WangShiplu, SarkerGuian, QianShirin, KaboliGuido, MarsegliaShiro, KubukiGuido, OriShirsendu, SikdarGuido, PanzarasaShiuh-Chuan, HerGuido, PaolicelliShiuh-Hwa, ShyuGuido, TociShiv K., SharmaGuijian, XiaoShiva, MohajerniaGuilhem, PoyShivakant, ShuklaGuilherme, GasparShivraj, KarewarGuillaume, CouégnatShiyang, TangGuillaume, SavelliShlomo, MagdassiGuillem, Domènech-GilShmuel, RyvkinGuillermo A., RiverosShmuel, SamuhaGuillermo Pradíes, RamiroShoaib, SarfrazGuillermo, Guerrero-VacaShoichiro, SumiGuillermo, MonrósShoshan Tamar, AbrahamiGuillermo, Riesco MunozShotaro, NishitsujiGundula, Schulze-TanzilShou-Heng, LiuGungun, LinShouhu, XuanGunnar, KlosShouke, YanGunnar, SeideShou-Mei, XiongGunnar, SuchaneckShravan, KairyGuntae, KimShreyas, KuddannayaGunter, HeymannShrihari, SankarasubramanianGünther Josef, RedhammerShruti, VyasGuntram, WagnerShuai, CaoGuo, JiangShuai, LouGuodong, GohShuai, ShaoGuojun, QiShuai, WangGuoli, TuShuang, LuGuolong, LuShuanghao, ZhengGuolong, ZhaoShuanglong, LiuGuoming, WangShu-Chuan, LiaoGuo-Qing, HuShu-feng, YeGuosheng, HuangShuhei, TakahashiGuoxin, XieShuilong, ShenGuoxing, MiaoShujian, ChenGuoxing, SunShuko, SuzukiGuoying, DongShuli, FanGuozhao, JiShun-Peng, ZhuGuozhen, LiuShunsuke, MuraiGurjot, DhaliwalShuo, ChenGustavo Filipe, PintoShuping, PengGustavo Gonçalves, DalkiranisShuqing, SunGuy A., PluvinageShuting, ChenGuya Diletta, MarconiShuvra, SinghaGwang Goo, LeeShuwen, ZengGyanender P., SinghShweta, AgarwalaGyani Shankar, SharmaShweta, GoyalGyeong Hee, RyuSiamak Farajzadeh, KhosroshahiGyeongcheol, ChoeSibele B. C., PergherGyorgy, DeakSiddharth S., GautamGyorgy, SzekelySiddharth, GautamGyubaek, AnSiddharth, KesharwaniGyuyong, KimSidney, LinHadi, AryanSidorov V., NikolayHadia, AlmahliSiegfried Bernhard, MenzelHaejin, BaeSiemowit, MuszyńskiHaeng-Ki, LeeSiham, AmirouHae-Seok, LeeSi-Il, SungHae-Won, KimSílvia Castro, CoelhoHafeezullah, MemonSilvia De, La FlorHafiz Waqar, AhmadSílvia Morais, PereiraHai, XinSilvia, AngelovaHai, ZhangSilvia, BarbiHaibo, XieSilvia, Bittolo BonHaibo, YuSilvia, CarlottoHaifei, LvSilvia, CasassaHailong, LyuSilvia, DanteHaim, AbramovichSilvia, FarèHaim, GrebelSilvia, FioreHaitao, CuiSilvia, LogozzoHaiwen, ZhuSilvia, PatachiaHai-Xing, FangSilvia, SantiniHaiyan, ZhaoSilvia, SchintkeHaiyang, ZouSilvia, VasiliuHaiyuan, ZhangSilvija, KukleHajo, DieringaSilvino Dias, CapitãoHakan, BildirirSilvio, MeloniHalenur, KurmusSilvio, OsellaHalina, GarbalinskaSilviu, NastacHalina, KrawiecSilviu, PolosanHamdan S., AlghamdiSima, KashiHamdy, IbrahimSimas, RackauskasHamed, MalekmohammadiSimeng, LiHamed, MansooriSimon A. M., HespHamed, SohrabpoorSimon, CarstensHamid Reza, LashgariSimon, DenningtonHamid, DoostmohammadiSimon, HigginsHamid, ZaidiSimon, PascalHamidreza Yazdani, SarvestaniSimon, RogersHamidreza, AnajafiSimon, SteinbergHamidreza, DehghaniSimon, TrosienHamza, SamouhSimona Liliana, IconaruHamzeh, HaghshenasSimona, BadilescuHamzeh, KashaniSimona, BungauHan Cheol, ChoeSimona, FiliceHan Eol, LeeSimona, MartinottiHan, WangSimona, MiclausHan, ZhangSimona, MorariuHana, CharvátováSimona, StrnadHana, JirkováSimona, VasileHana, OvčačíkováSimone, CiampiHanfei, MeiSimone, DonadelloHang Nga, MaiSimone, GalloHang, YeSimone, ManciniHangfeng, ZhangSimone, VenettacciHang-Sheng, YangSimone, ZanottoHaniyeh, FayazfarSimonetta, BoriaHanjong, PaikSimonetta, TutiHanki, KimSinan, SuHanna, DodiukSindy, Seara-PazHanna, PazniakSiqi, GuoHannah K., LiberatoreSiqi, ShiHannah, LeeseSitarama, KadaHannes, RijckaertSitaraman, KrishnanHannu-Pekka, KomsaSithara S., NairHanoch Daniel, WagnerSithara, SreenilayamHanqing, CheSiu Fung, YuHans, JanssenSivasambu, BohmHans, RoggendorfSkander, HathroubiHans-Georg, SchweigerSlađana, MilardovićHans-Joachim, KrauseSlah, YaacoubiHanumantha Rao, MadalaSlawomir M., KaczmarekHany, HassaninSławomir, BorymskiHanying, ZhaoSławomir, CzarneckiHao, HaiSławomir, DreslerHao, WangSlawomir, FrancikHao, YangSlawomir, GrabowskiHao, YuSławomir, JakiełaHao, YuanSławomir, MilewskiHaochun, ZhangSlawomir, SwiradHao-Hueng, ChangSlobodan, VukicevicHaohui, XinSmolík, LubošHaolong, ChenSneha, SamalHaoyan, WeiSnježana, Firšt RogaleHaoyu, WangSnježana, Mardešić-BrakusHaozhi, PanSnjezana, Tomljenovic-HanicHaque A. B. M., TahidulSofia A., KoriliHaradhan, KolyaSofia, MasiHaralampos N., MirasSofia, MorozovaHarald, RennhoferSofia, PapadiochouHaribabu, PalneediSofia, RealHarikrishnan, JayamohanSohail, YasinHarish, KumarSoham, GhoshHarishkumar, MadhyasthaSoheil, GohariHarold, PhilipsenSoheil, JafariHaroldo T., HattoriSoheil, SaediHarry, CoulesSoheil, SolhjooHarry, KambezidisSohichi, HiroseHarsh, VardhanSo-Hye, ChoHarshkumar, PatelSo-Hyoun, LeeHartmut R., FischerSolange, MagalhãesHartmut, GliemannSolmoi, ParkHartmut, WitteSomasekhar R., ChinnadayyalaHaruaki, KitagawaSomasundaram, PrasadhHary, DemeySomen K., BhudoliaHashimoto, ChikanoriSompop, BencharitHassan, GhadbeigiSon Long, HoHassan, JavedSon Thanh, NguyenHassan, MaherSong, JoonhyukHassane, LgazSong, ZhangHatice, MutluSongkil, KimHåvard J., HaugenSonglin, DingHawreen Hasan, AhmedSonglin, ZhangHayet, DjelalSongwen, TanHazel, AssenderSonia, CarabineiroHe, HuangSónia, SimõesHe, LiSonja, SielkerHeather A., MurdochSoo-Hwan, ByunHeather L., BowlingSoo-Hyun, JooHeather, PowellSoon-Hong, KwonHechuan, LiSophie, BriffaHector Andres, TinocoSophie, CoxHéctor, BeltránSophiya I., PechenyukHéctor, MigallónSoram, OhHédi, HamdiSören, BartelsHeejin, JangSören, ÖstlundHee-Jo, LeeSori Ion, JingaHeghes, BogdanSorin, MihaliHegyi, AndreeaSossio Fabio, GrazianoHeidi, PiiliSotiris K., HadjikakouHeinz, PalkowskiSotiris, SotiropoulosHeinz, SturmSotomi, IshiharaHeinz, SurbeckSougata, MallickHeinz, WinklerSougata, RoyHélder, PugaSoundaram Jeevarathinam, AnanthakrishnanHelena Cristina, VasconcelosSowmya, SrinivasanHelena Otmačić, ĆurkovićŠpela, TrafelaHelena, FelgueirasSpiros, ZinelisHelena, Oliver-OrtegaSpyridon N., PapageorgiouHelena, Otmačić ĆurkovićSpyridon, MourtasHelena, RyšlaváSpyridon, NtougiasHélène, Serier-BraultSpyros, GallisHeli, KangasSpyros, PapaefthymiouHeli, LiuSravya, TekumallaHella-Christin, ScheerSrecko, StopicHelmut, HollSreco D., SkapinHelmut, KlöckerSreejith, NanukuttanHelmut, RiedlSreejith, SivaramapanickerHelmut, RitterSreekar Babu, MarpuHem, MotraSri Ayu, AnggrainiHemant, ChoudharySridhar, VadahanambiHemraj, YadavSriharsha, VenuturumilliHendra, HermawanSrinivas, GadipelliHendrik, KosslickSrinivasan, BhuvaneshHeng Li, HuangSriram Praneeth, IsanakaHeng, LiSriram, MalladiHengxu, SongSriramya D., NairHenri, VahabiStamatia, KarakouliaHenriette, SzilagyiStamatis, BoyatzisHenrik Hovde, SonstebyStancekova, DanaHenrik, PedersenStanislav, LenartHenri-Pierre, LassalleStanislav, MelnikovHenrique, De Amorim AlmeidaStanislav, RuszHenrique, GomesStanislaw, JozwiakHenryk, GrajekStanisław, JóźwiakHenryk, KaniaStanisław, NogaHeon-Ho, JeongStanisław, PietrzykHeonjune, RyouStanko, PopovićHerbert, PfnürStanley Udochukwu, OfoegbuHeriberto, Perez-AceboStathis C., StirosHerman, PotgieterStathis, ΚaliviotisHermann, EhrlichStefan Ioan, VoicuHermis, IatrouStefan J., EderHernán, GonzálezStefan, BaudisHernando III, SalapareStefan, BossmannHerng, Tun SengStefan, BringuierHervé, ReychlerStefan, ChassaingHesam, PouraliakbarŠtefan, ČikošHeshmat, NoeiStefan, CsakiHeuken, MichaelStefan, FlegeHeura, VenturaStefan, JanzHicham, JabraouiStefan, KarlssonHideaki, IwaokaStefan, KolevHideaki, KatogiStefan, KöstlerHideaki, TakahashiStefan, KrakowiakHideaki, YamadaStefan, KranzHideaki, YasuharaStefan, PanglischHideki, HayashiStefan, PersijnHideki, KyogokuStefan, RungHideki, NakazawaŞtefan, ŢăluHidetada, HirakawaStefan, TanglHidetaka, KawakitaStefan, WagnerHideto, TsujiStefan, WolnyHidetomo, UsuiStefan, ZechelHidetoshi, TeramotoStefana, MiliotoHideya, KawasakiStefan-Andrei, IrimiciucHikaru, KoboriStefania, ImperatoreHilario, VidalStefania, LiuzziHimanshu, AroraStefania, MarzoratiHimanshu, GoelStefania, MitolaHimanshu, KathuriaStefania, NardecchiaHiroaki, TatsumiStefania, SilviHirofumi, InoueStefania, StoleriuHirofumi, MiyajiStefania, ZappiaHirohiko, HoujouStefano, AgnoliHirohito, OgasawaraStefano, BellucciHiroki, AkasakaStefano, BerettaHiroki, ManoStefano, BonduàHiromasa, HasegawaStefano, CaporaliHiromu, SaitoStefano, CicchiHironobu, TaharaStefano, ColumbuHironori, NakajimaStefano, CrespiHiroo, UchidaStefano, EramoHiroshi, IkedaStefano, FioriHiroshi, KajiyaStefano, FocaroliHiroshi, KoibuchiStefano, FranginiHiroshi, MaiwaStefano, GazzottiHiroshi, OkadaStefano, GrolliHiroshi, TagawaStefano, GuarinoHiroshi, YoshiharaStefano, LaiHiroyasu, KoizumiStefano, LauretiHiroyo, SegawaStefano, LettieriHiroyuki, KanzakiStefano, ManfrediniHiroyuki, MiyamotoStefano, MarianiHiroyuki, MuramatsuStefano, MaschioHiroyuki, OkamotoStefano, PaganoHiroyuki, TakeiStefano, PerilliHiroyuki, TatenoStefano, PisaHirpa G., LemuStefano, RossiHisham, AboulfadlStefano, SalvatoriHitoshi, ShikuStefano, SgobbaHo Jun, SongStefano, SivolellaHoa, Le QuynhStefano, ValvanoHoang Nam, PhanStefano, Vecchio CipriotiHoang V., ChuyenStefanos, MourdikoudisHoang, NguyenSteffen, BergHoang-Phuong, PhanSteffen, WitzlebenHocine, BougdahŠtěpán, HýsekHocine, ChalalStepczynska, MagdalenaHocine, SiadStéphane, DanieleHofko, BernhardStephane, KenmoeHogeon, SeoStephane, PanierHojeong, JeonStephanie, FlorczykHojong, ChoiStephen A., BagshawHolger, FiedlerStephen A., CrossHolger, WatterStephen Peter, StagonHone-Zern, ChenStephen U., EgarievweHong Chul, MoonStephen, FosterHong Joo, KimStephen, HenryHong, LeiStephen, HoathHong, MengStephen, JosephHong, PengStephen, SkinnerHong, QiStephen, WalleyHong, VuStephen, WorrallHong, ZhaoSteven M., TommasiniHong, ZhouSteven R., SchmidHongan, MaSteven W., BucknerHongbo, FengSteven, BabaniarisHongfeng, XieStijn, MertensHong-Gu, JooStoyan, GutzovHongjian, DuStoyan, SlavovHongjing, WuStuart Leigh, PhoenixHongjun, WangStuart, StockHongkun, LiStyliani, PapatzaniHongli, HuSubhankar, SinghaHongliang, HeSubramanian, SundarrajanHongqin, MaSubrata Deb, NathHong-Sheng, QiSubrata, GhoshHongshun, LiuSubrata, SarkerHongwei, BaiSuchinder K., SharmaHongwei, GuoSuchol, SavagatrupHongwei, LiuSuchorzewski, JanHongye, YangSu-Chul, YangHongyu, LiSudarson Sekhar, SinhaHongyu, LiuSudhanshu, NahataHongze, WangSudhir, RavulaHongzhi, LiuSudip, MondalHooman, SabarouSudip, MukherjeeHoon Eui, JeongSugrib Kumar, ShahaHorațiu Alexandru, ColosiSugrib, ShahaHoraţiu, MoldovanSuhrid, DeshmukhHoratiu, RotaruSujat, SenHoraţiu, VermeşanSujeet, KumarHorea, FeierSujeong, LeeHoria, CalniceanuSujit, DasHoria, ConstantinescuSujith, MangalathuHossam, KishawySuk Ho, BhangHossein, Cheraghi BidsorkhiSukanta, BhowmickHossein, DerakhshanSukhoon, PyoHossein, EslamiSukjoon, HongHossein, JahromiSuk-Won, HwangHossein, MohammadiaraniSuman Kumar, AdhikaryHossein, TaheriSumanta, SahooHouliang, TangSuming, ZhuHouwen, PanSumit, MurabHouyang, ChenSumit, SachdevaHrishikesh, DasSumita, GoswamiHristo, PenchevSun Woog, KimHrvoje, DraganićSunand, SanthanagopalanHrvoje, GlavasSunanda, SainHsiharng, YangSung Bo, LeeHsing-I., HsiangSung Cik, MunHsuan-Kai, LinSung Hwan, HongHsuan-Ling, KaoSung Woon, ChaHu, WangSung Woong, ChoiHua, WeiSung-ae, SonHuai, YangSung-Chul, BaeHuaijun, LinSungchul, YangHuaishan, WangSung-Gul, HongHualing, HeSunghan, KimHuang, Chien WeiSungho, SongHuang, ZengSung-Hoon, KangHuang-Hsing, PanSung-Hwan, JangHuan-Hsuan, HsuSung-Kon, KimHuanyu, ChengSung-Uk, ZhangHuayang, YuSung-Won, YooHubert J. M., GeijselaersSungWoo, SohnHubert, AnyszSungwook, ChoungHubert, JopekSunil Kumar, RamamoorthyHubert, RahierSunil, K. SharmaHubertus, HintzenSunney, ChanHudisteanu, IulianaSunniva, CollinsHueng-Chuen, FanSunwoo, LeeHugh, LeeSuong V., HoaHugo M. R. D., SilvaSuraj, JoshiHugo M., OliveiraSurendra Krushna, ShindeHugo P., Van LandeghemSuresh Kannan, BalasingamHugo, CostaSusan, SelkeHugo, SalmonSusana R., SousaHui, LiSusana, SérioHui, WuSusanna, KällbomHui, YanSusanna, PilusoHui, YaoSusanne, MichelicHui, ZhangSuset, Barroso-SolaresHui, ZhengSushanta, GhoshalHuijing, HeSusumu, HamaHuijing, ZhaoSusumu, OnakaHuijun, LiSuvash Chandra, PaulHui-Shen, ShenSuvi, Santa-ahoHuizhi, ChenSuzan, MeijsHumberto, Almeida JrSuzue, YonedaHung Ji, HuangSvatopluk, ZemanHung-Chi, ChengSven, SängerlaubHungJi, HuangSvetlana V., KononovaHunter Bryan, HendersonSvetlana, DerkatchHuolin, HuangSvetlana, KrylovaHusain, ShekhaniSvetlana, PolivtsevaHusam, HusseinSvetlana, PushkarHusanu, ElenaSvetlana, Von GratowskiHussain, AltammarSvetoslav, KolevHussien, HegabSvetozar, MusićHuu Kien, BuiSwadeshmukul, SantraHwai-Chung, WuSwarup, RoyHwa-Jung, KimSwee Leong, SingHwan Soo, DowSwetha, ChandrasekaranHwi-Dong, JungSyed HaiderHyeok, KimSyed Saad B., QasimHyeonggon, KangSyeda Adila, AfghanHyeongjoo, KimSylvain, AchelleHyeong-Ki, KimSylvain, BertainaHyeong-Yeol, KimSylvain, DancetteHyeon-Jong, HwangSylvain, DantoHynek, BalcarSylvain, MagneHyo Jung, KimSylvain, MartinHyo, KangSylvain, QueyreauHyoung-Su, HanSylvie, Dabos-SeignonHyun Do, YunSylwester, SamborskiHyun, KimSylwia, ChudyHyun, SeokSylwia, MoziaHyung D., BaeSylwia, Zielińska-RaczyńskaHyungjun, SongSyyed Adnan Raheel, ShahHyungwoo, KimSzabó Ferenc, JánosHyunjo, JeongSzabó Péter, JánosHyunsung, KimSzabolcs, FischerHyu-Soung, ShinSzende, TonkI.-Chen, ChenSzetsen, LeeIan, MaclarenSzilvia, KlebertIan, MajorSzu-Hung, ChenIan, MartinsSzymon, BanaszakIan, McCueSzymon, ChorazyIan, PotterSzymon, DawczynskiIan, SmithSzymon, ŁośIan, WoodheadSzymon, ManiaIan, WymanSzymon, WojciechowskiIbrahim Fatih, CengizSzymon, ZelewskiIbrahim M. A., ElSherbinyTadanobu, InoueIbrahim M., GadalaTadas, DambrauskasIbrahim, ElgaliTadashi, KawaiIbrahim, ShaabanTadeja, KosecIchiro, MinamiTadeusz M., MuziołIchiro, TerasakiTadeusz, HryniewiczIckhyun, SongTadeusz, ŁagodaIda, WestermannTadeusz, MuziolIeva, MisiunaiteTadeusz, SiwiecIeva, PlikusieneTadeusz, SzymczakIgnacio Martín, NietoTae J., ParkIgnacio, PuertasTae Whan, HongIgnazio, CannasTaegyu, LeeIgnazio, RoppoloTaeho, AhnIgor I., GorbachevTaehoon, KimIgor S., SrotinTae-Hyun, KimIgor S., YasnikovTae-Hyung, KimIgor S., ZhirkovTaejong, PaikIgor V., KhudyakovTaek Yong, HwangIgor, BobrovskijTaek, LeeIgor, BorovikTaekeun, OhIgor, DubenkoTaeyoub, KimIgor, GolubTae-Yub, KwonIgor, IatsunskyiTaffese, WoubishetIgor, MakarovTahir, RasheedIgor, MazurTahira, PirzadaIgor, MedvedTa-I., YangIgor, MedveďTai-Cheng, ChenIgor, MoravcikTai-Feng, HungIgor, MuchaTaiha, JooIgor, PolozovTaiho, KambeIgor, RubtsovTairong, KuangIgor, SevostianovTaishi, KobayashiIgor, ShardakovTaisiya, SukhikhIgor, ShchetininTaito, MiuraIgor, ShishkovskyTakaaki, ArahiraIgor, ŠtubňaTakaaki, KawamuraIgor, VelkavrhTakaaki, WajimaIikka, VirkkunenTakafumi, KitazawaIkeo, NaokoTakahiro, GunjiIL Tae, KimTakahiro, KannoIL, SohnTakahiro, KunimineIlare, BordeaşuTakahiro, SekiIlaria, CacciottiTakahisa, ArimaIlaria, CapassoTakashi, IizukaIlaria, GiustiTakashi, KajitaniIlbey, KarakurtTakashi, MatsuoIldiko, BadeaTakashi, NagoshiIldiko, PeterTakashi, NakamotoIleana Denisa, NistorTakashi, OkijiIleana Nicoleta, PopescuTakashi, YanagishitaIleana, FloreaTakashiro, AkitsuIleana, RauTakayuki, HasegawaIlenia, FarinaTakayuki, OhtaIlhwan, ParkTakayuki, ShiraiwaIlia, RasskazovTakayuki, TakasugiIlian, HristovTakayuki, TsukegiIliana, FauziTakehiko, GotohIlias, EfthimiopoulosTakeo, OhsawaIlioara, OnigaTakeshi, FukudaIliya, PetrievTakeshi, HagioIlja, GunkelTakeshi, NagaiIlka, SchmueserTakeshi, NagaseIlker, BayerTakeshi, OkuzonoIlkeun, LeeTakeshi, UemoriIlko, BaldTakeshi, YabutsukaIlona, WandzikTakeshi, YamaguchiIlutiu-Varvara, Dana AdrianaTakuichi, SatoIlya G., ShenderovichTakuma, KurehaIlya, OkulovTakuma, ShibataIlya, UlasovTakumi, AsakuraIma, GhaeliTakumi, ShimomuraIman, AbdallahTakuo, SakonIman, SalehiniaTakuro, HosomiIman, Sari SarrafTakuya, HayashiIman, YazdiTakuya, IshimotoImane, BelyamaniTakuya, TaniguchiImants, KaldreTal, TamariImre, BakonyiTalal, MaksoudImre, CzinegeTalib, Al-AmeriImre, OrbulovTamako, HataImyhamy M., DharmadasaTamara Aleksandrov, FabijanićIn Woo, CheongTamara, TomašegovićIñaki, GarmendiaTamas, KomivesIndrek, MustTamas, OroszInes, Flores-ColenTamás, TörökInês, MatosTamas, VargaInese, FilipovaTamer, BreakahIngars, ReinholdsTamerlan T., MagkoevInge, HoffTamitake, ItohInge, LindemannTammana S. R. C., MurthyIngo, HertrichTan, SuiIng-Song, YuTancu, Ana Maria CristinaIngyu, LeeTânia, RosadoInho, NamTanja, LubeInna, KurganskayaTanja, PessiInna, LisnevskayaTanmoy, MukhopadhyayIn-Sang, YangTanmoy, PramanikIn-Seok, SongTanveer, AhmadIn-Seok, YoonTanveer, TabishInsoo, RoTanvir, ManzurInsun, YoonTanvir, QureshiIn-Sung, YeoTao, HongIoan Călin, RoşcaTao, LiIoan Cezar, MarcuTao, LuIoan, AschileanTao, MengIoan, MamaligaTao, ShaoIoan, StamatinTao, SunIoan, UrsuTao, WangIoana Maria, CorteaTao, XuIoana Roxana, BordeaTao, YuanIoana, ChiulanTao, ZhangIoana, CsakiTao, ZhouIoana, DemetrescuTao-Hsing, ChenIoana, GhiutaTa-Peng, ChangIoana, MaiorTar, IldikóIoana, StanculescuTarak, Ben ZinebIoanina, ParlatescuTaras, ParashchukIoanna, DeligkioziTarek, AllamIoannis A., AsimakopoulosTarek, BachaghaIoannis K., KarabagiasTarek, El-BialyIoannis, AnastopoulosTarek, RagabIoannis, BratsosTariq, NazirIoannis, KoutelidakisTarkes Dora, PallicityIoannis, KoutselasTarlan, HajilouIoannis, MastorakosTarmo, TammIoannis, NikolakakisTaro, UraseIoannis, PetsagkourakisTaron, MakaryanIoannis, SamarasTarun, GoswamiIoannis, SpanopoulosTatheer, ZahraIoannis, TsivintzelisTatiana E., ItinaIoannis, YentekakisTatiana N., MyasoedovaIolanda, FrancoliniTatiana S., DeminaIon Octavian, PopTatiana S., FrolovaIon-Dragos, UtuTatiana, AndreaniIonela, Carazeanu PopoviciTatiana, DeminaIonel-Alexandru, GalTatiana, LatychevskaiaIonelia, VoiculescuTatiana, PyatinaIonuţ Claudiu, RoatǎTatiana, ZolotoukhinaIonuţ, LedeţiTatjana, DedovaIonut-Ovidiu, TomaTatjana, Glaskova-KuzminaIraida, KolcunováTatjana, GricIraklii I., EbralidzeTatsiana, MikulchykIrati, BarandiaranTatsuo, HasegawaIren E., KuznetsovaTatsuro, MoriIrena M., HlaváčováTatsuya, KikuchiIrena, GlazarTatu, PinomaaIrena, GlažarTatyana, IvanovaIrena, Jacukowicz-SobalaTatyana, KoutzarovaIrena, Jankowska-SumaraTawfik, KhattabIrena, MamajanovTea, MarohnićIrena, PaulinTedeusz, SałacińskiIrena, ŽmakTeen Hang, MeenIrene Del Sol, IllanaTei-Chen, ChenIrene, AndreuTej, LamichhaneIrene, BonadiesTeng-Chun, YangIrene, Buj-CorralTengfei, FuIrene, VillaTeng-Shih, ShihIreneusz, KapustaTeng-Wen, ChangIreneusz, PiwońskiTeodor, MilenovIreneusz, SzachogluchowiczTeodora Stoyanova, LyubenovaIreneusz, ZagórskiTeodora, StaicuIrez, Alaeddin BurakTeofil, JesionowskiIrfan Ahmad, KhanTerence A., ImberyIrimpan, MathewsTeresa Leonor, Martins MorgadoIrina A., KurzinaTeresa Moura E., SilvaIrina A., ZverevaTeresa, BravoIrina D., YushinaTeresa, Cotes-PalominoIrina Domnica, TitorencuTeresa, FascianaIrina Mikhailovna, MakeevaTeresa, GurayaIrina S., EdelmanTeresa, Pineda RodríguezIrina Ya, MittovaTeresa, RucinskaIrina, AtkinsonTeresa, RussoIrina, GoryachevaTeresa, StryszewskaIrina, IelciuTeresa, ZychIrina, LegchenkovaTereza, PavlůIrina, LoginovaTero, LuukkonenIrina, LyanguzovaTero, TuovinenIrina, PetreskaTerry R., McnelleyIrina, SeverinTerttu, HukkaIrina, SmolinaTessy, Theres BabyIrina, SulaevaTetiana, ManykIrina, ZgurăTetsumi, IrieIrina, ZhevstovskikhTetsuo, ShojiIrmgard, WeißensteinerTetsuo, YamaguchiIrshad, KammakakamTetsuya, KakoIryna, GolovinaTetsuya, KanagawaIsaac, JacobTetsuya, NakagawaIsaac, Torres-DiazTh. N., NikolaidisIsabel María, Martínez SierraThami, ZeghloulIsabel Pestana, Paixão CansadoThanasis D., PapathanasiouIsabel, CoelhosoThanh-Canh, HuynhIsabel, DuarteThemis, MatsoukasIsabel, Montealegre-MeléndezThemistoklis, KabanosIsabel, Rial-HermidaTheo, CalaisIsabel, RucandioTheocharis, BaxavanisIsabelle, HuynenTheodora, Krasia-ChristoforouIslam, MosaTheodoros, RousakisIslam, ShyhaThibaut, SoulestinIsmael, MatinoThierry, ChartierIsmael, PellejeroThierry, RibeiroIsmail, FidanThiyagarajan Jothi, SaravananIsnaldi, Souza FilhoThomaida, PolydorouIsrael, FelnerThomas F., GeorgeIsrafil, KucukThomas J., TaylorIstrate, BogdanThomas M., GruppIstvan, PalinkoThomas M., JohnsonIstvan, SzilágyiThomas N., FarrisIta, JunkarThomas N., MasseyIt-Meng, LowThomas Walter, CorneliusItsuki, MiyazatoThomas, AllenItthipon, JeerapanThomas, AntretterItxaso, CalafelThomas, AuzelleItzhak, OrionThomas, ChristopoulosIulian, AntoniacThomas, DechatIulian, PanaThomas, DippongIuliana, CotaThomas, DorinIuliana, MotrescuThomas, EchterhofIuliia, MorozovaThomas, FiedlerIuri B. C. M., RochaThomas, GerekeIvan A., ParinovThomas, GkourmpisIvan Nikolaevich, ErdakovThomas, GoudoulasIvan S., RaginovThomas, IskratschIvan S., ZhidkovThomas, KoneggerIvan V., SmirnovThomas, LampkeIvan Yu., SkvortsovThomas, LindnerIvan, AnželThomas, MachonIvan, BassaniniThomas, MayerIvan, BelyaevThomas, Mayer-GallIvan, Cabrera I. FaustoThomas, MichlIvan, ChenchevThomas, PatersonIvan, ChodakThomas, SchmidIvan, GalvãoThomas, SchnabelIvan, GiorgioThomas, SchweizerIvan, HollýThomas, SiegmundIvan, KozyatnykThomas, StergiopoulosIvan, KrausThomas, ThiebaultIvan, LesovThomas, WaltherIvan, LomakinThor, BechsgaardIvan, NemecThorsten, KoslowskiIvan, PetryshynetsThresia, ThomasIvan, RužiakThurid, GspannIvan, ShorstkiiTia, MangIvan, ShtepliukTiago A., FernandesIvan, TarasovTiago A., RodriguesIvana, BarisicTian, LiangIvana, GrčićTianfeng, YuanIvana, IvanićTianfu, GuoIvana, MilicevicTianhao, WangIvanka Netinger, GrubešaTian-Li, WuIvano, AlessandriTianrui, ZhaiIvaylo, DimitrovTianyou, TaoIveta, MarkováTianyu, LiuIvica, BokoTianyu, XieIvica, GarašićTianzhu, SunIvica, KožarTianzhu, ZhangIvo, ČernýTibor, BedoIvo, SchindlerTibor, IžákIvo, StachivTibor, KrenickyIvona, NuićTibor, KvačkajIvor, LoncaricTibor, PasinszkiI-Wen Peter, ChenTiejiong, LouIwona, KarbownikTien Duc, PhamIwona, KwiecieńTiffany, PellizzeriIyas, KhaderTihomir, TomašičIzabela, Barszczewska-RybarekTiina, NypeloIzabela, HagerTiina, TitmaIzabela, JendrzejewskaTill, FrömlingIzabela, Kalemba-RecTill, LeissnerIzabela, MajorTill, ValléeIzabela, MatułaTilo, ZienertIzabela, MiturskaTim C., HolgateIzabella, GrzegoryTim, GrunwaldIzhak, EtsionTim, PeppelIzumi, NakamuraTimo, SajavaaraIzumi, TaniguchiTimothy C., EiseleJ. Barry, WiskelTimothy Robert, MerrittJaber Rezaei, MianroodiTimothy, HunterJacek A., MichalskiTimothy, TrusterJacek, AndrzejewskiTimur Sh., AtabaevJacek, FilipeckiTimur, SalievJacek, GołaszewskiTimur, YildirimJacek, GóraTina, KeglJacek, GórkaTing, TsuiJacek, JaniszewskiTing-Yu, LiuJacek, KaczmarTingyun, WangJacek, KatzerTino, GottschallJacek, KrawczykTitan, PaulJacek, LubczakTitu, Mihail AurelJacek, MatysTitus A., JennyJacek, MuchaTitus, SanduJacek, PietraszekTiuc Ancuţa, ElenaJacek, RylTiziana, FuocoJacek, SawickiTkachenko, VolodymyrJacek, SiódmiakTkacz, MarekJacek, ŚliwińskiTkhe Kyong, FamJacek, SzczerbaToan, DinhJacek, SzulejTobias, AbtJacek, TomkówTobias, BrinkJacek, WojnarowiczTobias, FeyJacek, WychowaniecTobias, MüllerJack, HaleyTobias, TauböckJack, McAlorumTodor, VuchkovJack, SkinnerTohru, HayakawaJacob, HeltzelTom, HeitmannJacob, HochhalterTom, KampermanJacob, HorwitzTomáš, DvorskýJacob, OlchowkaTomas, KalinaJacopo, TirilloTomáš, KrumlJacqueline, KrimTomas, ProsekJacques C., VédrineTomáš, ProšekJacques, GilloteauxTomas, SimurdaJacques, LamonTomasz M., KarpinskiJacques, LengTomasz M., KarpińskiJadwiga, FangratTomasz Norbert, KołtunowiczJadwiga, Wojkowska-MachTomasz P., OlejnikJae Bok, SeolTomasz, BartkowiakJae Hong, KimTomasz, BłaszczyńskiJae Hoon, ChoiTomasz, BulzakJae Hyuk, LimTomasz, ChmielewskiJae Sung, KwonTomasz, CyganJae Sung, LeeTomasz, CzeppeJae Wung, BaeTomasz, CzujkoJaehan, JungTomasz, DrzymałaJae-Han, LimTomasz, DurejkoJaeheum, YeonTomasz, DylJae-Hong, KimTomasz, GarbowskiJae-Hoon, SimTomasz, GawełJaehwang, LeeTomasz, Goetzendorf-GrabowskiJaehyun, LeeTomasz, GorzelańczykJae-Hyun, LeeTomasz, JaroszJae-Hyung, ChoTomasz, KaniaJaejun, LeeTomasz, KikJae-Kyu, YangTomasz, KoczorowskiJaemin, KongTomasz, KowalukJae-Sang, ParkTomasz, KoziorJae-Suk, RyouTomasz, KrystofiakJae-Won, ChoiTomasz, KubiakJaewoo, KimTomasz, KurzynowskiJaeyun, MoonTomasz, LipińskiJagadeesh K., VenkatesanTomasz, MajewskiJagannath, PadmanabhanTomasz, MościckiJagoda, LitowczenkoTomasz, MuszyńskiJai, SinghTomasz, PiotrowskiJaime Bonnín, RocaTomasz, PlocinskiJaime Pedro Oliveira, Da SilvaTomasz, PonikiewskiJaime, Fernandez-BacaTomasz, PytlowanyJaime, PunaTomasz, RozwadowskiJaime, Ramirez-VickTomasz, RudnickiJaime, ViñaTomasz, RydzkowskiJainagesh, SekharTomasz, SiwowskiJaipal, GuptaTomasz, ŚlebodaJaka, BurjaTomasz, ŚlęzakJakapan, ChantanaTomasz, SochaJakob, BuchheimTomasz, StrękJakob, HeierTomasz, SzymborskiJakob, KübarseppTomasz, SzymczakJakob, WohlertTomasz, TańskiJakub W., NarojczykTomasz, TomaszewskiJakub, AdamekTomasz, TrzepiecinskiJakub, DuszczykTomasz, WasilewskiJakub, HlostaTomasz, WróbelJakub, HodulTomasz, ZdebJakub, KonkolTomasz, ŻelazińskiJakub, MatusikTomaz, KekJakub, ZdartaTomaz, PepelnjakJalal, IsaadTomče, RunčevskiJamal, KhatibTomi T., LiJamal, NasirTomi, SuhonenJames Bernard, MetsonTomica, HrenarJames E., KrzanowskiTomislav, KišičekJames E., ToneyTomislav, PintauerJames G., RavinTommaso, CastroflorioJames G., RyanTommaso, D’AntinoJames H., CarterTommaso, LomonacoJames Kit-hon, TsoiTomoaki, SatomiJames P., ConnollyTomohiko, HojoJames, CahillTomohiro, TojoJames, FurnessTomohisa, KumagaiJames, HannaTomokazu, HattoriJames, LimTomoko, AkaiJames, MollerTomoko, SugimotoJames, NovakTomoya, NishiwakiJames, ParkerTomoya, TakadaJames, RoscowTomoyuki, FujiiJames, SherwoodTomoyuki, MiyamotoJames, YangTomoyuki, TakuraJames, ZubackTomoyuki, TodaJamieson, BrechtlTon, LubrechtJamila, BoudadenTong, FeiJan Erik, LindqvistTong, GaoJan K., ZarebaTong, LiuJan, BiernatTongyan, PanJan, BogackiTonica, BončinaJan, BohlenTonino, TrainiJan, BrodaTonya B., WolfeJan, ČapekToralf, BeitzJan, DecTorben, JensenJan, DzuganTore, KvandeJan, FabryTorgom K., AkopyanJan, FořtTorsten, FritzJan, GóreckiToru, KanbayashiJan, GrymToru, SasakiJan, HonzíčekToru, UtsunomiyaJan, HošekToshifumi, SatohJan, HuebnerToshiharu, KazamaJan, IlavskyToshihide, IgariJan, JezierskiToshiki, MiyazakiJan, KaziorToshio, NaitoJán, KizekToshio, OgawaJan, KlusakToshiro, TomidaJan, KoplíkToshiyuki, ItohJan, KotekToshiyuki, KanakuboJan, MacakToshiyuki, KawaiJan, MajerníkToyanath, JoshiJán, MoravecTracie L., FerreiraJan, MrazekTraian, DumitricaJan, NecasTran Quyet, ThangJan, NiederwestbergTravis W., WalkerJan, PaczesnyTrent, AshtonJan, PapugaTretsiakova-McNally, SvetlanaJan, PizońTrevor C., BrownJan, SchmidtTrevor D., RapsonJán, SedliačikTrevor, AbbottJan, SuchaniczTrevor, BlackburnJan, SuchorzewskiTri Ho Minh, LeJan, ŚwierczekTri-Dung, NgoJan, SýkoraTristan, PetitJan, TomastikTroy Y., AnsellJan, TrejbalTruong, VoJan, ValentinTs. D., DikovaJan, ValičekTse-Min, LeeJán, ViňášTsu-Chuan, LeeJan, WajsTsunehisa, MikiJan, ZawalaTsung Pin, HungJan, ZidekTsung Sheng, KaoJana, BidulskáTsung-Hsien, ChuangJana, HolubováTsutomu, ItoJana, HorakovaTsuyoshi, ShimoJana, PiskTudor, PetrinaJana, ŠmilauerováTuhin, MukherjeeJanak, SapkotaTúlio, HonórioJane Ru, ChoiTullio, GenovaJanez, PerkoTullio, MonettaJanez, ZavašnikTuncer, EdilJan-Henrik, SmåttTünde, KovácsJanina, AdamusTung, PhanJanja, StergarTungky, SubrotoJanja, TrcekTung-Yuan, YungJanka, VaškováTuong, LeJanne, JuoksukangasTutunaru, BogdanJanne, KoivistoTyurin Yury, IvanovichJanne, PesonenTze-Bin, SongJannik, LarsenTz-Feng, LinJannis N., LemkeTzong-shi, LiuJannis, WenkTzu-En, LinJanno, ToropTzu-Sen, YangJános, DobránszkyTzu-Yen, HuangJanos, LukacsUbong, EduokJanos, MizseiUday, SinghJanos, SzepvolgyiUdayabhanu, JammalamadakaJanusz, BrolUdishnu, SanyalJanusz, DabrowskiUlf, BetkeJanusz, DubowikUli, LohbauerJanusz, KluczyńskiUlises, AmadorJanusz, KonkolUllrich, ScherfJanusz, KonstantyUllrich, SteinerJanusz, KozakiewiczUlrich D., JentschuraJanusz, KrawczykUlrich, BaischJanusz, LelitoUlrich, BaumgartnerJanusz, NowickiUlrich, PontJanusz, SikoraUlrike, SteinmannJanusz, SmulkoUmar, ShafiqueJanusz, TomczakUmberto Capasso, PalmieroJanusz, TorzewskiUmberto, CelanoJanusz, WójcikUmberto, PriscoJanusz, ZmywaczykUmberto, RaucciJari, RuuskaUmmul Khair, SultanaJari, TuominenUmut, CakmakJarmila, VilcakovaUnbong, BaekJaromír, DrápalaUpulitha Eranka, IllangakoonJaromír, KopečekUri, RavivJaromír, MoravecUrmas, JohansonJaroslav, BrunckoUrška, Vrabič BrodnjakJaroslav, ČechUrszula, BazylińskaJaroslav, FilipUrszula, FilipkowskaJaroslav, FojtUrszula, MizerskaJaroslav, HornakUrszula, StachewiczJaroslav, JerzUsman, AliJaroslav, KousalUtz, Von WagnerJaroslav, KováčUwe, StuhrJaroslav, KovacikVaclav, BabuskaJaroslav, KruisVáclav, HolýJaroslav, LegemzaVaclav, PaidarJaroslav, MenčíkVaclav, PrajzlerJaroslaw Jan, JasinskiVáclav, RancJaroslaw W., KlosVaclav, SeberaJarosław, BąkowskiVadim P., BoyarskiyJarosław, BartnickiVadim V., PotapovJaroslaw, BieniasVadim, KhlestkinJaroslaw, GalkiewiczVadim, MizonovJarosław, GórskiVagelis, HarmandarisJarosław, GórszczykVagelis, KaroutsosJaroslaw, JacakVahid, FarzanehJarosław, JakubowiczVahid, SerpooshanJarosław, KaczmarczykVaibhav, VibhuJarosław, KaszewskiVaishak, ViswanathanJaroslaw, KozubaValentin Ştefan, OleksikJarosław, LatalskiValentin, BrudnyiJarosław, MarciszValentin, IoniţǎJarosław, WozniakValentin, JmerikJarosław, ŻmudzkiValentin, LeroyJarosław, ZubrzyckiValentin, MateevJasmin, JelovicaValentina, BeghettoJasmine, SinhaValentina, BertanaJason, BrenkerValentina, ButvinaJason, HoffmanValentina, CaudaJason, LimValentina, CeteanJason, ScottValentina, CollaJasper Rikkert, PlaisierValentina, DincaJavad, MehrmashhadiValentina, DomeniciJavad, RazaviValentina, FurlanJavad, TavakoliValentina, GargiuloJavaid, ButtValentina, MameliJavier E., Contreras-ReyesValentina, MarascuJavier Fernando, Jimenez AlonsoValentina, MazzantiJavier Martínez, TorresValentina, PernaJavier Rodríguez, Vázquez De AldanaValentina, SabatiniJavier S., Blázquez GámezValentina, SessiniJavier, AldazabalValentina, SimaginaJavier, CañavateValentina, UivarosiJavier, CarballoValentina, VasilevskayaJavier, Fernandez-GarciaValentina, VenutiJavier, Garcia-PardoValentyn, MaidannykJavier, GilValeria, De MatteisJavier, IllescasValeria, MertingerJavier, Martinez RodrigoValeria, VignaliJavier, Martín-SánchezValérie, MontouilloutJavier, NarcisoValérie, Paul-BoncourJavier, PadillaValerio Flavio, GiliJavier, PazValerio, De BiagiJavier, Pereiro-BarcelóValerio, PiniJavier, RiberaValeriy I., BogdanovichJavier, SánchezValeriy, DudkoJavier, SeravalliValery Stanislavovich, LesovikJavier, Xavier GilValery, KulichikhinJayachandra Hari, MangalaraValery, PutlayevJayachandran, VenkatesanValery, ShabashovJayanthi, KumarValter, CarvelliJayne, GarnoVamsi K., YadavalliJayraj, VaghasiyaVamsi, BorraJea Uk, LeeVan-Canh, TongJean Aime, MbeyVance G., NielsenJean Christian, BernèdeVanessa Paredes, GallardoJean M., PelletierVanira, TrifilettiJean Marc, TullianiVanja, KokolJean, DumoulinVan-Tho, HoangJean, MencikVarun, ChaudharyJean-Claude, BunzliVarun, VohraJean-Claude, GrivelVarvara, GiuseppeJean-Denis, ParisseVarvara, ShubinaJeanette, OrlowskyVarvara, SygouniJean-François, BlaisVasil, GeorgievJean-François, DrilletVasil, SimeonovJean-Luc, FiheyVasilica, PopescuJean-Michel, BechtVasiliki, KamperidouJean-Michel, LebanVasilios C., KapsalisJean-Michel, RomanoVasilis, KanakoudisJeannette, YenVasilis, SarhosisJean-Noël, JaubertVasily, EfremenkoJean-Philippe, BerteauVassilis, InglezakisJean-Pierre, BiratVassilis, KapsalisJean-Pierre, DalmontVassilis, StathopoulosJean-René, HamonVasyl, ShvalyaJean-Sébastien, BaumannVattikuti S. V., PrabhakarJean-Yves, DubozVeerappan, ManiJedo, KimVeerasikku Gopal, DeepaganJeeyoung, YooVeerendra, AtlaJeff C. W., WangVegar, OttesenJeff, HughesVeikko, LinkoJeffrey A., HawkVelram Balaji, MohanJeffrey B., AllenVenanzio, GiannellaJeffrey D., EinkaufVenelinov, TonyJeffrey G., LundinVenkat, PadmanabhanJeffrey M., TingVenkata Karthik, NadimpalliJeffrey, De VeroVenkatesh, VijayaraghavanJeffrey, GimbleVenkatesham, TallapallyJeffrey, HawkVera I., IsaevaJeffrey, VenezuelaVera L. M., SilvaJehan, El-JawhariVera, MarinovaJehwang, RyuVera, RealinhoJelena, ČulinVerena, LeitgebJelena, DragašVerónica, CalderónJelena, Rnjak-KovacinaVerónica, García-SanzJelena, Srnec NovakVeronica, GrayJelena, VasiljevićVerónica, Montes GarcíaJenica, PaceagiuVerónica, Montes-GarcíaJennifer A., Ciezak-JenkinsVeronika, HuntosovaJennifer H., ShepherdVeronika, MazanovaJennifer, GottfriedVeronika, TunakovaJennifer, HamptonVeronique, DoquetJennifer, HulingVesa, SaikkoJennifer, PattersonVesna, AlarJenő, GubiczaVesna, ZalarJen-Ray, ChangViacheslav, BalobanovJens, HoltmannspötterViacheslav, BazhenovJens, PetersViacheslav, KuksenkoJeong Gook, JangVibhore Kumar, RastogiJeong Jae, WieVicente I., ÁguedaJeong, YeumVicente, AlberoJeongguk, KimVicente, Faus-MatosesJeonghoon, LeeVicente, Flores-AlésJeongsoo, NamVicente, RivesJeongyong, KimVictor Dmitrievich, LakhnoJerard, GordonVíctor F., VázquezJeremias, HeyVíctor Jesús, Gómez HernándezJeremy, EppVictor M., PridaJeremy, GoldmanVíctor Manuel, García SuárezJeremy, TeoVíctor Manuel, Ortiz MartínezJernej, KlemencVíctor Manuel, Pérez PuyanaJerzy J., LangerVíctor Revilla, CuestaJerzy, BobińskiVictor V., TcherdyntsevJerzy, BochniaVictor, AchaJerzy, ChlistunoffVictor, AtuchinJerzy, GorausVíctor, CárdenesJerzy, HołaVictor, EremeyevJerzy, ŁabanowskiVictor, FanelliJerzy, MalachowskiVictor, GeantăJerzy, MorgielVictor, KagalovskyJerzy, NapiórkowskiVictor, KomarovJerzy, NiagajVictor, MamaneJerzy, PodgórskiVíctor, OestreicherJerzy, SękVictor, RalchenkoJerzy, SmardzewskiVictor, RizovJerzy, SokolnickiVictor, SelinJerzy, SzyszkaVictor, ShapovalovJerzy, WawrzeńczykVictor, SoltamovJerzy, WinczekVictor, StarovJessica, AndrioloVíctor, YepesJesús C., HernandezVictoria, CorregidorJesus Lozano, SánchezVictoria, KurushinaJesús Miguel, Chacón MuñozVictoria, PiunovaJesus, CintasVictorien, ProtJesús, Del ValVictorio, CadiernoJesús, Eiras FernándezVictor-Vlad, CostanJesus, GalanVidya, KishoreJesús, Hernández SazViera, HomolovaJesús, LemusViera, ZatkalíkováJesús, López-SánchezViet-Duc, LeJesús, Mena-ÁlvarezVigilio, FontanariJesús, Mínguez-AlgarraVijay K., TomerJesús, Prado-GonjalVijay, Kumar ThakurJesús, Suárez GonzálezVijay, SukumaranJesús, ToribioVijayabhaskar, VeeravalliJesús-María, García-MartínezVijayavenkataraman, SanjairajJia, LinVijaykumar B., VarmaJia-Bao, YanVijaykumar, MarakattiJiabin, LiVikas, PatelJia-Chong, DuVi-Khanh, TruongJiajing, ZhouVikram, BedekarJialei, LiuVikram, DeyJialiang, XuVikram, PakrashiJialong, ShenViktor P., AstakhovJiamin, SunViktor, GribniakJiamin, WuViktor, HlavičkaJian, CaoViktor, KharinJian, LiViktor, KudiiarovJian, LinViktor, MolnárJian, OuyangViktor, NormanJian, PengViktor, SchlosserJian, WangViktor, TothJian, XuViktoria E., MartínJian, YuViktoria K., KisJianchao, CaiViktória Kovács, KisJiang, ChengVila Santos, MariaJiang, GuoVilem, BartunekJiangfeng, NiVilko, MandićJiangkun, DaiVinaya K. B., KumarJianhua, ZhaoVinayak, KaushalJianhua, ZhouVincent E., LambertiJianjun, WangVincent, BaltazartJianmin, MaVincent, FridricJianmin, YangVincent, HumblotJianming, WenVincent, JiJian-qiang, WangVincent, LaudeJianquan, LuoVincenza, BrancatoJianwen, PanVincenzina La, SpinaJian-Wen, WangVincenzo M., SglavoJianxun, DingVincenzo Manuel, MarzulloJiao Jiao, LiVincenzo Mariano, MastronardiJiaqi, LiVincenzo, BellantoneJiaqi, XuVincenzo, BrunoJiaqing, WangVincenzo, CaraccioloJiaqing, XiongVincenzo, CostanzoJiarui, HeVincenzo, D’antoJiawei, GongVincenzo, GrassiaJiawei, XiangVincenzo, GuarinoJiaxi, XuVincenzo, LoschiavoJiayang, WuVincenzo, MallardoJiayi, YuVincenzo, MoramarcoJia-Zhuang, XuVincenzo, PalmaJiba, DahalVincenzo, StornelliJibran, KhaliqVincenzo, TebaldoJichao, LiVincenzo, VaianoJie, BaoVineet, KumarJie, YangVineet, ShahJie, ZhouVinh Tung, LeJiehua, LiVinu, KrishnanJifeng, GaoVioleta, Bokan-BosiljkovJiheng, LiVioleta, DediuJihoon, KimVioleta, MelinteJihui, YuanVioleta, NiculescuJihyun, BaeVioleta, PurcarJijin, XuViorel, CircuJill, Steinbach-RankinsViorel, GoantaJim, WoodhouseViorel, PaleuJi-Man, ParkViorel, PaunoiuJimbert, PelloViorel, SanduJimin, KwonViorel-Aurel, SerbanJin Hyun, LeeViorel-Puiu, PaunJin Man, KimVipuil, KishoreJin Woo, LeeVipul, PatelJin Yong, ParkVirgil, TeodorJin, GengVirgil-Florin, DumaJin, GongVirginia, BrancatoJin, MotoyanagiVirginie, DumasJin-Cherng, HsuVirginija, JankauskaiteJinfeng, JiaVirginijus, BukauskasJing, ChenVisan, Anita IoanaJing, DuVishnu, SaseendranJing, HanViswanathan, SwaminathanJing, JinVit, KřivýJingan, LiVit, ProchazkaJingang, YuVitali, SikirzhytskiJingfeng, HuangVitalii, ShtenderJinghui, WangVitalis, BriedisJingjie, HuVitaly A., MorozovJingjie, YeoVitaly S., SokolovskyJingJing, ZhangVitaly, BuckinJingkun, LiVitaly, TseluikinJingming, CaiVitaly, ZviaginJing-Shan, DoVítezslav, KrivanekJingwei, HeVítězslav, MášaJingxian, YuVito, DianaJingyao, SunVito, GiganteJing-Yun, WuVito, PagliaruloJinhan, MoVito, RicottaJinhua, SunVito, RizziJinjin, HaVitor M., CorreloJinkui, FengVitor, CarneiroJinlian, HuVittorio, ChecchiJinming, HuVittorio, NicolosiJinming, LiuVivek, DhandJinqiang, NingVivek, PatelJin-Soo, AhnVivi, AnggrainiJinyan, WangVivi, TornariJinyang, XuViviana P., RibeiroJin-Yeon, KimVjaceslavs, LapkovskisJin-Yoo, SuhVladan, KoncarJin-Young, LeeVladimir A., AseevJin-yuan, QianVladimir A., EsinJiranuwat, SapudomVladimir A., SkripnyakJiri, BarekVladimir B., ShirokovJiří, BrožovskýVladimir G., PetrovJiří, CzernekVladimir K., IvanovJiri, DuchoslavVladimir L., TassevJiri, KlemesVladimir M., SegalJiří, KubásekVladimir S., PrudkovskiyJiří, MatejíčekVladimir V., AndreevJiří, PazderkaVladimir V., KuznetsovJiri, PflegerVladimir, BalabanovJiri, SvobodaVladimir, CechJiro, KitagawaVladimír, ChmelkoJi-Sang, ParkVladimir, EgorkinJitendra Kumar, SinghVladimir, FedorovJitendra, SinghVladimír, KopeckýJithender J., TimothyVladimir, KunetsovJittisa, KetkaewVladimir, OsipovJiun-Ren, HwangVladimir, PichuginJiusheng, LiVladimir, PopovJiuxing, JiangVladimir, PromakhovJi-Won, ParkVladimir, ShvartsmanJizhou, FanVladimir, TihkonovJo, VerhaevertVladimir, VaksJoachim, BaumeisterVladimir, VinokurovJoachim, BreternitzVladimir, VishnyakovJoachim, HausmannVladimira, NovotnaJoachim, KoetzVladimíra, PetrákováJoachim, MayerVladimirs, BiziksJoachim, PaierVladislav, GurzhiyJoachim, StorsbergVladislav, KrisyukJoachim, VollbrechtVladislav, VoronenkovJoamin, Gonzalez-GutierrezVladyslav, TurloJoan Daniel, PradesVlastimil, BílekJoan Josep, Roa RoviraVlastimil, BorůvkaJoan, FormosaVlastimil, KuldaJoan, TorrasVlastimil, MatějkaJoan, Torrens-SerraVlastimil, VodarekJoana C., AntunesVoichita, BucurJoana, AlmeidaVoitech, StankevicJoana, AntunesVojko, MatkoJoana, Mesquita-GuimarãesVojtech, AdamJoana, ValenteVolf, LeshchynskyJoan-Josep, SuñolVolker Paul, SchulzJoanna Julia, SokołowskaVolker, SchulzeJoanna Maria, DulińskaVolker, VentzkeJoanna N., JadczakVolodymyr, BonJoanna, BorowiecVolodymyr, SmetanaJoanna, BrzeskaVyacheslav, KuznetsovJoanna, BzówkVytautas, BoculloJoanna, CabajVytautas, BučinskasJoanna, DepciuchVytaute, PeciulieneJoanna, DołżonekVytenis, JankauskasJoanna, FedorowiczWacław, KuśJoanna, GondroWahid, FerdousJoanna, KarbowniczekWai Yee, YeongJoanna, KolmasWail Al, ZoubiJoanna, KozłowskaWakshum M., TuchoJoanna, KujawaWaldemar, KorzeniowskiJoanna, MałeckaWaldemar, MuchaJoanna, Mastalska-PopławskaWaldemar, PerdochJoanna, MichalowskaWaldemar, PydaJoanna, MystkowskaWaldemar, ŚwiderskiJoanna, Paciorek-SadowskaWaldemar, ZiajaJoanna, PietrasikWalid, DebouchaJoanna, PisarskaWalter, CaseriJoanna, RoszakWalter, EeversJoanna, RydzWalter, LengauerJoanna, RyszkowskaWalter, StanleyJoanna, Saluk-BijakWan, ShouJoanna, Wessely-SzponderWandong, WangJoanna, WiącekWang, QunJoanna, ZdunekWang, ShunfengJoannis K., KallitsisWang-zhong, MuJoão Antônio Leal, De MirandaWanlin, ZhuJoao F., ManoWanqing, WuJoão Gomes, FerreiraWansu, ParkJoão M. C., EstêvãoWanwan, LiJoão M. P. Q., DelgadoWarda, AshrafJoão M., MirandaWarren D., HuffJoão Manuel Mendes, CaramesWaseem, AminJoão Miguel Marques, Dos SantosWataru, NorimatsuJoão P., MagrinhoWayne, NeilJoão Pedro, OliveiraWeena, LokugeJoão, Castro-GomesWei Long, NgJoão, CruchoWei, ChenJoao, PintoWei, DengJoao, PiresWei, HuJoão, RequichaWei, WangJoão, RestivoWei, WuJoão, RibeiroWei, XuJoaquim C. G., Esteves Da SilvaWei, YangJoaquim Miguel, OliveiraWei, ZhangJoaquim, BarbosaWei, ZhaoJoaquim, Minguella-CanelaWei-Chih, LaiJoaquín, Arias-PardillaWeichuan, HuangJoaquín, Coronas CeresuelaWei-Chun, LinJoaquín, Ortega CastroWei-Chyou, WeiJochen G., RaimannWeidenfeller, BerndJoe, SakaiWeidong, XiaJoe, ShapterWei-En, YuanJoel, CharrierWeifeng, LinJohan, BerglundWeihong, GuoJohan, BlomWeihua, JinJohan, HektorWei-Jen, ChangJohan, JacqueminWeijie, LiJohan, SilfwerbrandWeijie, ZhaoJohann, MogeritschWeijie, ZhouJohann, ToudertWeilin, DaiJohanna, EisenträgerWeimin, HuangJohannes H., SchmitzWei-Ting, LinJohannes, HojaWeixin, LiJohannes, HommelWeizhao, ZhangJohannes, KasnatscheewWen Chang, HuangJohannes, LützenkirchenWen, JiangJohannes, ReinerWen, ZhangJohannes, WalterWenbin, ZhouJohn Arron, StrideWencheng, JinJohn B., PariseWen-Cheng, LiaoJohn Bosco Balaguru, RayappanWen-Chin, LinJohn D., ClaytonWen-Fu, LaiJohn D., YeagerWengang, WuJohn Dominic, SmithWen-How, LanJohn F., CassidyWen-Jen, LeeJohn G., HardyWenjia, ZhangJohn Harry, CaufieldWenjie, ZhaoJohn J. S., DilipWenjihao, HuJohn K., BrooksWenjin, DingJohn T., KiwiWenjun, LuJohn W., NicholsonWenjun, ZhangJohn, AggasWenlong, ChangJohn, BartlettWenming, TongJohn, BillinghamWenqiang, ZuoJohn, BolanderWensheng, WangJohn, CampbellWentao, HeJohn, GallagherWentao, ZhangJohn, HalloranWen-Ten, KuoJohn, KechagiasWenwen, JingJohn, ManderWenxian, LiJohn, SummerscalesWenxu, ZhangJohn, TibballsWen-Ya, LeeJohn, VakrosWenyuan, CuiJohn, VenetisWenyue, LiJohn, VlachopoulosWerner, BlauJohnn P., JuddWerner, Maurer-ErtlJohnson, ZhangWerner, TheisenJoji, OkazakiWerner, WeitschiesJolanta, KowalonekWesley O., GordonJolanta, LatosińskaWieslaw A., GrabonJolanta, PaukWiesław, UrbaniakJolanta, TamošaitienėWieslawa, CiesinskaJolien, DendoovenWijitha, SenadeeraJolien, Van Der PuttenWiktor, ZierkiewiczJolita, OstrauskaiteWilfried, WunderlichJon B., SuzukiWill, LynchJon E., DahlWill, MayesJon Elvar, WallevikWilliam A., BrantleyJon Tomas, GudmundssonWilliam R., HarrellJon, GoldingWilliam R., LonghurstJonas A., PramuditaWilliam S. Y., WongJonas H., OsórioWilliam, ChuirazziJonas, JoosWilliam, HarrisonJonas, ÖrtegrenWilliam, SchonbergJonathan I., KatzWilliam, ZimmermanJonathan Mark, HallamWing Shan, ChoiJonathan, BallWiniecki, MariuszJonathan, BurnsWioletta, GrzmilJonathan, ButlerWioletta, Jackiewicz-RekJonathan, GrasmanWioletta, RaczkiewiczJonathan, LauWirginia, PilarczykJonathan, OtiWislei R., OsórioJonathan, PageWitold, BrostowJonathan, PhillipsWitold, HabratJonathan, SeyboldWitold, JakubowskiJonathan, TranWitold, OgiermanJonathon, TanksWitold, ZukowskiJong Jeong, SoonWładysław G., WieczorekJong Won, KimWojciech, BańkowskiJong Wook, RohWojciech, BorekJongbok, LeeWojciech, DawidowskiJonghoon, JooWojciech, FabianowskiJonghun, YoonWojciech, HykJonghyun, OhWojciech, JamrozikJong-Ning, AohWojciech, JurczakJong-Sook, LeeWojciech, KapłonekJongwon, YoonWojciech, KicinskiJonkwon, ChoiWojciech, KubissaJoo Seong, SohnWojciech, KujawskiJoo-Cheol, ParkWojciech, MacekJoohan, KimWojciech, NowakJoon Ho, SeoWojciech, PawlakJoon Sik, ParkWojciech, PiastaJordan, MaximovWojciech, PiekoszewskiJordan, MorelliWojciech, SmułekJordan, WeaverWojciech, StawińskiJordi, PuiggalíWojciech, StępniowskiJordi, SolerWojciech, SuderJordi-Roger, Riba RuizWojciech, TarasiukJörg, KresslerWojciech, WalachJörg, OpitzWojciech, WielebaJörg, PezoldtWojciech, ZającJorge H., MonteiroWojciech, ŻórawskiJorge Paredes, VieyraWolf-Dieter, GrimmJorge, Cara-JiménezWolfgang, BacsaJorge, de BritoWolfgang, BlumJorge, EscorihuelaWolfgang, DornischJorge, FeuchtwangerWolfgang, FrickeJorge, MorgadoWolfgang, KernJorge, Paz FerreiroWolfgang, KleemannJorge, PeixinhoWolfgang, LöserJos, BrouwersWolfgang, WeigandJosé A. F. O., CorreiaWolfram, VolkJosé A., HuidobroWondwosen Abebe, AregawiJose A., LoyaWondwosen, MetaferiaJosé A., MartinsWon-Ik, ChoJose A., Sainz-AjaWon-Jun, ShonJosé Alberto, ÁlvarezWonku, KangJosé António, CovasWon-Seok, KoJosé Antonio, Reglero RuizWon-Suck, OhJose Antonio, Travieso-RodriguezWoo June, ChoiJosé Barroso, De AguiarWoo-Jae, SeongJose Carlos Martins, De AlmeidaWookjin, LeeJosé Carlos Rodríguez, HernándezWookyung, SunJose Florentino, Álvarez-AntolínWoong, NamJosé Gaspar, MartinhoWooseok, JiJosé Ginés, Hernández-CifreWoosung, ParkJosé Humberto, Almeida JúniorWouter, HerreboutJosé Ignacio, IribarrenWouter, Van RenterghemJosé Ignacio, LombrañaWurong, JianJosé J., BaldovíXanthippi, ChatzistavrouJosé L., Amaro-MelladoXavier, BalandraudJosé Luis, Calvo GuiradoXavier, BegaudJosé Luis, Cantero GuisándezXavier, ColinJosé Luis, García CalvoXavier, ColomJosé Luís, Gómez-RibellesXavier, LegrandJosé Luis, Montaño PriedeXenofon, StrakosasJose M., SanchezXi, ShenJose Manuel, AmadoXialu, WeiJosé Manuel, BenitoXiaming, FengJose Manuel, DiazXian Hu, LiuJose Manuel, Gómez-SoberónXian, GuijunJosé Manuel, LópezXian, PengJosé Manuel, Moreno-MarotoXiang, ChenJose Manuel, Vasco-OlmoXiang, LiJosé Marcos, OrtegaXiang, MaJose María, FernandezXiang, YanJosé María, MontielXiang, ZhangJose Marqués, HuesoXiangang, LuoJose P. B., SilvaXiangfa, WuJosé Sebastián, Velázquez BlázquezXiangguang, MengJosé V., PrataXianghuai, DongJose Vicente, Ríos-SantosXianghui, ZhangJosé, AlemánXiang-Kai, YangJosé, AlvarezXiangning, BuJose, Calaf ChicaXiangwei, ZhuJose, CaridadXiangxiong, KongJosé, Duarte MarafonaXiangyang, DongJose, FernandezXiangyang, XuJosé, GamelasXiangying, ShenJose, Gamez-PerezXiangyu, ZhongJosé, GarciaXiangzhong, ChenJosé, Martínez-LilloXianhu, LiuJose, MillaXianhua, ChenJose, MunizXiao Dong, ZhouJosé, NevesXiao, ZhouJosé, PenuelasXiao-An, FuJosé, Rodríguez-AviXiaobo, ChenJose, SánchezXiaochao, TangJoseba, AlbizuriXiaochuan, XuJosef, FüsslXiaochun, ZhouJosef, JampilekXiaodong, WangJosef, JůzaXiaofeng, WangJosef, MarousekXiao-Guang, YueJosef, PetrusXiaohang, SunJosef, StráskýXiaohong, WangJosef, ZemekXiaohu, YaoJosefina, PonsXiaojian, GaoJosé-Maria, Cabrera-MarreroXiao-Lei, ShiJose-Maria, Delgado-SanchezXiaoliang, ZengJosep, Canet-FerrerXiaolong, JiaJoseph E., JakesXiaolong, ZhengJoseph Herbert, PodolskyXiaolong, ZhuJoseph N., GrimaXiaoming, HuangJoseph N., ZalamedaXiaoming, ZhangJoseph W. F., RobertsonXiaopeng, LiangJoseph, CameronXiaoqian, MaJoseph, DumontXiaoqing, WangJoseph, FurgalXiaosheng, FangJoseph, GovanXiaowen, ZhanJoseph, NdieyiraXiaoyan, ZhangJoseph, PoonXiaoyang, CaiJoseph, SloopXiaoying, ZhuangJoseph, TylczakXiaoyong, WangJose-Ramon, SarasuaXiaoyu, HuangJoshua, OwenXiaozhi, HuJoshua, YoungXifeng, LiuJosip, BrnićXigao, ChenJosip, IvšinovićXijia, WuJosip, KranjčićXijuan, ChenJosko, ViskicXijun, ShiJothi Saravanan, ThiyagarajanXin Li, PhuahJozef, BártaXin, HeJozef, DrabowiczXin, KangJozef, HaponiukXin, LiJózef, HorabikXin, LiuJozef, IwaszkoXin, MengJozef, JanovecXin, WangJozef, KeckesXin, WuJozef, KuczmaszewskiXin, XiaoJozef, MartinkaXin, XuJozef, MelcerXin, ZhangJozef, PeterkaXin, ZhuangJozef, StrečkaXinchang, ZhangJozef, ŠvajlenkaXincun, ZhuangJózsef, PapXingchen, LiuJuan A., SansXinghua, MengJuan Antonio, Almazán LázaroXingliang, DaiJuan Antonio, CeciliaXingwu, ZhangJuan B., Carda CastellóXinhong, WangJuan Carlos, Cabanelas ValcárcelXinlin, YanJuan Carlos, HernandoXinluo, ZhaoJuan Carlos, Pereira FalconXinming, LiJuan Carlos, Pomares TorresXinting, ZhengJuan F., MiravetXinxin, XiaoJuan Ignacio, BeltranXinxin, ZhangJuan Jesus, Martin-Del-RioXinyu, LiuJuan Jose, Galán-DíazXinyu, ZhaoJuan José, Santana RodríguezXiongqi, PengJuan Luis, Garcia-PomarXipeng, TanJuan M., Vazquez-MartinezXiuxuan, SunJuan María, García-LastraXizu, WangJuan Navarro, ArenasXosé Ramón, NóvoaJuan Pedro, FernándezXu, SongJuan, Ahuir-TorresXu, YangJuan, Aparicio-BlancoXu, ZhangJuan, Camara SerranoXu, ZhouJuan, García RodríguezXuan, YangJuan, JiménezXuchuan, JiangJuan, Monzó-CabreraXudong, CaoJuan, Niclos GutiérrezXue, ChenJuan, PouXue, LiJuan, ReyXuedong, XiJude, OsaraXuefeng, YaoJudit, HorváthXuefeng, ZhangJudita, GražulytėXue-Gang, ChenJudith, DawesXuehua, RuanJudyta, Cielecka-PiontekXuejian, WuJuergen, JakumeitXueliang, GeJuergen, SchreiberXueping, ZhangJuergen, SmolinerXuhui, ZhaoJugal Kishore, SahooXunchang, WangJuha, SongXuri, HuángJuhyuk, MoonXusheng, DuJu-Hyun, MunXxx, SedaoJuhyun, ParkYa, ChengJui-Chao, KuoYachao, WangJui-Cheng, ChangYadir, Torres HernándezJukka, KetojaYadira Bajon, FernandezJulfikar, HaiderYaewon, ParkJulia A., BaimovaYafeng, YangJulia Claudia, Mirza-RoşcaYahya, MezianiJulia, Alvarez-MalmagroYajun, ZhouJulia, BujanYakov, StrelnikerJulia, LorenzoYaming, PanJulia, PribylYan V., ZubavichusJulia, UreñaYan, BusbyJulián Jiménez, ReinosaYan, DongJulian, KubisztalYan, HanJulian, PilzYan, HongJulian, PlewaYan, WuJulián, PuszkielYan, ZhangJulian, RosalieYan, ZhugeJulian, UnglaubYandong, JiaJuliána, DrábikováYanfei, GaoJuliana, SchellYanfeng, WangJuliane, Salbach-HirschYang, HuJulie, ZhangYang, LiJulien G., MahyYang, LinJulien, BertheYang, LiuJulien, CapelleYang, LuJulien, FérecYang, SongJulien, LegrosYang, WangJulien, MahyYang, YuJulien, VieillardYang, ZhangJulio, Gutiérrez MorenoYang-Hee, KwonJulio, Ramirez-CastellanosYanghoo, KimJulita, Dworecka-WójcikYang-Kao, WangJulius, SchoopYanhua, LeiJuliusz, WiniarskiYanhua, LuoJun Cong, GeYani, ZhaoJun, ChenYaniv, GelbsteinJun, KangYaniv, KnopJun, KoyanagiYanling, SchneiderJun, LiuYann, GarciaJun, NieYannick, CoffinierJunfei, ZhangYannick, PannierJung Gi, KimYannick, SalaminJung Gu, LeeYanquan, GengJung Heum, YeonYanzi, GaoJung Jin, KimYao, ShunJung Sang, ChoYaodong, LiuJunghwan, KimYapu, ZhaoJung-Kul, LeeYaqiao, WuJungwon, HuhYarusova Sofya, BorisovnaJunhui, JiYashar, BehnamianJunhui, ShiYashar, Esfahani MonfaredJun-Hyun, KimYashwanth Kumar, BandariJunjie, WangYasin Emre, DurmusJunjie, ZhuYasuhiko, TabataJunko, AimiYasuhisa, AdachiJunkyeong, KimYasuko, MoriyamaJunlei, TangYasunori, AyukawaJunling, HuYasunori, MinamiJunlong, ShangYasushi, ShimadaJunmin, LiuYasutaka, AndoJunren, LinYasutaka, KitahamaJunseop, LeeYauheni, ZhalniarovichJunsheng, LiYavuz, CaydamliJunyan, YiYe, TianJunyi, LiuYe, TingJunyong, ParkYee Yan, LimJuozas, ValivonisYeon Ju, KimJuraj, GerliciYeou-Fong, LiJure, ŽigonYevgen, GorashJurek, KwelaYevgeny, VapnikJurga, JuodkazytėYi Sheng, LaiJürgen, BriegerYi, BaoJürgen, IhlemannYi, DengJurgita, MalaiškienėYi, NieJurgita, ZekonyteYi, YangJüri, MajakYi-Chen, LiJurij J., SidorYi-Chun, ChenJurijs, DekhtyarYifan, LiJürn W. P., SchmelzerYifeng, HuangJustas, BražiūnasYi-Huang, HsuehJustin B., SamburYijie, ZhangJustyna, DzięciołYi-Jun, XuJustyna, KasińskaYimei, WenJustyna, KozlowskaYiming, JiangJustyna, Kucinska-LipkaYin, YinJustyna, Kucińska-LipkaYinan, CuiJustyna, Kulczyk-MaleckaYing, CaoJustyna, KuziakYing, HuangJustyna, StrankowskaYing, LiJustyna, SulejYing, YangJustyna, SułowskaYingang, LiuJustyna, ZygmuntowiczYingchao, SuJyh-Herng, ChenYingchao, YangJyh-Shiarn, CherngYing-Chih, LiaoJyotsna, SharmaYing-Chih, PuK., KenryYing-Guo, ZhouKaarlo, NieminenYinghao, MiaoKadir, OzaltinYinglei, LiKae-Long, LinYing-Liang, ChenKagan, KermanYingsheng, ChengKah Hou, TengYingwei, YangKahraman, KeskinboraYingxiang, LiuKai Chun, LiYingxin, HeKai Min, YangYingzi, YangKai S., ExnerYi-Ning, ChenKai Yuan, LiYinnian, FengKai, GongYinyin, BaoKai, OßwaldYi-Ping, FangKai, TaoYiping, WangKai, TreutlerYiqun, ZhengKai, WangYiqun, ZhouKai, WeiYiran (Emma), YangKai, WuYisong, TanKai, XiYit Lung, KhungKai, YanYiting, XuKai, YinYi-Tsu, ChanKaikai, SongYiwei, WengKailiang, XuYi-Wen, ChenKaimiao, LiuYiwen, LiKainan, WangYixuan, FengKajetan, MüllerYlenia, ZambitoKakeru, FujiwaraYo, TomotaKállai, NikolettYoav, LinzonKalliopi, KousiYogendra Kumar, MishraKallol, PradhanYogendra, MishraKamal, HossainYogesh K., VohraKamala Kanta, NandaYohei, JinnoKambiz, ChizariYoichi, NakajimaKamil, DziubekYoichi, YamashitaKamil, KaminskiYoji, ShibutaniKamil, KaszycaYoko, Yamabe-MitaraiKamil, KowalskiYolande, MuratKamil, RahmeYonatan, CalahorraKamil, ZalegowskiYong Chan, ChoiKamila, SadowskaYong Jae, SuhKamila, SałasińskaYong Jin, JeongKamran, KardelYong Jun, ChoiKamran, MakarianYong Soo, YoonKamyar, Shirvani MoghaddamYong X., GanKan, ZhangYong, DingKanchana, KularatneYong, HaoKandammathe V., SreekanthYong, LiuKandasamy, SaravanakumarYong, SunKang Su, KimYong, ZhangKang, YaoYongbeom, SeoKanglin, XingYongchao, MouKanji, OnoYong-Cheng, LinKanwal, ChadhaYongcun, ZhangKap Jin, KimYongda, YanKapil, GangwarYonggang, PangKapil, PatelYonggang, ShangguanKar Wey, YongYong-Hak, LeeKarathanasopoulos, NikolaosYonghee, KimKarekin, EsmerianYong-Jie, HuKarel, DvořákYongjun, FengKarel, SakslYongqian, ShiKaren, EsquivelYong-Seok, LeeKarim, AlyYongsheng, GaoKarim, CherkaouiYoo, Myong JaeKarimulla, MullaYoo-jae, KimKarin, Habermehl-CwirzenYoon-Hwae, HwangKarin, LeistnerYooseob, SongKarin, PopaYooseok, ShinKarine, RehelYoshiaki, AdachiKarl, BunneyYoshiharu, KimuraKarla, MerazzoYoshihiko, NakataniKarla, Quiroz-EstradaYoshihisa, FujiiKarl-Christian, ThienelYoshihito, HayashiKarol, ĆwiekaYoshiki, MikamiKarol, JaśkiewiczYoshimasa, YamamotoKarol, KyziolYoshinori, NarutaKarol, StrzałkowskiYoshitaka, NakanishiKarol, SynoradzkiYoshitaka, NaraKarol, SzałowskiYoshitake, MasudaKarol, SzubertYoshito, AndoKarol, WolskiYosuke, GotoKarolína Machalová, ŠiškováYosuke, KageshimaKarolina, Goździewska-HarłajczukYoufeng, YueKarolina, GrabowskaYougen, YiKarolina, JakubczykYouhei, TakedaKarolina, SyrekYouhong, TangKároly, BenedaYoumin, RongKarsten, GüntherYoun-Bae, KangKarsten, MüllerYoung Min, SongKarsten, StahlYoung Sik, KimKarthik, RathinamYoung Sik, PyunKarthik, TappaYoung-Chul, LeeKarthikeyan, GnanasekaranYoungeup, JinKartik, BeheraYoung-Hag, KohKarunakaran, GopaluYounghan, YoonKarwowska, BeataYoung-Hwan, YouKaryn, JarvisYoung-Il, JangKashi Vishwanath, JessuYoungjae, ChunKasim, KorkmazYoungjun, SongKasimayan, UmaYoung-Jun, YouKaspar M. B., JansenYoung-Kwon, ParkKasper, StokbroYoung-Min, KangKasper, Van GasseYoungmin, KimKasturi, Joshi-NavareYoung-Min, KimKasturi, RoyYoung-Seob, JeongKatakalos, KonstantinosYoung-Soo, SeoKatariina, PussiYoung-Su, LeeKatarina, MarušićYounho, RewKatarina, MonkovaYousheng, ZouKatarzyna Miłkowska-PiszczekYoushi, HongKatarzyna, ArkuszYoussef, HamidiKatarzyna, BalinYousub, LeeKatarzyna, BialasYouwen, ZhangKatarzyna, Cesarz-AndraczkeYu (Tom), GaoKatarzyna, CieślakYu Kyoung, RyuKatarzyna, Dolzyk-SzypcioYu M., SpivakKatarzyna, GabryśYu Ying (Clarrisa), ChoongKatarzyna, GawlińskaYu, CherepennikovKatarzyna, JankowskaYu, HoriuchiKatarzyna, JanuszewiczYu, LiKatarzyna, JedynakYu, LiuKatarzyna, JelonekYu, QiaoKatarzyna, Kalinowska-WichrowskaYu, SekiguchiKatarzyna, KlimekYu, SongKatarzyna, Kowalczyk-GajewskaYu, WangKatarzyna, Leszczyńska-SejdaYu, YanKatarzyna, LewandowskaYu, ZhongKatarzyna, MrózYuan, RenKatarzyna, Niemirowicz-LaskowskaYuan, ZhangKatarzyna, NowinskaYuan-bo, ZhangKatarzyna, RajkowskaYuan-Chang, LiangKatarzyna, Siwińska-CiesielczykYuanchao, LiKatarzyna, SkórczewskaYuancheng, FanKatarzyna, StaszakYuan-Jen, ChangKatarzyna, WilpiszewskaYuankun, LinKatarzyna, ZawadaYuanshen, QiKate, NguyenYuanwei, YanKaterina, GiouriYuanyu, HuangKaterina, MouralovaYuanyuan, LiKaterina, SojkovaYuBin, ChenKatharina, AlbrechtYubin, ZhangKatharina, PrinzYubo, JiaoKatharine Moore, TibbettsYucheng, FuKatherine M. E., StewartYu-Chih, ChiangKatherine, AristizabalYu-Chun, ChiangKatherine, BrownYue, HouKatherine, HooperYue, HuangKathrein, Von KopylowYue, XiaoKathrin, SpendierYue, ZhaoKati, RajuYueh-Hsun Kevin, YangKatia, GuerinYueh-Lien Robbie, LeeKatica, SimunovicYuen, ClementKatie, AldredYuesheng, GaoKatir, ZioucheYuewu, WangKatogi, HideakiYufan, ZhaoKatrin, BokelmannYu-His, HuangKatrin, WudyYuh-Ping, ChangKatsuaki, TanabeYu-ichi, KomizoKatsuhiko, ArigaYuichiro, MatsushitaKatsushi, TanakaYuji, AraiKauko, LeiviskäYukari, OdaKaupo, KukliYuki, AkagiKaushik, ParmarYuki, SugiuraKaushik, SankarYukihiro, HaradaKaveh, AhadiYukihiro, MatsumotoKavian, CookeYukinari, SunatsukiKavita, MathurYukio, HamaKavitha, PathakotiYuko, TadaKay-Peter, HoyerYulia M., PanchenkoKazek-Kęsik, AlicjaYulia N., BespalkoKazem, JadidiYulia Yu., BozhkoKazimierz, FabisiakYulia, PushkarKazimierz, KowalskiYuling, NiuKazuaki, KisuYulong, LiKazuhiko, HashimotoYuman, ZhuKazuhiko, KitamuraYuming, TangKazuhiro, KandaYun Yong, KimKazuki, YoshiiYun, HeKazunari, YoshidaYunbo, BiKazunori, TsubakiYunfeng, GeKazuo, ShimizuYung, TingKazuo, TakahashiYung-Ching, DingKazutoshi, KojimaYung-Chou, KaoKazuyuki, HokamotoYung-Chung, ChenKe, HanYung-Jin, WengKee Sung, LeeYunlong, WuKeerti, Keerti KappagantulaYunming, WangKefeng, XieYunwen, TaoKei, ShibataYunyan, ZhangKeiji, JindoYuri, GornostyrevKeiji, OgawaYuri, MikhlinKeiji, ShigaYuri, RibakovKeita, AndoYuri, SharkeevKeith J., StineYuri, SvirkoKeith, FriedmanYuri, VoznyakKeith, RochfortYurii, Gun’koKejia, YangYurii, SharkeevKejing, YuYuriy, GarbovskiyKelvin, AfrashtehfarYurong, HeKen, ShiratoYurong, MaKen’ichiro, MatsumotoYury A., SkorikKenan, SongYury M., BelousovKendra, ErkYury, KapelyushinKeng-Shiang, HuangYusheng, LeiKenichiro, NakaraiYusheng, ShiKenji, KatohYusuf A., MehtaKenji, KinashiYusuke, HiejimaKenji, SugisakiYusuke, TakahashiKenji, UsuiYusuke, YamauchiKenjiro, YazawaYuta, MatsushimaKenneth R., PoeppelmeierYuta, NabaeKenneth, ScheplerYutaka, OhsedoKenta, AoyagiYu-Ting, TsaiKentaro, HozumiYu-Tse, WuKentaro, YoshidaYuxiang, HongKeon-Soo, JangYuxin, HeKerstin, ZehbeYuzo, NakamuraKeshav Raj, PaudelYves, GossuinKetij, MehulićYves, HuttelKeun Ba Da, SonYves, PiponKeun-Soo, KimYvonna, JiráskováKevin J., McHughZacharias, FrontistisKevin, CavicchiZacharias, ViskadourakisKévin, CocqZacharoula, IatridiKevin, CritchleyZachary, HarrisKevin, De FranceZachary, SmithKevin, MontagneZaharia, Sebastian MarianKevin, SmithZahra, AbdollahnejadKeyou, MaoZahra, BazrafshanKhader, HamdiaZahra, El-SchichKhadija El, CheikhZahra, GholmaiKhaled, AlalussZahra, KhatamiKhaled, GiasinZahra, Mazrouei-SebdaniKhaled, HabibZahran, Al-KamyaniKhaled, MurtadaZahyun, KuKhaled, RagabZain, Ul-AbdinKhaled, SinjabZaiqing, QueKhalid, ArifZaiyuan, LeKhalid, ElaminŻaneta Anna, MierzejewskaKhalid, ElwakeelŽaneta, DohnalováKhalid, HashimŻaneta, Król-KilińskaKhalid, RashidZaneta, SwiatkowskaKhanh Chau, LeZao, YiKhanh, NguyenZara, SusiKheng-Lim, GohZarghaam Haider, RizviKhizir, MahmudZbigniew, BrytanKhondokar Mizanur, RahmanZbigniew, GarncarekKi-buem, KimZbigniew, GiergicznyKidane Fanta, GebremariamZbigniew, GronostajskiKien-Wen, SunZbigniew, KabalaKim, BongjuZbigniew, MarciniakKimihiro, SakagamiZbigniew, NadolnyKimitoshi, YagamiZbigniew, OksiutaKinga, BociongZbigniew, OtrembaKinga, KorniejenkoZbigniew, PaterKinga, PielichowskaZbigniew, PedzichKinga, RodakZbigniew, PozorskiKinga, SzentnerZbigniew, RanachowskiKingshuk, DuttaZbigniew, RespondekKioupis, DimitrisZbigniew, StempienKira E., VostrikovaZbyněk, KeršnerKiran, ChereddyZbynek, RaidaKiril, KrezhovZbyšek, PavlíkKirill I., RybakovZdenek, ChlupKirill M., SkupovZdeněk, KošnářKirill V., YusenkoZdeněk, PokornýKirnbauer, AlexanderZdenek, RemesKirtika, KohliZdzisław, NowakKiryl D., PiatkevichZdzisława, OwsiakKishan, MahmudZe’ev, PoratKivovics, MártonZeinab, EzzeddineKiyofumi, KurumisawaŽelimir, JelčićKiyofumi, TakabatakeZeljka, PetrovicKlára, BeranováŽeljko, SkokoKlara, KraljićŽeljko, VerzakKlara, MagyariZeman, OliverKlára, MelánováZen, MaenoKlaus Dieter, SchierbaumZengfeng, ZhaoKlaus Schütt, HansenZengqian, ShiKlaus, BöningZengshun, ChenKlaus, FriedrichZenon, MrózKlaus, HolschemacherZeyi, GuanKlaus, KöhlerZhangwei, WangKlaus, RischkaZhao, DingKlaus, SchrickerZhao, GuangjiuKlaus, StöweZhao, WangKlaus, SzielaskoZhaochao, LiKlaus, TimmelZhaogang, YangKlaus, VoitZhaohui, LiKłosowski, PawełZhaoyang, DingKoen, RobeynsZhe, ChenKogularasu, SakthivelZhe, GaoKoh, SugamataZhe, GuiKoh-ichi, SugimotoZhe, ZhangKoichi, MayumiZhejun, WangKoichi, TakakiZheling, LiKoichiro, NambuZhenfeng, GongKoizumi, ToshioZheng, CuiKoji, GotohZheng, ZhangKoji, NakabayashiZhengjie, FanKojiro, UetaniZhenglong, LeiKokot, GrzegorzZheng-mao, ChenKonda Gokuldoss, PrashanthZhengming, HuangKongchang, WeiZhengwu, JiangKonrad Jerzy, KapciaZhengxu, CaiKonrad, GruszkaZhengying, WeiKonrad, MollenhauerZhenlong, WangKonstantin A., ProsolovZhenping, WanKonstantin P., AndryushinZhenyin, HaiKonstantin, BorodianskiyZhenyuan, JiaKonstantin, BurmistrovZhenzhou, WangKonstantin, KatinZheshuai, LinKonstantin, KhivantsevZhezhen, FuKonstantin, PopovZhi, YangKonstantin, TretiakovZhibin, DuKonstantinos G., KalogiannisZhibo, YangKonstantinos G., MegalooikonomouZhichao, LouKonstantinos I., ChristidisZhidong, ZhangKonstantinos P., GiannakopoulosZhifu, ZhouKonstantinos, BeltsiosZhi-Gang, GuKonstantinos, DimosZhigang, XiaoKonstantinos, MountrisZhigang, ZangKonstantinos, PapoutsisZhihua, XiongKonstantinos, RaftopoulosZhihuan, WengKonstantinos, SakkasZhijian, WangKonstantinos, SenetakisZhijie, WangKonstantinos, SidiropoulosZhilin, LiuKonstantinos, SotiriadisZhiqiang, LiangKonstantinos, TsongasZhiqiang, ShenKontu-Druga, EvangelismZhiqiang, ZhangKoo-Hyun, ChungZhishun, WeiKornel, MajlingerZhitian, LiuKornel, PietrzakZhitong, ChenKornelius, TetznerZhiwei, LinKosmas, EllinasZhixia, LiuKosuke, NozakiZhixing, GanKotaro, DoiZhiyuan, FengKotaro, MoriZhiyuan, QiKothandam, GopalakrishnanZhong, LiKoumei, BabaZhong, WuKouun, ShiraiZhong, ZhengKozo, MatsumotoZhongmin, JinKresimir, GrilecZhongmin, XiaoKris, CampbellZhongming, FanKrishna, Nama ManjunathaZhou, YeKrishnan, MurugappanZhouying, HeKristaq, GazeliZhu, PanKristen M., MeiburgerZhu, WeiKristian, KnihaZhujin, YangKristin, CarpenterZhuo, WangKristin, PfeifferZhusheng, ShiKristina, GorsetaZhuxian, ZhouKristina, KonstasZhuyuan, QiKristýna, VavrušováZi, OuyangKrisztina, LászlóZiad, MoumniKrunoslav, JuraicZiaudding, KhanKrystyna, MalińskaZichen, LuKrzysztof Adam, OstrowskiŽiga, GosarKrzysztof Artur, BogdanowiczZiga, SmitKrzysztof J., KurzydłowskiZigmas, BaleviciusKrzysztof, DudekZima, BeataKrzysztof, EjsmontZingway, PeiKrzysztof, FormelaZiniu, YuKrzysztof, GórnyZinoviy, BlikharskyyKrzysztof, GromyszZiyong, HouKrzysztof, GrysaZlata, Dolacek-AldukKrzysztof, GrzelakZlata, Hrnjak-MurgićKrzysztof, KarczewskiZlatin, ZlatevKrzysztof, KlugerZoe, TerzopoulouKrzysztof, KoniecznyZohaib, KhurshidKrzysztof, KoronaZohreh, VafapourKrzysztof, KulaZois, SyrgannisKrzysztof, LejcuśZois, TsinasKrzysztof, MaciejewskiZoltán, DudásKrzysztof, MiecznikowskiZoltan, HomonnayKrzysztof, MiernikZoltán, KovácsKrzysztof, MoraczewskiZoltán, WeltschKrzysztof, MuszkaZong-Liang, TsengKrzysztof, NadolnyZongshan, ZhaoKrzysztof, NaplochaZrinka Buhin, SturlicKrzysztof, NowackiZsófia, KeresztesKrzysztof, OstrowskiZsolt, CziganyKrzysztof, PańcikiewiczZsolt, KovácsKrzysztof, PielichowskiZuosheng, LeiKrzysztof, RadwańskiZuoti, XieKrzysztof, RogowskiZuquan, JinKrzysztof, RokoszZuzana, FlorkováKrzysztof, SchabowiczZuzana, JurasekovaKrzysztof, SiczekZuzana, KoledovaKrzysztof, SkrzypkowskiZuzana, MurčinkováKrzysztof, SołekZuzana, ZajíčkováKrzysztof, StrzelecZuzanka, TrojanovaKrzysztof, SzczepanowiczZuzanna, Żołek-TryznowskaKrzysztof, SzwajkaZvonimir, DadićKrzysztof, TadyszakZygmunt Mariusz, GusiatinKrzysztof, WałęsaZygmunt, Mikno

